# Computation‐Guided Control of Excited‐State Deactivation through Modulation of Conical‐Intersection Accessibility by Donor–Acceptor Asymmetry in Bridged Stilbene AIE Luminogens

**DOI:** 10.1002/advs.76058

**Published:** 2026-06-18

**Authors:** Takuya Tanaka, Satoshi Suzuki, Hirosato Koyanagi, Kiyoshi Miyata, Riki Iwai, Kazunobu Igawa, Ken Onda, Ben Zhong Tang, Gen‐ichi Konishi

**Affiliations:** ^1^ Department of Chemical Science and Engineering Institute of Science Tokyo Meguro‐ku Tokyo Japan; ^2^ Department of Chemistry Faculty of Science Kyushu University Nishi‐ku Fukuoka Japan; ^3^ Faculty of Advanced Science and Technology Kumamoto University Kumamoto Japan; ^4^ Guangdong Basic Research Center of Excellence for Aggregate Science, School of Science and Engineering, Shenzhen Institute of Aggregate Science and Technology The Chinese University of Hong Kong (Shenzhen) Shenzhen China

**Keywords:** aggregation‐induced emission, conical intersection, molecular design, potential energy surface, topology

## Abstract

A computation‐guided investigation of asymmetric donor–acceptor bridged stilbenes reveals structure–property relationships governing conical intersection (CI) accessibility and excited‐state deactivation in aggregation‐induced emission (AIE) luminogens. Although CIs play a central role in nonradiative decay, the molecular factors governing CI accessibility in AIE systems remain insufficiently understood. Here, we show that asymmetric donor–acceptor placement combined with bridge‐controlled structural flexibility strongly influences CI accessibility and excited‐state deactivation in push–pull alkylene‐bridged stilbenes ([6]/[7]). Quantum‐chemical analyses of 30 derivatives reveal substituent‐dependent energetic trends associated with CI accessibility that can be rationalized by the relative energetic positions of the Franck–Condon and CI geometries. On the basis of these trends, representative derivatives, including DCBS[6], DCBS[7], DPB[7]C, and DPB[7]N, were synthesized together with reference compounds. Their photophysical properties generally correlate with the computed CI‐accessibility trends, indicating that donor–acceptor asymmetry and bridge rigidity cooperatively influence excited‐state deactivation and fluorescence suppression in solution. Time‐resolved spectroscopy, post‐relaxation PES analyses, and CI topology analyses further support the proposed relaxation pathways and suggest that substituent inversion alters CI energetics, topology, and nonadiabatic coupling. These findings provide mechanistic insight into substituent‐dependent excited‐state deactivation in bridged stilbenes.

## Graphical abstract

Donor–acceptor asymmetry modulates conical‐intersection accessibility and topology in bridged stilbenes, providing mechanistic insight into excited‐state deactivation and aggregation‐induced emission (AIE).

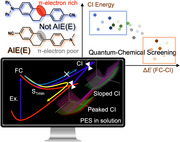

## 1 Introduction

Aggregation‐induced emission (AIE) luminogens (AIEgens) have emerged as an important class of photofunctional chromophores since Tang and co‐workers first introduced the concept [1, 2]. Over the past two decades, AIEgens have enabled diverse photonic and materials applications [3, 4], spanning fields from organic light‐emitting diodes (OLEDs) [5] and functional soft materials [6, 7, 8, 9] to photodynamic therapy [10, 11, 12] and microbial sensing [13]. Representative examples of AIEgens include tetraphenylethene [14, 15, 16], hexaphenylsilole [17], and 9,10‐bis(dialkylamino)anthracene [18, 19, 20]. Accumulating mechanistic and theoretical studies indicate that fluorescence quenching in solution arises from ultrafast internal conversion mediated by large‐amplitude structural distortions that transiently disrupt the π‐conjugated system. Quantum‐chemical analyses further suggest that this nonradiative decay proceeds through conical intersections (CIs) on the potential energy surface (PES) [21, 22, 23, 24]. In contrast, in the solid state, restricted molecular motions destabilize these CIs and enable radiative decay from the S_1_ minimum. Although the energetic accessibility of CIs is increasingly recognized, most AIEgens were not originally designed with this concept in mind but were discovered empirically.

**FIGURE 1:**
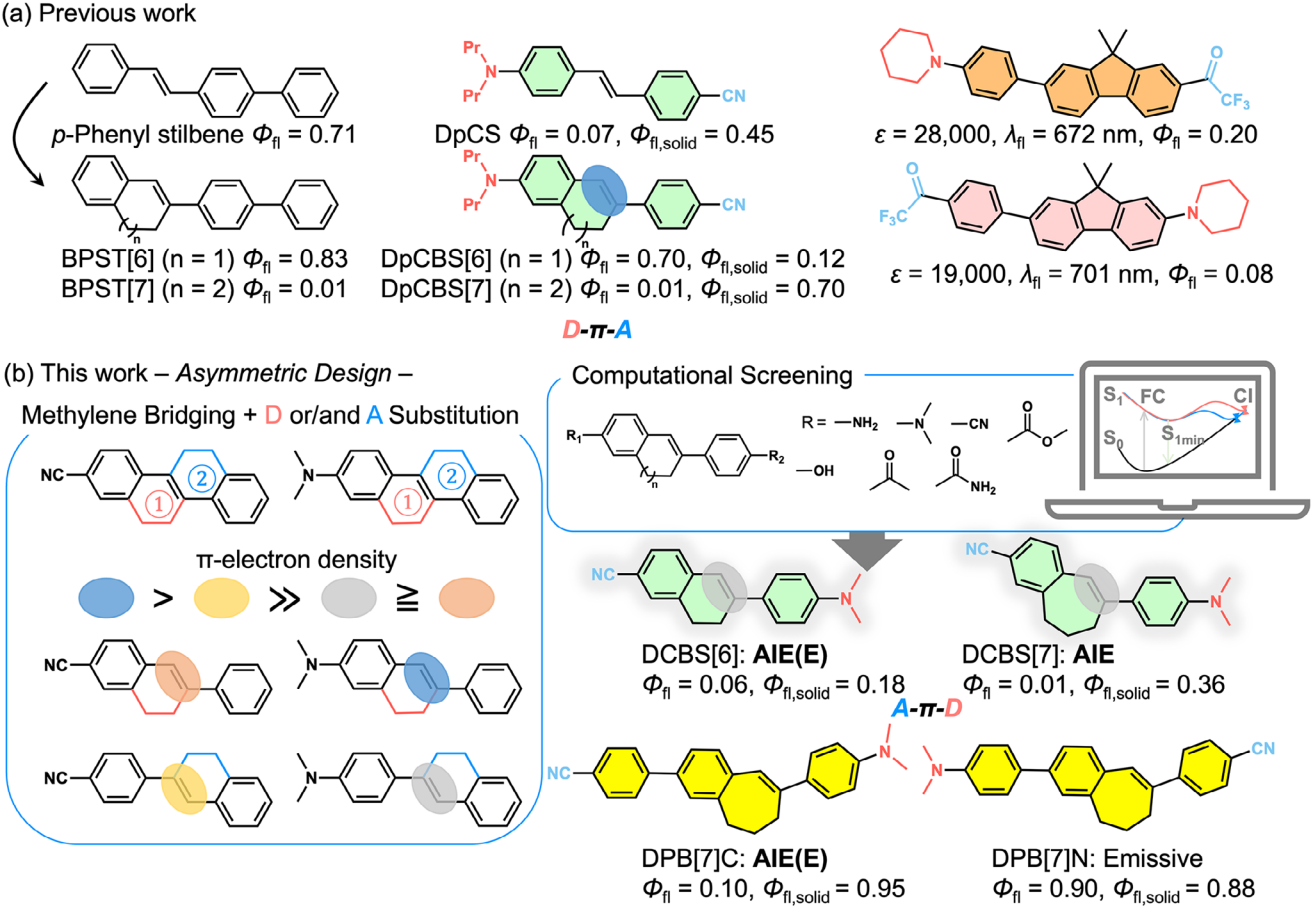
(a) Previous work: Design of AIE‐active bridged stilbenes and substituent‐position–dependent modulation of photophysical properties on asymmetric π‐scaffolds [25]. (b) This work: Asymmetric structural modification combining alkylene bridging (ethylene for [6] and propylene for [7]) with donor or acceptor substitution, leading to redistribution of π‐electron density on the central C_et_–C_et_ bond (left). Comparative computational analysis of substituent‐dependent CI accessibility enabling comparative investigation of new bridged stilbenes (right). Chemical structures of the newly developed AIE, AIEE, and emissive candidates are shown. Fluorescence quantum yields (*Φ*_fl_) were measured in THF.

To investigate molecular factors governing CI accessibility and nonradiative decay, we integrated theoretical insights with structural organic chemistry [25]. As a mechanistic starting point, we focused on stilbene, a prototypical chromophore known to exhibit efficient internal conversion in solution [26, 27, 28, 29, 30, 31, 32]. We hypothesized that conjugation extension and functional‐group modification of a scaffold prone to efficient nonradiative decay could generate systems exhibiting aggregation‐induced emission behavior. Although π‐extended stilbene (PST) shows visible solid‐state fluorescence, it remains emissive in solution; however, introducing flexible alkylene bridges between the central C═C bond (C_et_–C_et_) and the phenyl rings facilitates access to low‐lying CI regions associated with nonradiative decay in solution, converting PST into an AIEgen (BPST[7], Figure 1a) [25]. More recently, we developed a bridged derivative of the weakly emissive 4‐dimethylamino‐4’‐cyanostilbene (DCS), yielding a D–π–A‐type system exhibiting AIE behavior (DpCBS[7]) that undergoes efficient nonradiative relaxation irrespective of solvent polarity (Figure 1a) [33]. Time‐resolved spectroscopy revealed nearly constant activation barriers across solvents, indicating solvent‐dependent energetic shifts of the PES without alteration of the deactivation mechanism. These findings prompted us to investigate how electronic asymmetry and bridge rigidity cooperatively influence CI accessibility within D–π–A‐type bridged stilbene systems.

Recently, we found that transposition of donor and acceptor positions in asymmetric π‐extended fluorenes exhibiting solvatochromic fluorescence (i.e., D–π–A vs. A–π–D) induces pronounced changes in absorption coefficients, emission wavelengths, and fluorescence quantum yields (Figure 1a, right) [34, 35]. From a theoretical standpoint, asymmetric substitution patterns have been reported to significantly alter excited‐state PES energetics; notably, Schuurman et al. demonstrated that electron‐donating and electron‐withdrawing substituents systematically modulate CI energies in ethylene [36, 37]. In bridged DCS derivatives, donor and acceptor units can be arranged in inverted orientations relative to the alkylene bridge, thereby introducing steric and electronic asymmetry around the C_et_–C_et_ bond. We hypothesized that such asymmetry would induce asymmetric π‐electron distribution along the C_et_–C_et_ bond, lowering the energetic cost of twisting distortions and thereby facilitating access to low‐lying CI regions (Figure 1b, left). In addition to substituent asymmetry, bridge size is expected to strongly influence excited‐state deactivation behavior through modulation of structural flexibility. In particular, comparison between the relatively rigid six‐membered scaffold and the more flexible seven‐membered analogue enables examination of how substituent‐dependent electronic effects emerge under different degrees of geometric constraint.

In this study, we employ comparative quantum‐chemical PES analyses to investigate how donor–acceptor asymmetry and bridge rigidity influence excited‐state relaxation and nonradiative decay in bridged stilbenes. We performed systematic PES calculations on structurally diverse six‐ and seven‐membered bridged stilbenes bearing donor, acceptor, and donor–acceptor substituents, and analyzed how the excited‐state PES responds to substituent type, placement, and bridge rigidity. On the basis of these substituent‐dependent energetic trends, representative derivatives, including DCBS[6], DCBS[7], DPB[7]C, and DPB[7]N, were synthesized and investigated together with reference compounds. Their experimentally observed photophysical properties were then compared with the computed CI‐accessibility trends using steady‐state spectroscopy, fs‐transient absorption spectroscopy, and CI topology analyses. Together, these results provide mechanistic insight into substituent‐dependent excited‐state deactivation in bridged stilbene systems.

## 2 Results and Discussion

We first employ comparative computational analyses to explore how bridge size and substituent placement at the X and Y positions affect CI accessibility in bridged stilbenes through changes in the relative energetics of the Franck–Condon and CI regions. On the basis of these trends, representative D–π–A derivatives are investigated to examine whether the computed energetic trends are reflected in experimentally accessible luminogens. Their photophysical properties in solution and the solid state are then investigated, followed by fs‐transient absorption spectroscopy and CI topology analyses to evaluate how the computed energetic trends are reflected in excited‐state deactivation and nonradiative decay. This sequence of analyses provides a framework for discussing how molecular structure, FC/CI energetic relationships, and photophysical behavior are interconnected.

### 2.1 Substituent‐Dependent Modulation of CI Accessibility

**FIGURE 2:**
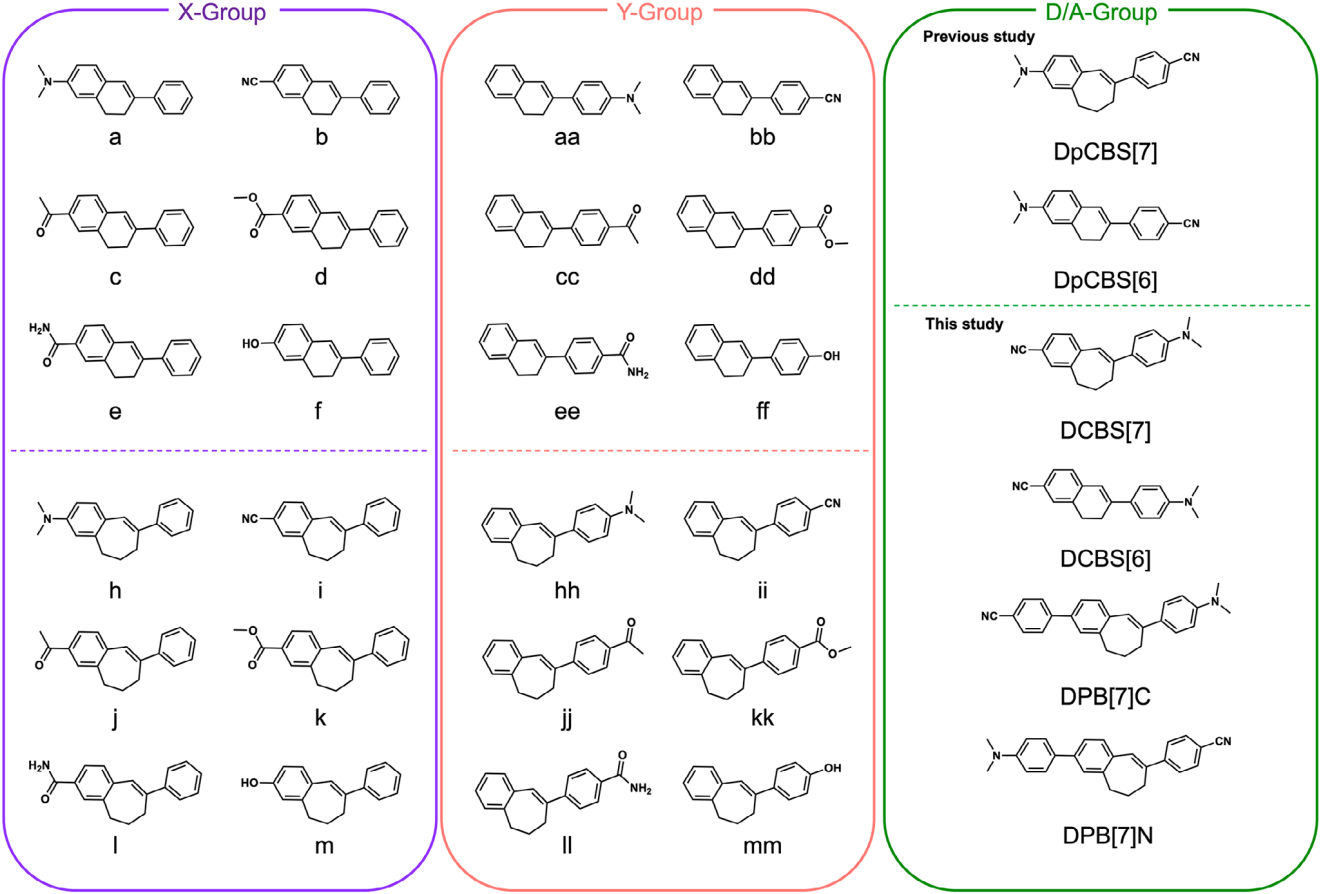
Molecular library used for comparative analysis of substituent‐dependent CI accessibility, classified into X‐Group, Y‐Group, and donor–acceptor (D/A) group derivatives.

To dissect how substituent identity and placement in bridged stilbenes (BST[*m*], *m* = 6,7) influence nonradiative deactivation, we constructed energy diagrams for the 30 compounds shown in Figure 2. We compared the relatively flexible seven‐membered scaffold (BST[7]), previously shown to promote AIE through enhanced CI accessibility, with the more rigid and planar six‐membered analogue (BST[6]). Because DpCBS[6] is already emissive in dilute solution, the rigid BST[6] scaffold provides a useful platform for examining how substituent‐dependent electronic effects influence CI accessibility under restricted geometric conditions. All relevant minima and CI geometries were computed using a unified theoretical protocol, as described below.

All CIs were optimized from pre‐twisted geometries around the C_et_─C_et_ bond using the branching‐plane update method [38]. Calculations were performed at the MRSF‐TDDFT level with the BHHLYP functional and the 6–31G(d) basis set (MRSF‐TD‐BHHLYP/6‐31G(d)) [39, 40, 41]. This approach affords a balanced description of excited‐state PESs by eliminating spin contamination inherent to conventional SF‐TDDFT while maintaining computational efficiency [39, 40]. The same computational protocol was consistently applied to all relevant stationary points, including the Franck–Condon (FC) point and the relaxed excited‐state minimum (S_1min_). All calculations were carried out using GAMESS (September 2022) [42]. The substituent set comprised electron‐donating groups (─OH, ─N(CH_3_)_2_) and electron‐withdrawing groups (─CN, ─COCH_3_, ─CONH_2_, ─COOCH_3_). Full computational details and energy diagrams are provided in Section S3 (Figures S1–S6).

From a theoretical standpoint, CI accessibility is ideally evaluated on the full excited‐state PES, including FC, the relaxed S_1min_, and pathways leading from these regions to the CI. Here, the relative energetic feasibility of reaching the CI region from the FC or S_1min_ regions is used as an operational measure of CI accessibility. However, such exhaustive mapping is impractical for systematic computational analyses. Previous quantum‐chemical studies have shown that the efficiency of nonradiative decay via CIs correlates strongly with the energetic relationship between the FC and CI regions [22, 24, 43, 44, 45, 46]. When the FC state lies higher in energy than the CI, the excess energy retained after photoexcitation enables barrierless or low‐barrier access to the CI, as exemplified by retinal and stilbene photoisomerization [47, 48, 49, 50]. Accordingly, the FC–CI energy difference (Δ*E* (FC–CI)), together with the absolute CI energy, provides physically meaningful and practical metrics for assessing CI accessibility. We therefore adopt these quantities to systematically evaluate substituent effects in bridged stilbenes.

**FIGURE 3:**
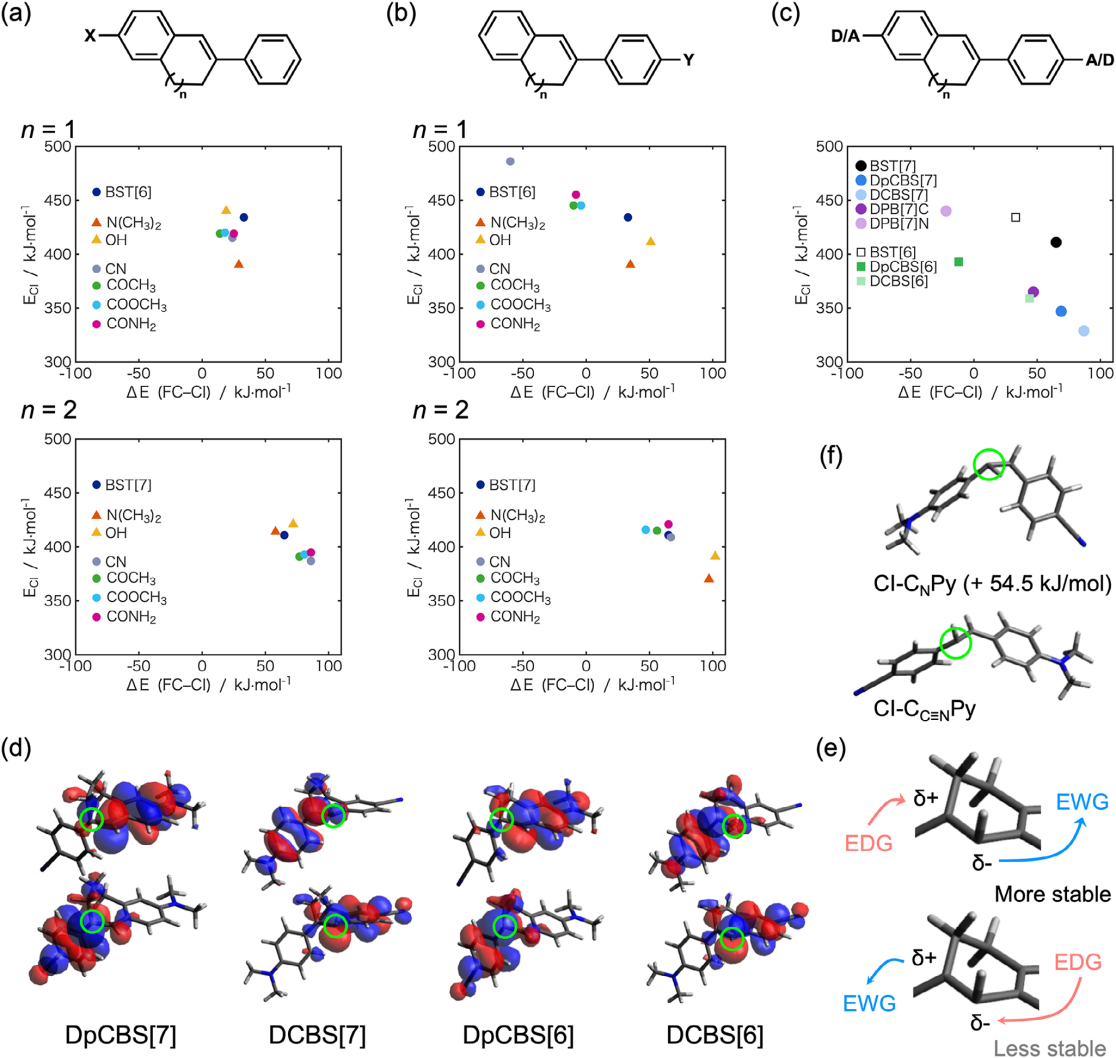
Computational substituent‐dependent trends in CI accessibility, plotted as the energy difference Δ*E* (FC–CI) versus CI energy (*E*_CI_) for (a) X‐position, (b) Y‐position, and (c) D–π–A derivatives. Molecules in the lower‐right region exhibit large Δ*E* (FC–CI) and a low *E*_CI_, indicating enhanced CI accessibility and thus promising candidates for AIE/AIE behavior. (d) Frontier molecular orbitals at the CI geometries of selected X‐substituted derivatives (LUMO, top; HOMO, bottom). Pyramidalized carbon atoms involved in the CI are highlighted. (e) Twisted pyramidal CI structures of DpCS: CI─C_C≡N_Py is ∼55 kJmol^−1^ more stable than CI‐C_N_Py. (f) Schematic illustration of the C_et_–C_et_ electronic structure and substituent‐dependent CI stabilization as a function of donor/acceptor arrangement.

Figure 3a,b summarizes the substituent‐dependent energetic trends for six‐ and seven‐membered bridged stilbenes (BST[6] and BST[7]). In the six‐membered system (*n* = 1), substituent effects at the X position are markedly weaker than in the seven‐membered analogue. Electron‐withdrawing groups (EWGs) lower the CI energy but concomitantly stabilize the FC energy, resulting in negligible changes in Δ*E* (FC–CI). Electron‐donating groups (EDGs) have only minor effects, with ─OH slightly destabilizing and ─N(CH_3_)_2_ weakly stabilizing the CI. In contrast, substitution at the Y position exhibits a clearer trend: EWGs (e.g., ─CN) substantially destabilize the CI and often render Δ*E* (FC–CI) negative, thereby creating an uphill FC→CI pathway and effectively suppressing CI access, whereas EDGs afford only modest stabilization. Overall, donor or acceptor substitution in BST[6] does not efficiently enhance nonradiative decay; EDGs provide limited effects, while EWGs—particularly at the Y position—strongly disfavor CI accessibility. In contrast, the seven‐membered system (BST[7], *n* = 2) exhibits a clearer and much stronger substituent dependence. At the X position, EWGs selectively stabilize the CI with little change in the FC energy, resulting in a substantial increase in Δ*E* (FC–CI) and enhanced CI accessibility, whereas EDGs have minimal impact. At the Y position, EDGs strongly stabilize the CI and increase Δ*E* (FC–CI), while EWGs induce only weak perturbations. These trends provide a useful guideline for BST[7]: the cooperative placement of EWGs at X and EDGs at Y maximizes CI accessibility, thereby contributing to modulation of nonradiative decay in D–π–A systems. Taken together, comparative analyses across both ring sizes suggest a consistent substituent‐dependent trend in CI accessibility within this bridged stilbene family: CI accessibility is most effectively enhanced by placing an EWG at the X position and an EDG at the Y position, which stabilizes the CI relative to the FC state in both ring systems examined here. On the basis of these substituent‐dependent trends, we next apply these substituent combinations to explicit D–π–A derivatives to examine how donor–acceptor interactions and bridge rigidity jointly govern CI accessibility.

In the present study, “CI accessibility” is used primarily to describe the relative energetic feasibility of reaching the CI region from the FC or relaxed S1 region. We note that the overall efficiency of nonradiative decay is additionally influenced by PES topology and nonadiabatic coupling around the CI. Although CI accessibility is primarily discussed here in energetic terms, the overall deactivation behavior is also influenced by PES topology and relaxation pathways.

### 2.2 CI Accessibility in Representative D–π–A Bridged Stilbenes

To examine the relationship between computed CI accessibility and photophysical behavior, we synthesized DCBS[6], DCBS[7], DPB[7]C, and DPB[7]N together with reference compounds. We examined five representative D–π–A derivatives (Figure 2) to elucidate how donor–acceptor (D/A) interactions and bridge rigidity cooperatively govern CI accessibility. Among these, 4‐dimethylamino‐4’‐cyano bridged[6]stilbene (DCBS[6]) and 4‐dimethylamino‐4’‐cyano bridged[7]stilbene (DCBS[7]) were selected as representative substitution patterns associated with enhanced CI accessibility. The previously reported 4‐dipropylamino‐4’‐cyano bridged[6]stilbene (DpCBS[6]) and bridged[7] analogue (DpCBS[7]) serve as reference non‐AIE and AIE‐active compounds, respectively [33]. In addition, DPB[7]C and DPB[7]N were introduced as π‐conjugation‐extended derivatives of DCBS[7] and DpCBS[7] to further probe the generality of the substituent‐dependent trends within this molecular framework. Figure 3c shows that the newly synthesized derivatives largely follow the substituent‐dependent trends identified at the X and Y positions. Based on Δ*E* (FC–CI), DCBS[6], DCBS[7], and DPB[7]C is predicted to exhibit enhanced CI accessibility relative to the non‐AIE reference DpCBS[6]. In contrast, DPB[7]N displays a negative Δ*E* (FC–CI) and a high CI energy, closely resembling the behavior of DpCBS[6].

### 2.3 Electronic Origin of CI Stabilization

To rationalize the substituent‐dependent modulation of CI geometries, we examined the electronic structures of the MECIs (Figure 3d). Although MRSF‐TDDFT describes the electronic structures of S_0_ and S_1_ using a few Slater determinants, namely |HOMO^2^>, |HOMO^1^LUMO^1^>, and |LUMO^2^>, it can still qualitatively capture the electronic character. The spatial distributions of the HOMO and LUMO provide insight into how substituents affect the MECI. At the twisted‐pyramidal MECI, the HOMO is localized on the pyramidalized carbon, whereas the LUMO is localized on the other carbon. Accordingly, placement of an electron‐withdrawing ─CN group on the pyramidalized carbon, which bears a negative charge, and an electron‐donating ─NMe_2_ group on the other carbon, which bears a positive charge, stabilizes the CI (Figure 3e). These results indicate that low‐lying CI is stabilized by the interplay between bridge‐induced geometric constraints and substituent‐directed electronic effects. To disentangle these contributions, we examined the unbridged D–A compound DpCS. Both C_et_ carbons undergo pyramidalization, yielding two distinct CI geometries (Figure 3f). The donor‐side pyramidalized CI is 54.5 kJ mol^−1^ higher in energy than the acceptor‐side analogue, mirroring the trend observed in the bridged systems and demonstrating that CI geometry is strongly influenced by substituent‐induced electronic effects. These results suggest that low‐lying CI geometries arise from the cooperative interplay between geometric constraint and electronic structure.

### 2.4 Relationship Between CI Accessibility and Post‐Relaxation Decay Pathways

**FIGURE 4:**
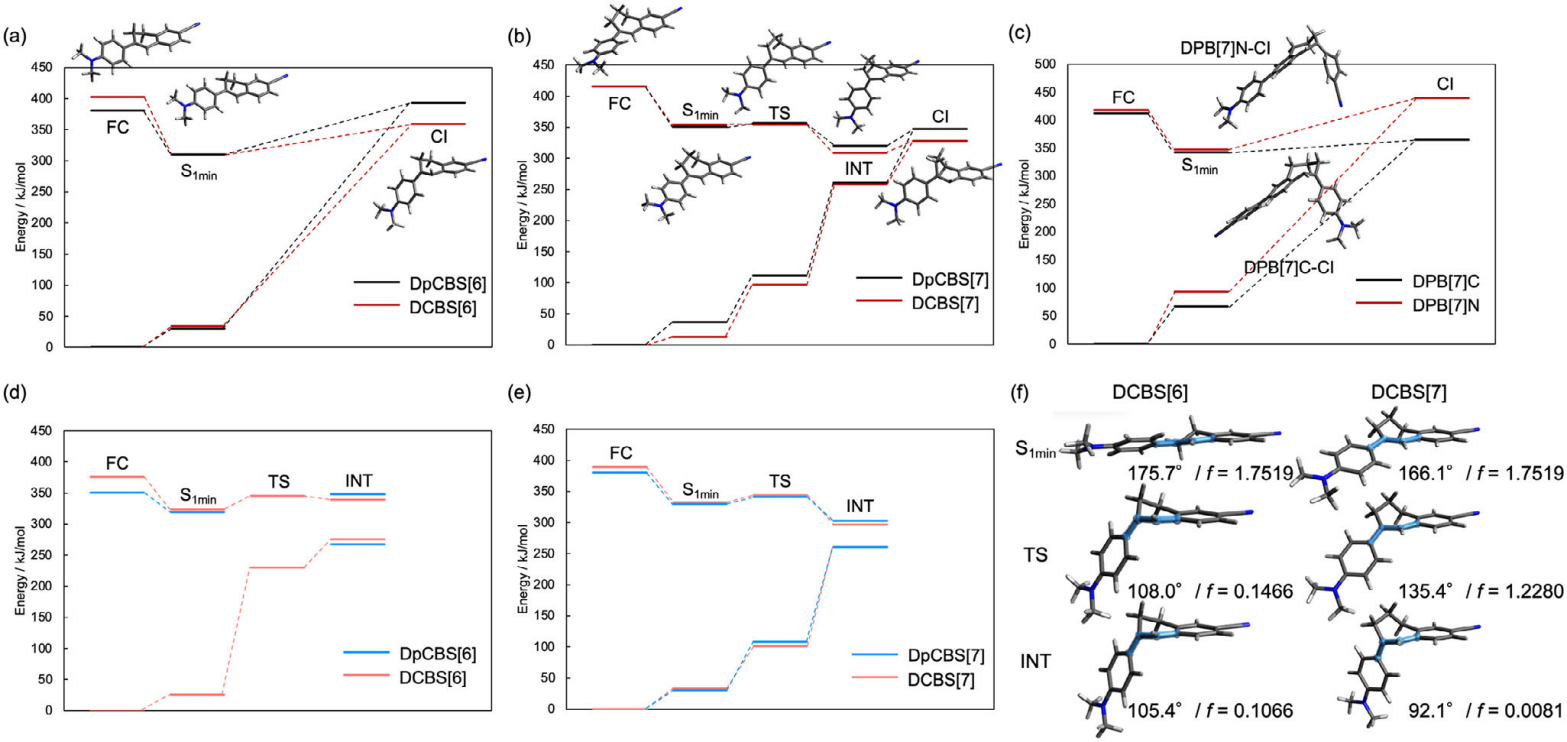
Energy diagrams and estimated structures of (a) DCBS[6] and DpCBS[6], (b) DCBS[7], DpCBS[7], and (c) DPB[7]C and DPB[7]N in the gas phase. All calculations were performed at the MRSF‐TDDFT BHHLYP/6‐31G(d) level. Energy diagrams of (d) DCBS[6] and DpCBS[6], (e) DCBS[7] and DpCBS[7] by using PCM‐MRSF‐TDDFT BHHLYP/6‐31G(d) in THF. (f) Optimized structures (S_1min_, TS, and INT) of DCBS[6] and DCBS[7] obtained at the PCM‐MRSF‐TDDFT BHHLYP/6‐31G(d) level in THF. The dihedral angles highlighted in cyan and the corresponding oscillator strengths (f) for each structure are indicated.

Here, we extend the discussion from the energetic accessibility of the CI to the overall post‐relaxation PES profiles connecting S_1min_ and the CI region. In the preceding comparative computational analyses, substituent effects were assessed primarily in terms of intrinsic CI accessibility using Δ*E* (FC–CI) and the CI energy as descriptors. Because nonradiative decay proceeds from the relaxed S_1min_ rather than directly from the FC region, we next tested whether compounds with low‐lying CIs also permit energetically accessible pathways from S_1min_. Accordingly, energy diagrams were constructed for representative donor–acceptor derivatives, including the FC, S1min, the transition state (TS) connecting S_1min_ to an INT‐like structure near the CI, and the MECI (Figure 4 and Table 1). INT‐like geometries were located for DpCBS[6], DCBS[6], DPB[7]C, and DPB[7]N. TS candidates connecting S_1min_ and INT were generated by linear interpolation in internal coordinates (LIIC), but subsequent eigenvector‐following optimizations did not converge to genuine TS structures. We therefore focus below on a comparative analysis of the relative energies of S_1min_ and the MECI to elucidate how substituent patterns modulate access to the CI region after structural relaxation.

**TABLE 1:** The Franck‐Condon state (FC), the local minimum near the FC (S_1min_), the transition state (TS) connecting S_1min_, and an intermediate near the CI (INT) and the minimum energy conical intersection (MECI), Δ*E*_TS‐S1min_ and Δ*E*_MECI‐FC_ normalized based on the ground state energy. (All energies in kJ mol^−1^).

	State	FC	S_1min_	TS^a^	INT	MECI	Δ*E*_TS‐S1min_	Δ*E*_MECI‐FC_
DCBS[7]	Gas	415	350	354	309	329	4.0	−86
	THF	389	332	344	297	n.a.	12	—
DpCBS[7]	Gas	415	351	357	320	347	6.0	−68
	THF	380	330	342	303	n.a.	12	—
DCBS[6]	Gas	403	339	—^b^	348	359	—	−44
	THF	376	324	340	339	n.a.	16	—
DpCBS[6]	Gas	381	339	—^b^	362	393	—	+12
	THF	351	319	—^b^	348	n.a.	—	—
DpCS	Gas	374	343	356	308	323, 378	13	−51, +4.0
DPB[7]N	Gas	418	347	—	—	440	—	+22
DPB[7]C	Gas	412	343	—	—	365	—	−4

^a^ Initial guess of TS structures connecting S_1min_ and CI was generated from linear interpolation of internal coordinates (LIIC). (Figure S5) Subsequently, geometry optimization was carried out to determine the true TS. The optimized TS was confirmed by vibrational analysis to exhibit a single imaginary frequency, and its connectivity to the S_1min_ and INT structures was examined by following the eigenvector of the imaginary frequency, as described in Section S3 and shown in Figure S6 of the Supporting Information.

^b^ TS does not converge during the calculations.

For DCBS[6] (Figure 4a), the excited state relaxes from FC to S_1min_, which is located 64 kJmol^−1^ lower in energy and characterized by a large oscillator strength (*f* = 1.6810), followed by nonradiative decay through a MECI situated 20 kJmol^−1^ above S_1min_. In comparison, for DpCBS[6], the MECI lies 54 kJmol^−1^ higher than S_1min_, DCBS[6] thus exhibits substantially enhanced CI accessibility. For DCBS[7] (Figure 4b), relaxation from FC yields an S_1min_ that is 64 kJmol^−1^ lower in energy (*f* = 1.7381). From there, the system crosses a small barrier of 4.0 kJmol^−1^ to reach an INT‐like geometry, which is essentially a low‐oscillator‐strength state (*f* ≈ 0.01), and then reaches a MECI 25 kJmol^−1^ below S_1min_. For comparison, the known AIE‐active DpCBS[7] features a MECI of only 4.0 kJmol^−1^ below S_1min_. The substantial lowering of the MECI energy in DCBS[7] therefore strongly suggests efficient nonradiative decay underlying its AIE behavior. Finally, for DPB[7]C and DPB[7]N (Figure 4c), distinct differences are observed. Both systems relax to S_1min_ located about 70 kJmol^−1^ below the FC (*f* = 1.6810), but DPB[7]N exhibits a MECI lying about 93 kJmol^−1^ above S_1min_, comparable to the high‐energy CI of DpCBS[6]. In contrast, DPB[7]C possesses a MECI of only 22 kJmol^−1^ above S_1min_, representing a marked improvement in CI accessibility. These energetic differences closely mirror the contrasting photophysical behaviors of the two compounds.

Up to this point, analysis of the excited‐state PES involving CIs has primarily been conducted under gas‐phase conditions. In general, continuum solvation models such as PCM and its variants (e.g., SMD) describe solvation effects based on the electronic density associated with a specific electronic state. However, in the vicinity of a CI, two electronic states become nearly degenerate and strongly mixed. Under such conditions, the ambiguous definition of the solvation Hamiltonian often results in root flipping during the solution of the secular equation and leads to poor convergence behavior [51]. To address these limitations, several advanced solvation approaches have been developed, including state‐averaged solvent models [52], self‐consistent solvent models [53], and dynamically adaptive solvent models [51], which have been applied to investigate solvent effects on CIs. However, considering their substantial computational cost, such treatments are beyond the scope of the present study. For regions where the electronic state can be clearly defined, such as the FC, S_1min_, TS, and INT, the application of PCM is more appropriate. We therefore performed PCM‐MRSF‐TDDFT calculations for these stationary points and constructed the corresponding energy diagrams (Figure 4d,e), with the energies summarized in Table 1. Overall, the relative energies of the key stationary points are largely preserved between the gas phase and THF, suggesting that the substituent‐dependent trends in CI accessibility are largely preserved under solvent conditions.

Notably, in contrast to the gas‐phase calculations, a TS connecting S_1min_ and INT‐like geometry could be identified for DCBS[6] in THF, with a barrier of 16 kJmol^−1^ relative to S_1min_. Analysis of the electronic character and geometry (Figure 4f) indicates that this structure is closer to the INT‐like geometry, suggesting a late, product‐like TS. In contrast, for DCBS[7], the corresponding structure is located between S_1min_ and INT, suggesting that the transition state appears earlier along the reaction coordinate, consistent with a more gradual excited‐state evolution. This distinction parallels the behavior observed for the unsubstituted BST[6] and BST[7] systems (Figure S3) and suggests that the PES shape—particularly the connectivity between the S_1_ region and the CI—is likely influenced by the bridge size. Although TS structures are expected to exist, our attempts to locate them were not successful for all systems, likely due to convergence difficulties. Nevertheless, the overall energy profiles and the consistent trends across related molecules support a deactivation process in which the system evolves from S_1min_ toward the CI region via INT‐like geometries. Taken together, these results indicate that, beyond the energetic position of the MECI, the overall PES profile plays an important role in modulating nonradiative decay behavior.

### 2.5 Experimental Correlation Between CI Accessibility and Photophysical Behavior

To examine the relationship between computed CI accessibility and experimentally observed photophysical behavior, we synthesized DCBS[6], DCBS[7], DPB[7]C, and DPB[7]N, along with CpBS[6] (bb) and DBS[6] (aa), and systematically examined their photophysical properties (Figures S7‐S15 and Tables S1–S6). CpBS[6] was selected as a reference compound exhibiting low CI accessibility (Figure 2a,b), whereas DBS[6] emerged as a promising candidate. Synthetic procedures are provided in Section S7 of the Supporting Information. Key spectroscopic parameters—absorption maxima (*λ*_abs_), emission maxima (*λ*_fl_), fluorescence quantum yields (*Φ*_fl_) in toluene, and amorphous solid‐state *Φ*_fl_—are summarized in Table 2. In agreement with the computed CI‐accessibility trends, DCBS[7], predicted to exhibit high CI accessibility, is nearly nonemissive in toluene (*Φ*_fl_ = 0.01) but becomes moderately emissive in the solid state (*Φ*_fl,solid_ = 0.36). A similar trend is observed for the known AIE reference DpCBS[7] (*Φ*_fl_ = 0.01; *Φ*_fl,solid_ = 0.70). In contrast, DCBS[6], predicted to exhibit only moderately enhanced CI accessibility relative to DpCBS[6], shows weak emission in toluene (*Φ*_fl_ = 0.06) and limited solid‐state enhancement (*Φ*_fl,solid_ = 0.18), consistent with its intermediate position in the calculated energetic trends.

**TABLE 2:** Spectroscopic properties (absorption maxima *λ*_abs_, fluorescence maxima *λ*_fl_, absolute quantum yields *Φ*_fl_) of DCBS[6], DCBS[7], DpCBS[6], DpCBS[7], DPB[7]N and DPB[7]C in toluene or THF and the solid‐state.

	*λ*_abs_ (nm)	*λ*_fl_ (nm)	*Φ*_fl_	*Φ*_fl, solid_
DCBS[6]	382^a^	473^a^	0.06^a^	0.18
DCBS[7]	363^a^	473^a^	0.01^a^	0.36
DpCBS[6]	402^a^	471^a^	0.76^a^	0.12
DpCBS[7]	379^a^	469^a^	0.01^a^	0.70
DPB[7]N	361^a^	479^a^	0.88^a^	0.88
DPB[7]C	363^a^	484^a^	0.09^a^	0.95
CpBS[6] (cc)	331^b^	401^b^	0.88^b^	0.21
DBS[6] (bb)	344^b^	431^b^	0.06^b^	0.38

^a^ Measured in toluene.

^b^ Measured in THF.

For the π‐extended derivatives, the computations suggested that the CI for DPB[7]C is more accessible than that for DPB[7]N. In agreement with the calculations, DPB[7]C displays low solution fluorescence (*Φ*_fl_ = 0.09) but strongly enhanced solid‐state emission (*Φ*_fl,solid_ = 0.95), characteristic of AIEE behavior. By contrast, DPB[7]N remains highly emissive in both solution and the solid state (*Φ*_fl,solid_ = 0.88), indicative of suppressed nonradiative decay due to limited CI accessibility. Further support comes from control compounds lacking a donor–acceptor pair. CpBS[6] exhibits high solution fluorescence (*Φ*_fl_ = 0.88) but markedly reduced solid‐state efficiency (*Φ*_fl,solid_ = 0.21), whereas DBS[6] shows weak solution emission (*Φ*_fl_ = 0.06) and pronounced solid‐state enhancement (*Φ*_fl,solid_ = 0.38), characteristic of AIE behavior.

Overall, the experimentally observed fluorescence behavior closely follows the computed substituent‐dependent CI trends, supporting CI accessibility as a useful descriptor for understanding AIE and AIEE behavior within this bridged stilbene series. Notably, the donor/acceptor‐inverted pair DCBS[6]/DpCBS[6] displays an opposite solution/solid behavior, directly supporting that substituent orientation can switch CI accessibility even within the more rigid BST[6] scaffold.

### 2.6 Solvent Effects on Photophysical Properties

**FIGURE 5:**
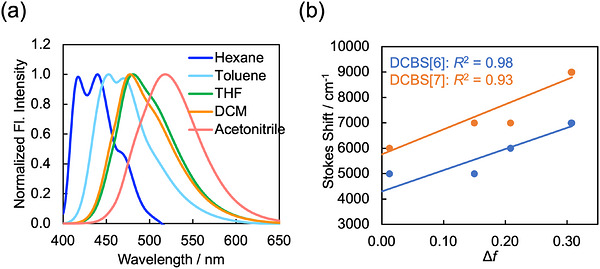
(a) Fluorescence spectra of DCBS[6] recorded in solvents covering a wide range of polarities. (b) Lippert–Mataga plots of DCBS[6] and DCBS[7].

Here, we focus on DCBS[6] and DCBS[7] to elucidate how the substituent arrangement governs excited‐state behavior in solution. In solvents of varying polarity (Tables S1 and S2), both compounds exhibit solvatochromic emission, comparable to their reference analogues DpCBS[6] and DpCBS[7] (Figure 5b). Lippert–Mataga [54] analyses show linear correlations between Stokes shift and solvent polarity (R^2^ = 0.98 for DCBS[6] and 0.93 for DCBS[7]; Figure 5b), indicating significant charge‐transfer character in the emissive states. Despite similar solvatochromism, their fluorescence efficiencies differ markedly: DCBS[7] is essentially nonemissive across a wide polarity range (*Φ*_fl_ ≤ 0.02), whereas DCBS[6] shows weak emission only in low‐ to medium‐polarity solvents (*Φ*_fl_ ≤ 0.10). These results indicate that, while the emissive state nature is similar, nonradiative deactivation efficiency is strongly modulated by ring size and substituent arrangement.

### 2.7 Solid‐State Emission Behavior and Aggregate Structures

**FIGURE 6:**
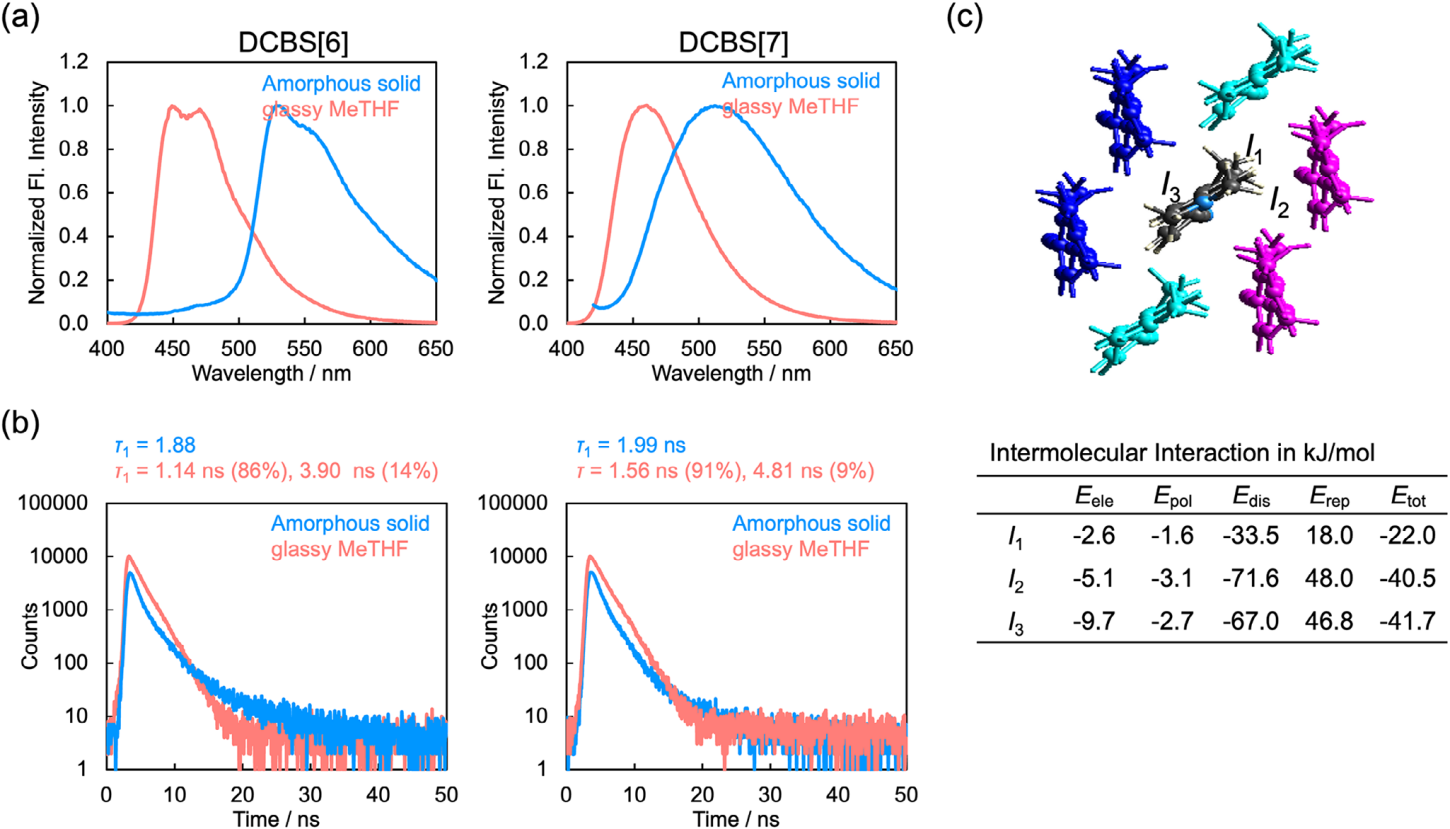
(a) Fluorescence spectra of DCBS[6] and DCBS[7] in the amorphous solid state and in a glassy MeTHF matrix at 80 K. (b) Fluorescence decay profiles in the amorphous solid state and in a glassy MeTHF matrix, recorded with excitation at 402.5 nm and detection at the emission wavelengths (*λ*_fl_). The decay curves were analyzed by tail fitting. (c) Single crystal structures of DCBS[6] and the corresponding intermolecular interaction energies (kJ mol^−1^) evaluated by CrystalExplorer at the B3LYP/6‐31+G(d,p) level. *E*_tot_ denotes the total intermolecular interaction energy.

To elucidate the emissive species and nonradiative decay processes in the solid state, fluorescence spectra and lifetimes of DCBS[6] and DCBS[7] were measured in the amorphous solid state and in a glassy 2‐methyltetrahydrofuran (MeTHF) matrix at 80 K (Figure 6a,b and Figure S16). The glassy MeTHF matrix at 80 K serves as a reference for monomeric emission, as intermolecular interactions and CI accessibility are largely suppressed under these conditions. While single crystals of DCBS[7] could not be obtained, the single‐crystal x‐ray structure of DCBS[6] was successfully determined (Figure 6c).

In the amorphous solid state, the emission spectra of DCBS[6] and DCBS[7] are red‐shifted by approximately 80 and 50 nm, respectively, relative to those in the glassy MeTHF matrix at 80 K (Figure 6a). This pronounced red shift indicates that the solid‐state emission cannot be attributed solely to monomeric species but instead likely originates from electronically coupled aggregate states. For DCBS[7], the fluorescence lifetime in the glassy MeTHF matrix at 80 K is 1.99 ns, whereas in the solid state at 300 K a shorter lifetime of 1.56 ns is observed together with a minor long‐lived component of 4.24 ns (9%). In the glassy matrix, suppression of intermolecular coupling and restricted CI accessibility result in highly efficient emission (*Φ*_fl_ ≈ 1). By contrast, the reduced solid‐state quantum yields of DCBS[7] (*Φ*_fl_ = 0.36) and DCBS[6] (*Φ*_fl_ = 0.18) indicate enhanced nonradiative decay in the solid state. The distinct *Φ*_fl_ values further suggest that the solid‐state nonradiative decay is strongly influenced by differences in intermolecular coupling arising from molecular packing.

Single‐crystal analysis of DCBS[6] reveals a planar molecular structure with a herringbone‐type packing motif (Figure 6c and Figure S28 and Table S13). Energy decomposition analysis indicates that the stabilization is dominated by dispersion interactions (*E*_dis_) balanced by Pauli repulsion (*E*_rep_), thereby maintaining a finite intermolecular separation [55]. Nevertheless, the planar geometry and herringbone packing motif promote intermolecular electronic coupling, thereby enhancing nonradiative decay and reducing *Φ*_fl_. In contrast, the seven‐membered bridge reduces molecular planarity and intermolecular coupling, consistent with the higher solid‐state *Φ*_fl_ of DCBS[7]. Such behavior is characteristic of seven‐membered bridged stilbene derivatives, in which the inherent conformational distortion introduced by the larger ring reduces intermolecular electronic coupling and favors radiative decay in the solid state [56, 57].

**FIGURE 7:**
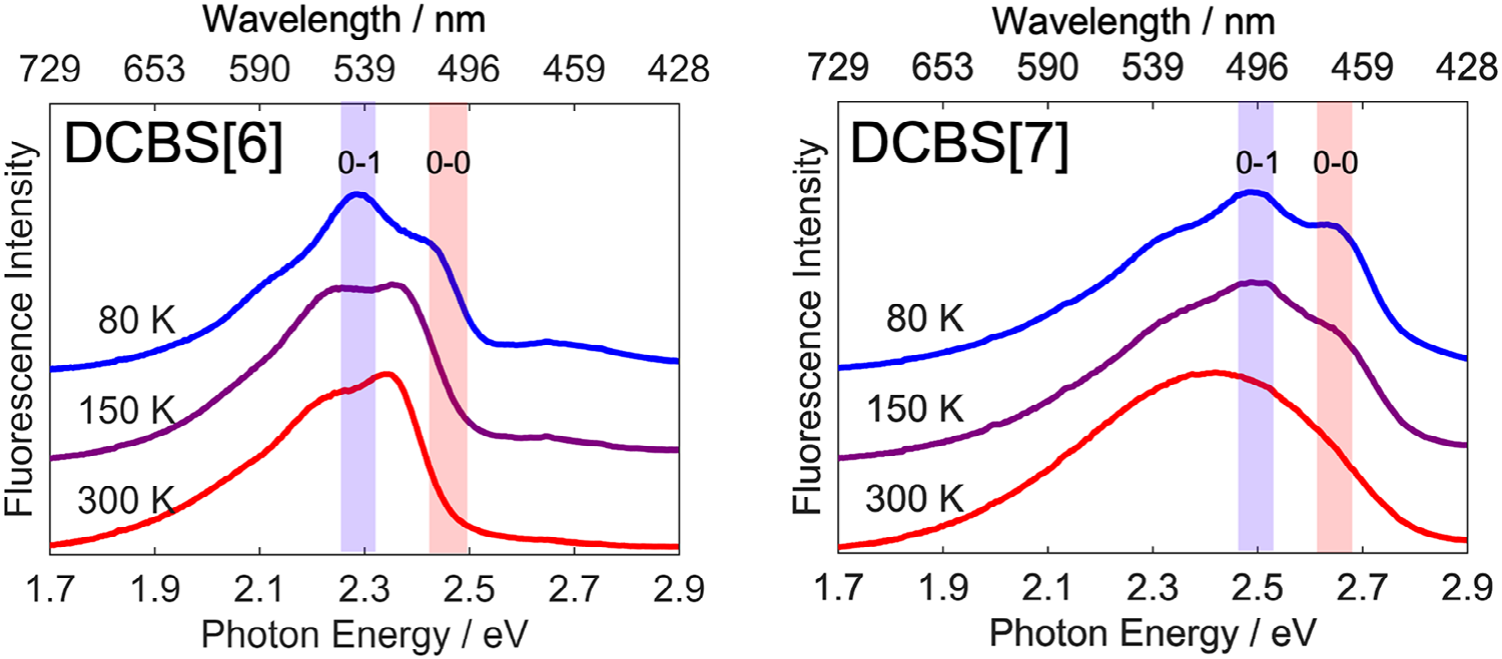
Temperature‐dependent fluorescence spectra of DCBS[6] and DCBS[7] in the amorphous solid state. Purple and red shaded bars indicate the vibronic bands originating from the C═C stretching mode, corresponding to the 0–1 and 0–0 transitions, respectively. Spectra were recorded at 80, 150, and 300 K.

To further probe emissive species in the amorphous solid state, temperature‐dependent fluorescence spectra and wavelength‐resolved fluorescence lifetime measurements were performed (Figure 7 and Figure S17–S26 and Tables S7–S12). At 80 and 150 K, both compounds exhibit vibronic structures corresponding to the 0–0 and 0–1 transitions with an energy spacing of ∼0.2 eV, consistent with the C_et_–C_et_ stretching mode (1600–1650 cm^−1^). For DCBS[7], these vibronic features gradually diminish with increasing temperature and evolve into a broad emission band at 300 K, indicating that the low‐temperature vibronic emission originates primarily from monomeric species [58]. As discussed above, this behavior is consistent with the coexistence of monomeric and excimer‐like emissive states in the amorphous solid state. In contrast, the vibronic structure observed for DCBS[6] persists across the entire temperature range and exhibits a slight red shift relative to that in the glassy MeTHF matrix. Considering that the overall emission of DCBS[6] is significantly red‐shifted relative to the glassy‐matrix monomer reference (Figure 6), the vibronic bands near 510 and 540 nm are unlikely to arise from isolated monomer emission. Instead, these features are more plausibly attributed to emissive aggregate states. Based on the single‐crystal structure (Figure 6c) and the persistent emission band near 540 nm (Figure 7), this emissive state is most reasonably assigned to an aggregate packing [59, 60, 61, 62, 63]. Notably, a very weak emission band near 460 nm remains detectable at 80 K for DCBS[6], which may correspond to a residual monomeric 0–0 transition. The low intensity of this feature suggests that monomer emission contributes only minimally to the overall solid‐state emission of DCBS[6].

### 2.8 fs‐Transient Absorption Spectroscopy (fs‐TAS)

**FIGURE 8:**
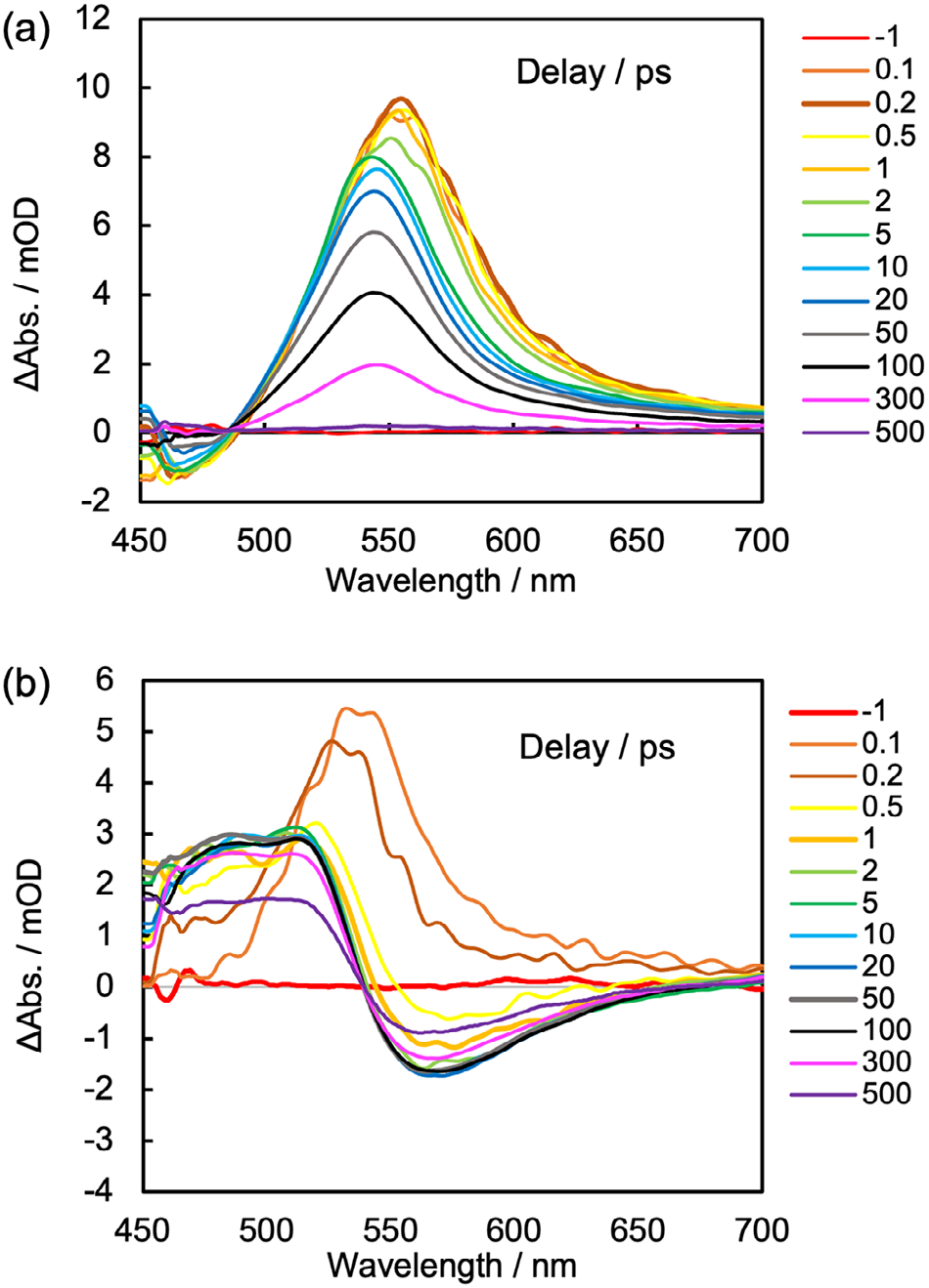
Transient absorption spectra of DCBS[6] recorded at various delay times after excitation at 400 nm at room temperature in: (a) toluene and (b) acetonitrile.

Steady‐state photophysical measurements revealed that DCBS[6] exhibits markedly different photophysical behavior from the previously reported DpCBS[6]. To elucidate the origin of this difference, we investigated DCBS[6], DCBS[7], DpCBS[6], and DpCBS[7] in detail using femtosecond transient absorption (fs‐TA) spectroscopy to estimate their excited‐state lifetimes (τ). Figure 8 shows the TA spectra of DCBS[6] in toluene and acetonitrile at various delay times; corresponding data for DCBS[7] are provided in Figure S27. For DCBS[6], excited‐state absorption (ESA) bands appear at approximately 550 nm in toluene and 500 nm in acetonitrile, accompanied by bleaching signals near 460 and 570 nm, which are assigned to ground‐state bleaching and stimulated emission, respectively. The ESA band exhibits a gradual blueshift after photoexcitation, which is consistent with vibrational relaxation and structural relaxation within the S_1_ state from the FC region [30, 64]. No additional distinct transient features are resolved within the present spectral window.

Here, the fs‐TA data were analyzed by global fitting using Glotaran with a two‐component kinetic model (Table 3). This model was adopted because the spectral evolution is adequately described by an initial spectral relaxation followed by a single exponential‐like decay, without the appearance of a clearly separable additional transient component. The first component (τ_1_) was assigned to vibrational and structural relaxation on the sub‐picosecond to picosecond timescale, consistent with the observed ESA blueshift. The slower component (τ_2_) was assigned to internal conversion from S_1_ to S_0_. Because τ_2_ predominantly reflects S_1_→S_0_ internal conversion dynamics, we focus on this component in the following discussion. For DCBS[6], τ_2_ values of 142 ps in toluene and 777 ps in acetonitrile were obtained, which are significantly shorter than those of DpCBS[6] (1.77 and 2.22 ns, respectively). Similarly, DCBS[7] shows shorter τ_2_ values (21.1 ps in toluene and 49.7 ps in acetonitrile) than its substituent‐inverted analogue DpCBS[7] (28.9 and 76.1 ps, respectively). These accelerated S_1_→S_0_ internal conversion dynamics in DCBS[6] and DCBS[7], relative to DpCBS[6] and DpCBS[7], are fully consistent with the enhanced CI accessibility suggested by the quantum‐chemical PES analyses.

**TABLE 3:** The estimated lifetime τ_1_ and τ_2_ of DCBS[6], DpCBS[6], DCBS[7], and DpCBS[7] in toluene and acetonitrile by TAS using global analysis (Glotaran).

		DCBS[6]	DpCBS[6]	DCBS[7]	DpCBS[7]
Toluene	τ_1_ (ps)	3.1	2.9	1.8	3.7
	τ_2_ (ps)	142	1773	21.1	28.9
Acetonitrile	τ_1_ (ps)	0.57	0.52	3.2	0.45
	τ_2_ (ps)	777	2219	49.7	76.1

Although the calculations suggest the presence of an intermediate INT‐like geometry near the CI for some compounds, its absence as a distinct fs‐TA feature does not preclude its involvement in the relaxation process. Rather, the INT is calculated to possess very low oscillator strength and is therefore unlikely to produce a distinct spectroscopic signature. In addition, such an intermediate is expected to be very short‐lived and may not accumulate sufficiently to be resolved experimentally. Furthermore, the characteristic signal of a stilbene‐like intermediate may lie outside the present detection window [29] or be spectrally overlapped with the broader ESA and bleaching features. Thus, the fs‐TA results are consistent with the calculated relaxation pathway, even though the INT is not directly observed as an isolated transient species.

### 2.9 Topology of Conical Intersection

In this section, we will discuss the rate of nonadiabatic transitions around the CI. Because nonadiabatic transitions occur most efficiently near the CI [65, 66, 67, 68, 69, 70], MECIs are often regarded as the most important points acting as funnels that connect the excited‐state and ground‐state PESs [71, 72, 73, 74, 75, 76, 77], although nonradiative decay can occur at other points as well. Therefore, understanding the energy landscape around the MECI is essential for estimating the rate of nonadiabatic transitions. Near the CI, PESs consist of an (*f*‐2)‐dimensional intersection space in which the energies of the two states remain degenerate and a two‐dimensional branching space (where *f* = 3*N*‐6 is the number of nuclear degrees of freedom). The branching space is spanned by two vectors:

(1) $${\vec{g}}_{\mathrm{IJ}}=\frac{\partial }{\partial \vec{R}}\left(E_{I}-E_{J}\right)$$

(2) $${\vec{h}}_{\mathrm{IJ}}=\left(E_{I}-E_{J}\right)\;\langle \psi_{I}\left|\frac{\partial }{\partial \vec{R}}\right|\psi_{J}\rangle$$

where, *E*_I_ and *E*_J_ denote the energies of states I and J, respectively, and $\vec{R}$ represents the nuclear coordinates. ψ_*I*_ and ψ_*J*_ are the wavefunctions of states I and J, respectively. Displacement along these two vectors lifts the degeneracy between the two states, resulting in the characteristic double‐cone topology of the PESs. The local topology around a CI, first classified by Ruedenberg [78], is known to influence the efficiency of nonradiative decay around the CI [79, 80, 81, 82]. To analyze the CI topology, two‐dimensional PESs (2D PESs) were constructed using the ${\vec{g}}_{IJ}$ and ${\vec{y}}_{IJ}$ in the vicinity of the MECI [65, 66, 67, 68, 69, 70].

**FIGURE 9:**
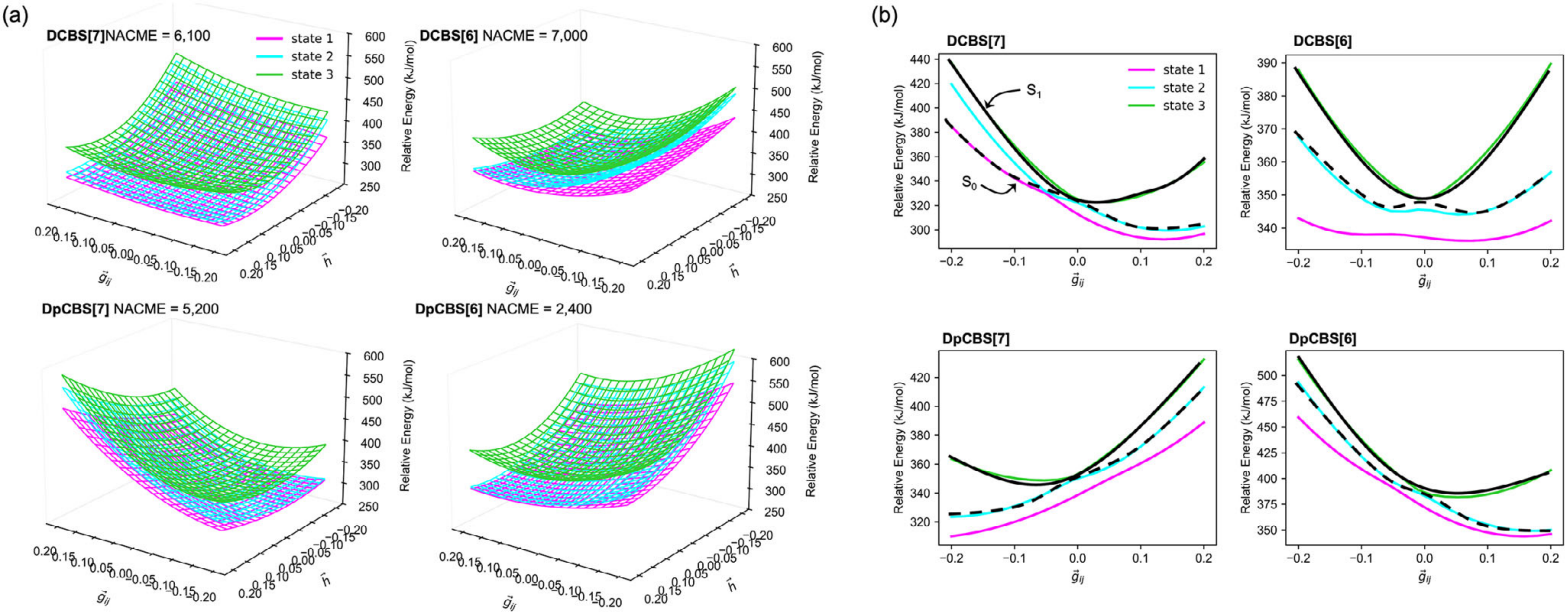
(a) Two‐dimensional potential energy surfaces (2D‐PESs, in kJ mol^−1^) of S_0_ and S_1_ constructed along the branching‐plane coordinates defined by the gradient difference vector (GDV, ${\vec{g}}_{IJ}$) and the derivative coupling vector (DCV, ${\vec{h}}_{IJ}$). The corresponding NACME values are indicated in $\sqrt{\mathrm{amu}}\cdot \mathrm{Boh}r^{-1}$. (b) One‐dimensional PES along GDV, ${\vec{g}}_{IJ}$ centered at the MECI (${\vec{h}}_{IJ}$ = 0). The assigned S_1_ and S_0_ surfaces are shown by the black solid and black dashed lines, respectively.

In Sections 2.1–2.4, MECIs were located using MRSF‐TDDFT with the branching plane updating method [83]. It should be noted that although MRSF‐TDDFT effectively suppresses spin contamination, analytical derivative coupling vectors (DCVs, ${\vec{h}}_{IJ}$) are not implemented in GAMESS. Instead, we employed branching plane updating method, which calculates the ${\vec{g}}_{IJ}$ and an approximate derivative coupling vector (${\vec{y}}_{IJ}$), orthogonal to ${\vec{g}}_{IJ}$, that converges to ${\vec{h}}_{IJ}$ during geometry optimization. The resulting 2D‐PES obtained by this method is shown in Figure S32. The vectors ${\vec{g}}_{IJ}$ and ${\vec{y}}_{IJ}$ are illustrated in Figures S30 and S31. In this section, we focus on 2D‐PES constructed with analytical DCV obtained with spin‐flip TDDFT (SF‐TDDFT‐BHHLYP/6‐31G(d)) as implemented in Q‐Chem [84]. The MECIs were optimized starting from the MRSF‐TDDFT geometries, yielding essentially identical structures. To confirm that the electronic states obtained at the MECIs by the two methods correspond to the same states, we compared the branching plane around the MECI. The PES obtained by MRSF‐TDDFT with the approximate ${\vec{h}}_{IJ}$ is shown in Figure S32, whereas the PES obtained by SF‐TDDFT with the analytical DCV is presented in Figure 9. Note that in a CI, the two degenerate states can be mixed arbitrarily, and therefore ${\vec{g}}_{IJ}$ and ${\vec{h}}_{IJ}$ cannot be uniquely defined because a rotational degree of freedom always remains within the branching plane. Nevertheless, apart from this arbitrariness of the basis, the resulting PESs are very similar. This agreement indicates that both approaches describe essentially the same electronic structure around the MECI.

In the following discussion, we provide an overview of the topology of the DCBS[*m*] and DpCBS[*m*] and discuss the possibility of nonadiabatic transitions around the CIs. It should be noted that the PES presented here were derived from gas‐phase calculations and may not be directly compared with actual PES under polar solvents. Figure 9 displays the PESs of the three lowest states, which correspond to mixtures of S_0_, S_1_, and T_1_ characters. Although spin contamination was small at the optimized MECI, we found that some states were highly spin‐contaminated at some points. A rigorous assignment of each surface throughout the branching plane lies beyond the scope of the present work. Comparison with Figure S32, in which the spin multiplicities are correctly described, shows that the overall shapes of the surfaces are qualitatively consistent. Therefore, we consider Figure 9a sufficiently reliable for discussing the CI topology.

Figure 9b shows cross sections of the PESs along the ${\vec{g}}_{IJ}$ direction. We assigned spin states by following the continuity of the electronic character of the states across the crossing region. The assigned S_1_ and S_0_ surfaces are indicated by the black solid and dashed lines, respectively. DCBS[6] exhibits a peaked CI topology in the vicinity of MECI, and DCBS[7] also shows a largely peaked topology, although with a slight tilt of the PES. In contrast, DpCBS[6] and DpCBS[7] exhibit sloped CI topologies. A peaked CI can be accessed downward from many directions and consequently promotes efficient internal conversion [72]. On the other hand, a sloped CI allows the possibility of returning to the excited‐state surface and thus reduces the probability of nonradiative decay [85, 86, 87, 88]. Because the ${\vec{h}}_{IJ}$ reflects interstate electronic coupling, these results indicate stronger electronic coupling along ${\vec{h}}_{IJ}$ at the CI for DCBS[6] than for DpCBS[6] (and similarly for DCBS[7] compared with DpCBS[7]). This distinction provides a clear rationale for the different fluorescence behaviors of DCBS[6] and DpCBS[6]. Consistent with this interpretation, the NACME of DCBS[6] reaches 7000 $\sqrt{\mathrm{amu}}\cdot \mathrm{Boh}r^{-1}$, nearly three times larger than that of DpCBS[6], highlighting a pronounced enhancement in internal conversion efficiency. Moreover, DCBS[7] shows a slightly larger NACME of 6100 $\sqrt{\mathrm{amu}}\cdot \mathrm{Boh}r^{-1}$ than DpCBS[7], further supporting the role of substituent inversion in stabilizing a peaked CI topology and promoting efficient nonradiative decay.

Next, we compared the CI topologies of the parent compounds BST[7] and BST[6] with those of push–pull‐type bridged stilbenes to assess the role of substituents. As shown in Figure S33, both BST[7] and BST[6] exhibit sloped‐type CIs. In contrast, the corresponding push–pull derivatives display peaked CI topologies. These results suggest that, in the gas phase, donor–acceptor substitution locally reshapes the PES topology around the CI, converting it from sloped to peaked in the systems examined here and thereby facilitating more efficient internal conversion.

## 3 Integrated Discussion and Limitations

**FIGURE 10:**
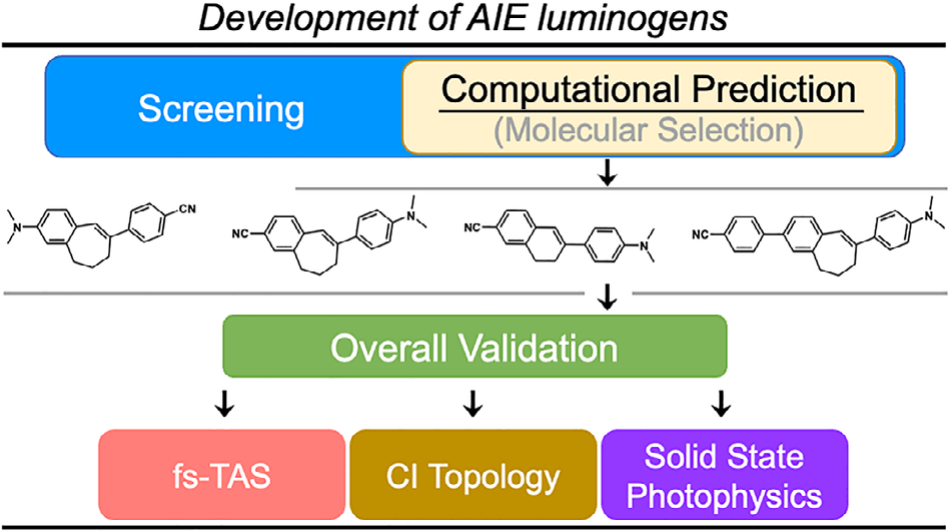
Schematic overview of the integrated structure–property framework for AIE luminogens. Comparative computational analyses are first employed to identify substituent‐dependent trends in CI accessibility, from which representative D–π–A derivatives are selected. These candidates are subsequently investigated through combined experimental and theoretical analyses, including fs‐transient absorption spectroscopy (fs‐TAS), conical intersection (CI) topology analysis, and solid‐state photophysical measurements, thereby establishing an integrated structure–property relationship.

Figure 10 summarizes the overall structure–property relationship and mechanistic framework developed in this study. Consistent with the strategy outlined at the beginning of the Results and Discussion, this study presents a structure–property framework that links molecular structure, CI accessibility, and photophysical behavior in bridged stilbene AIEgens (Figure 10). We first employed comparative computational analyses to explore how bridge size and substituent placement affect CI accessibility, using Δ*E* (FC–CI) and CI energy as practical descriptors. This analysis revealed a substituent‐dependent trend within this asymmetric molecular framework, in which donor–acceptor substitution tends to stabilize the CI relative to the FC region. On the basis of these insights, representative D–π–A derivatives were synthesized and investigated to determine whether the computed energetic trends are reflected experimentally. Their photophysical properties in solution and the solid state generally followed the computed CI‐accessibility trends, suggesting that substituent inversion provides a useful approach for modulating nonradiative decay and AIE behavior.

Beyond these initial comparative computational analyses and experimental investigations, subsequent analyses further clarify the excited‐state deactivation process. Energy diagrams describing access from S_1min_ toward the CI suggest that CI accessibility depends not only on the energetic position of the CI, but also on the shape of the PES along the relaxation coordinate, including the involvement of INT‐like configurations. Furthermore, CI topology analysis indicates that substituent‐dependent reshaping of the PES—from sloped to more peaked intersections—is associated with enhanced nonadiabatic coupling and more efficient internal conversion. In addition, consistent with the defining feature of AIE, solid‐state photophysical measurements reveal enhanced radiative decay and suppressed nonradiative decay upon aggregation. These observations are consistent with reduced access to low‐lying CI regions related to nonradiative decay, thereby supporting a structure–property relationship across different phases. Taken together, these results provide a coherent picture of AIEgen photophysics, in which comparative computational analyses reveal substituent‐dependent energetic trends and subsequent experimental and theoretical studies support a consistent mechanistic interpretation. Within the bridged stilbene systems examined here, this approach provides a practical and physically grounded strategy for connecting molecular design with excited‐state deactivation behavior.

Several limitations should be noted. CI‐related PES analyses were primarily conducted in the gas phase, as continuum solvation models can become ill‐defined in regions where multiple electronic states are strongly mixed. Although PCM calculations in THF preserve the overall energetic trends, solvent effects near the crossing region are expected to remain approximate. In addition, full mapping of the excited‐state pathways was not achieved for all systems, and TS structures could not always be reliably located. Finally, explicit nonadiabatic excited‐state dynamics simulations were not performed, and the proposed relaxation pathways are therefore inferred on the basis of static PES analysis, CI topology, and fs‐TA lifetimes.

## 4 Conclusion

In this work, we established computation‐guided structure–property relationships linking donor–acceptor asymmetry and CI accessibility in bridged stilbene AIE luminogens. Systematic evaluation based on Δ*E* (FC–CI) and CI energies revealed clear substituent‐dependent trends, which guided the synthesis and photophysical investigation of representative push–pull alkylene‐bridged stilbenes, including DCBS[6], DCBS[7], DPB[7]C, and DPB[7]N. Their fluorescence quantum yields and excited‐state lifetimes generally followed the computed energetic trends, highlighting the important role of asymmetric donor–acceptor placement in modulating excited‐state deactivation behavior.

Combined computational, spectroscopic, and branching‐plane PES analyses suggest that steric asymmetry and substituent‐induced polarization around the C_et_─C_et_ bond cooperatively stabilize low‐lying CI geometries and facilitate access to nonradiative decay pathways. In particular, substituent inversion reshapes the local PES topology from sloped to more peaked intersections and enhances nonadiabatic coupling, as reflected by the substantially larger NACME values observed for DCBS[6] relative to DpCBS[6]. These results collectively support a mechanistic picture in which bridge‐induced geometric constraints and asymmetric electronic effects jointly govern CI accessibility and internal conversion efficiency.

More broadly, the present findings suggest that donor–acceptor asymmetry can serve as an effective strategy for tuning excited‐state potential‐energy landscapes and nonradiative decay behavior in π‐conjugated systems. Although the generality of this approach beyond the present molecular framework remains to be established, the ability to modulate CI accessibility through substituent‐level asymmetry provides a useful basis for designing excited‐state dynamics in functional luminogens.

## Author Contributions

**Takuya Tanaka**: conceptualization, investigation, writing – review and editing, writing – original draft, methodology. **Hirosato Koyanagi**: investigation. **Kazunobu Igawa**: investigation. **Ben Zhong Tang**: supervision, writing – review and editing. **Riki Iwai**: investigation. **Gen‐ichi Konishi**: conceptualization, methodology, investigation, writing – review and editing, writing – original draft. **Satoshi Suzuki**: methodology, writing – review and editing. **Kiyoshi Miyata**: investigation, writing – review and editing. **Ken Onda**: methodology, investigation.

## Funding

This project was supported in part by MEXT/JSPS KAKENHI grants 23H02036, 25K22304 (G.K.), 23H04631, 23K20039, 23K26670, 23H03833, 23H01977, 24K01471, 24K01515, 25H01678 (K.M.), 23K01977, 23K20039, 25K01678 (K.O.), 24K08341 (S.S.), JST SPRING, Grant Number JPMJSP2180 (T.T.), and the Sasakawa Scientific Research Grant (T.T.).

## Conflicts of Interest

The authors declare no conflicts of interest.

## Supporting information




**Supporting File 1**: advs76058‐sup‐0001‐SuppMat.pdf.

**Supporting File 2**: advs76058‐sup‐0002‐Data.zip.

## Data Availability

The data that support the findings of this study are available in the supplementary material of this article.
