# Supplementary material for: Computation‐Guided Control of Excited‐State Deactivation through Modulation of Conical‐Intersection Accessibility by Donor–Acceptor Asymmetry in Bridged Stilbene AIE Luminogens
Source: Adv Sci (Weinh). 2026 Jun 18:e76058. Online ahead of print. doi: 10.1002/advs.76058 (PMC13336361; doi:10.1002/advs.76058)
Supplement: Supplementary file 1 — Supporting File 1: advs76058‐sup‐0001‐SuppMat.pdf. [file ADVS-9999-e76058-s002.pdf]

Supporting Information for

Computation-Driven Asymmetric Donor–Acceptor Control of Conical Intersection Accessibility Enables Rational Design of AIE Luminogens

Table of contents

S1. General procedure
S2. Materials
S3. Calculation
S4. Photophysical properties
4-1. Optical properties. (Absorption, fluorescence, excitation and fluorescence lifetime)
4-2. Femtosecond transient absorption spectroscopy.
S5. Crystallographic information
S6. 2D Potential Energy Surface (2D-PES)

S7. Synthesis and characterization
7-1. Synthesis of 8-(4-(dimethylamino)phenyl)-6, 7-dihydro-5*H*-benzo[7]annulene-3-carbonitrile (**DCBS[7]**)
7-2. Synthesis of 6-(4-(dimethylamino)phenyl)-7, 8-dihydronaphthalene-2-carbonitrile (**DCBS[6]**)
7-3. Synthesis of 4-(8-(4-(dimethylamino)phenyl)-6,7-dihydro-5*H*-benzo[7]annulen-3-yl)benzonitrile (**DPB[7]C**) and 4-(3-(4-(dimethylamino)phenyl)-6,7-dihydro-5*H*-benzo[7]annulen-8-yl)benzonitrile (**DPB[7]N**)
7-4. Synthesis of 4-(3,4-dihydronaphthalen-2-yl)benzonitrile (**CpBS[6]**) and 4-(3,4-dihydronaphthalen-2-yl)-*N*,*N*-dimethylaniline (**DBS[6]**)

S8. Reference

S1. General procedure

Characterization

^{1}H NMR and ^{13}C NMR spectra were recorded on 500 MHz (125 MHz for ^{13}C NMR) BRUKER spectrometer or 100 MHz JEOL 400 spectrometers for $CDCl_3$ solutions using tetramethyl silane (TMS) as the internal standard. The date for ^{1}H-NMR was reported as follows: chemical shift (δ ppm), multiplicity (s = singlet, d = doublet, t = triplet, q = quartet, quin = quintet, sex = sextet m = multiplet), integration, coupling constant (Hz). ^{13}C NMR spectra were reported as chemical shifts in ppm and multiplicity where appropriate. Column chromatography was carried out with Kanto Chemical silica gel 60N (40–50 mesh). TLC was carried out with Merck Silica Gel 60 F254 (0.2 mm) plate. Fourier transform infrared (FT-IR) spectra were recorded on a JASCO FT-IR 469 plus spectrometer with a universal Zn-Se ATR (attenuated total reflection) accessory in the 600-4000 cm^{-1}. Melting points (m.p.) were recorded on a Yanaco micro melting point apparatus. The mass spectrometry (MS) was performed on JEOL JMS700 mass spectrometer. JMS700 mass spectrometer.

Optical spectroscopy

Ultraviolet–visible (UV–Vis) absorption spectra were recorded on a JASCO V-670 spectrophotometer. Steady-state fluorescence spectra were measured using a JASCO FP-6500 spectrofluorometer or an Edinburgh Instruments FS5 spectrofluorometer. Absolute photoluminescence quantum yields were determined using a Hamamatsu Photonics Quantaurus-QY system for solid-state samples, and by the integrating sphere module (SC-30) equipped with the FS5 spectrofluorometer for solution samples.

Fluorescence lifetimes in solution were measured on an Edinburgh Instruments FS5 spectrofluorometer by time-correlated single-photon counting (TCSPC) using an EPL-405 picosecond pulsed diode laser (λ_{ex} = 402.5 ± 5 nm; pulse width ≈ 75 ps; repetition rate 2.5 kHz–20 MHz). Solid-state fluorescence lifetimes for **DCBS[6]** and **DCBS[7]** were recorded using a HORIBA Fluorocube TCSPC system equipped with a NanoLED excitation source (379 nm). For all TCSPC measurements, the time-to-amplitude converter (TAC) range was set to 50 ns, and data were collected until at least 10,000 counts were accumulated in the peak channel. For **DPB[7]C** and **DPB[7]N**, both solution-phase and solid-state fluorescence lifetimes were measured on the FS5 spectrofluorometer by TCSPC using the same EPL-405 pulsed diode laser (λ_{ex} = 402.5 ± 5 nm).

All solution-phase photophysical measurements were performed using optically dilute samples (optical density ≈ 0.10 at the excitation or absorption maximum) in 1-cm quartz cuvettes at room temperature (298 K). Temperature-dependent fluorescence measurements were carried out using an Oxford Instruments OptistatDN cryostat (77–300 K) or a Unisoku CoolSpeK USP-203-A cryogenic sample chamber coupled to the FS5 spectrofluorometer.

Femtosecond transient absorption spectroscopy

Femtosecond transient absorption (fsTA) measurements were performed using a pump–probe setup. A Ti regenerative amplifier system (Spectra-Physics, Spitfire Ace; pulse duration, 120 fs; repetition rate, 1 kHz; pulse energy, 3 mJ $pulse^{-1}$; central wavelength, 800 nm) was used as the excitation source. The amplifier output was split into pump and probe beams. The pump pulse at 400 nm was generated as the second harmonic of the 800 nm fundamental. A broadband

white-light continuum probe pulse was generated in a 3 mm-thick sapphire crystal. The time delay between the pump and probe pulses was controlled using a PC-operated mechanical delay stage. After passing through the sample solutions, the probe light was dispersed by a polychromator (JASCO, CT-10; 300 grooves mm^{-1}, blazed at 500 nm) and detected with a multichannel detection system equipped with a CMOS sensor (UNISOKU, USP-PSMM-NP). The data were analyzed using a home-built Python-based program.

Single crystal X-ray diffraction (XRD) analysis

Single crystal X-ray structural analysis were obtained using a RIGAKU FR-E+with Hypix-6000 area detector. Data were measured using ω scans of 0.5° per frame for 0.1/0.3 s using Cu Kα radiation. The diffraction pattern was indexed, and the total number of runs and images was based on the strategy calculation from the program CrysAlisPro (Rigaku, V1.171.42.41a, 2022). The diffraction pattern was indexed, and the total number of runs and images was based on the strategy calculation from the program CrysAlisPro (Rigaku, V1.171.42.41a, 2022). The unit cell was refined using CrysAlisPro (Rigaku, V1.171.42.41a, 2022) on 11323 reflections, 51% of the observed reflections. Data reduction, scaling, and absorption corrections were performed using CrysAlisPro (Rigaku, V1.171.42.41a, 2022). A multi-scan absorption correction was performed using CrysAlisPro 1.171.42.41a.

Theoretical calculations

Density functional theory (DFT) calculations were performed using Gaussian 16[S1], GAMESS (September 2022 release), and Q-Chem. Geometry optimizations and frequency analyses were carried out for all minima to confirm that the optimized structures exhibited no imaginary frequencies. Excited-state calculations were conducted using time-dependent DFT (TD-DFT).

To construct the potential energy surface (PES) diagram, mixed-reference spin-flip (MRSF) TD-DFT calculations were performed. The transition state (TS) along the S_1 relaxation pathway was initially approximated by linear interpolation in internal coordinates (LIIC) between the S_1 minimum (S_{1min}) and the conical intersection (CI). This initial TS guess was subsequently refined using MRSF-TDDFT optimization. The resulting TS structure was verified by vibrational analysis to possess a single imaginary frequency. Its connectivity to the S_{1min} and INT structures was further confirmed by following the eigenvector associated with the imaginary frequency.

For the calculations shown in **Figure 8**, spin-flip TD-DFT calculations were additionally carried out using Q-Chem with the BHHLYP functional and the 6-31G(d) basis set.

S2. Materials

Unless otherwise noted, all solvents and chemicals were commercially available and used without further purification. 6-bromo-1-tetralone was purchased from BLD pharma (Shanghaiina). 6-bromo-2-tetralone, and magnesium sulfate were purchased from Sigma-Aldrich Japan (Tokyo, Japan). *p*-Toluenesulfonic acid (*p*-TsOH), Methyl triphenylphosphonium bromide, tetrakis(triphenylphosphine)palladium(0) ($Pd(PPh_3)_4$), *N*-Phenyl-bis(trifluoromethanesulfonimide) ($PhNTf_2$), [Hydroxy(tosyloxy)iodo]benzene (HTIB), 4-(Dimethylamino)phenylboronic acid, 4-Cyanophenylboronic acid, and Iodine (I_2) were

obtained from TCI (Tokyo, Japan). Potassium tert-butoxide (KO^{t}Bu), Potassium phosphate (K_3PO_4), Sodium bicarbonate ($NaHCO_3$), Ammonium chloride (NH_4Cl), Sodium thiosulfate ($Na_2S_2O_3$), Hydrochloric acid, 13 M (13 M HCl), *n*-Butyllithium (2.6 M in hexane, *n*-BuLi), Ethylene glycol, and spectrograde pure water were purchased from Kanto Chem (Tokyo, Japan). Ethylene glycol, ammonium aqueous solution (NH_3 aq), were purchased from Wako Pure Chem (Tokyo, Japan). Column chromatography was performed on silica gel (Silica Gel 60N, 63-210 μm, Kanto chemical Co., Inc.). Spectro grade solvents: *n*-hexane, toluene, dichloromethane, THF, acetonitrile were purchased from Wako Pure Chem (Tokyo, Japan).

S3. Calculation

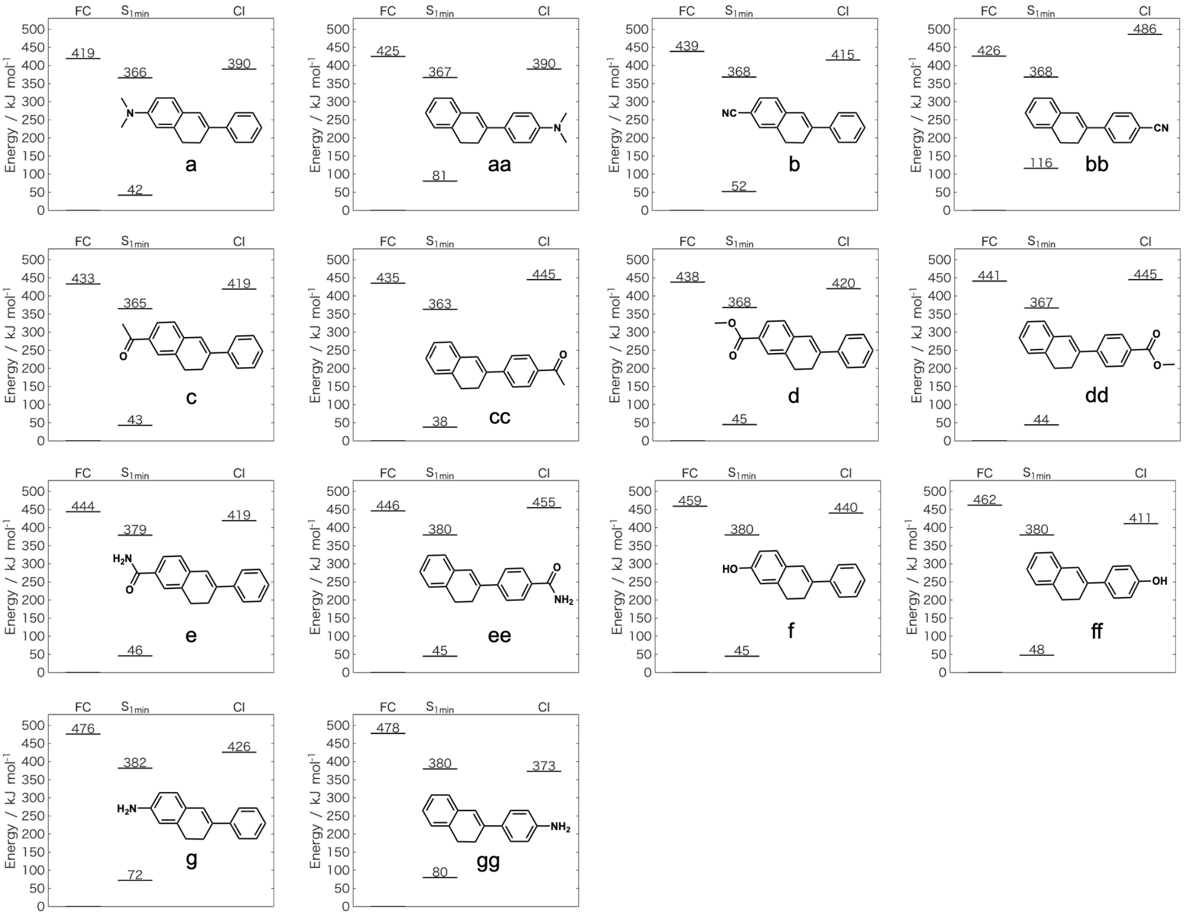

Figure S1. Computed energy profiles for the bridged stilbene (**BST6]**) derivatives investigated in this study. Energies are plotted relative to the ground state.

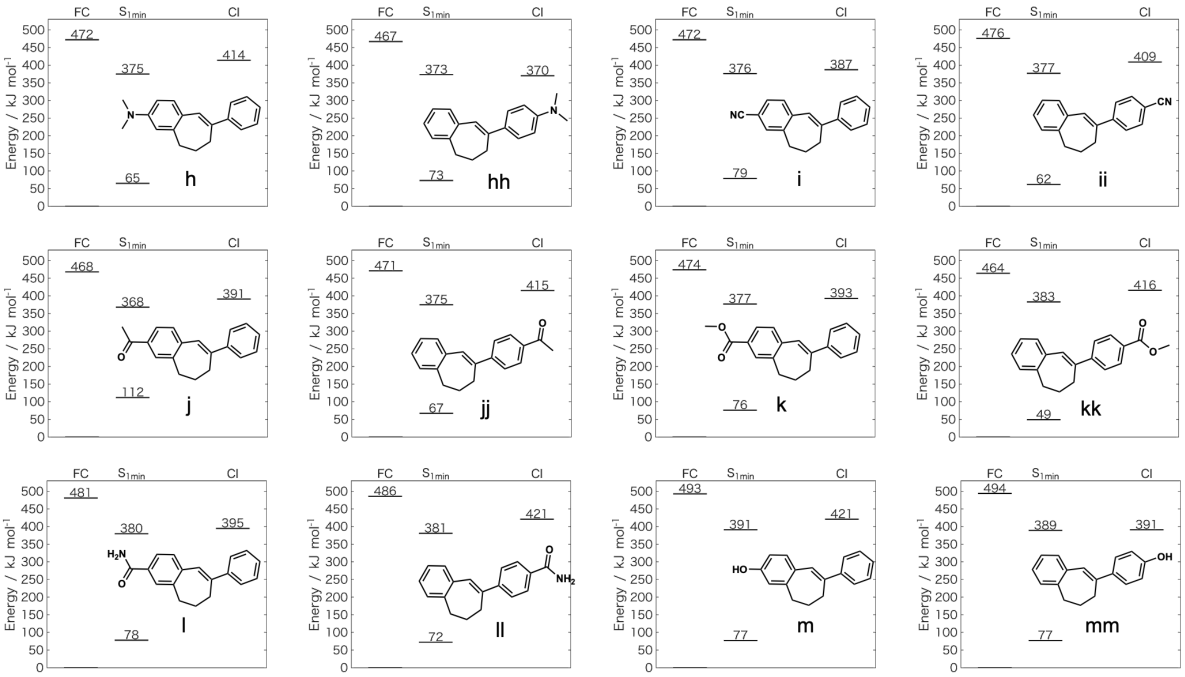

Figure S2. Computed energy profiles for the bridged stilbene (**BST[7]**) derivatives investigated in this study. Energies are plotted relative to the ground state.

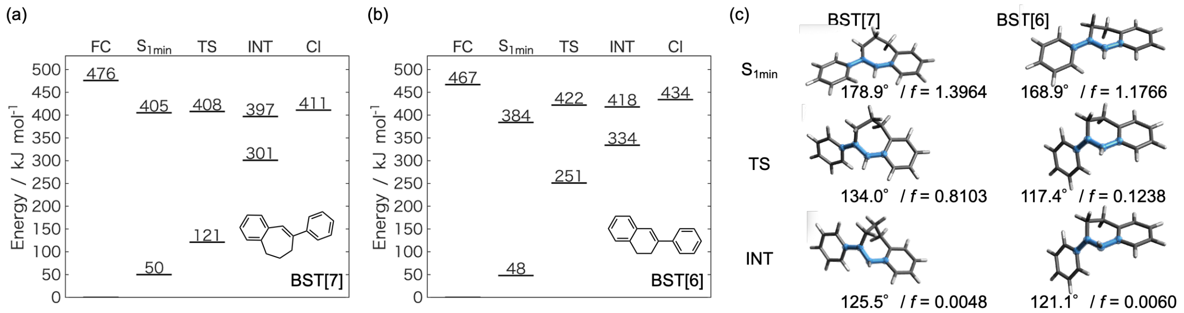

Figure S3. Energy diagrams and estimated structures of (a) BST[7], and (b) BST[6] in the gas phase. (c) Estimated structures (S_{1min}, TS, and INT) of BST[7] and BST[6]. The dihedral angles highlighted in cyan and the corresponding oscillator strengths (*f*) for each structure are indicated. All calculations were performed at the MRSF-TDDFT BHHLYP/6-31G(d) level.

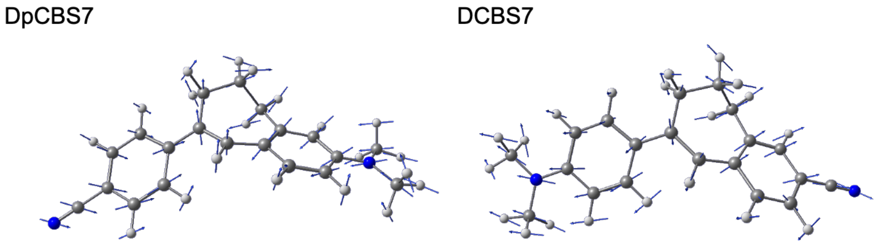

Figure S4. Visualization of the imaginary-frequency vibrational mode at the S_1 transition state (TS) for **DpCBS7** (left) and **DCBS7** (right). The displacement vectors illustrate the nuclear motion that drives the system toward the adjacent conical intersection along the reaction pathway.

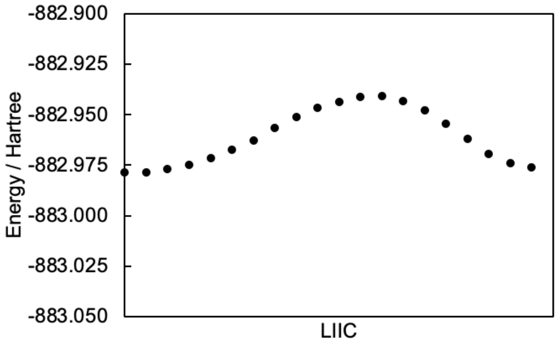

Figure S5. Potential energy surface calculated using linear interpolation in internal coordinates (LIIC) connecting S_{1min} and CI of **DCBS[7]** at the MRSF-TDDFT-BHHLYP/6-31G(d) level of theory.

Figure S4 shows that the imaginary vibrational mode of the TS for **DCBS[7]** corresponds to the structural distortion connecting the S_{1min} and INT geometries. When an IRC calculation was attempted, the trajectory deviated from the expected reaction pathway, even when a small step size of 0.1 was employed. This behavior is most likely an artifact arising from the relatively flat potential energy surface in this region. Therefore, as an alternative validation of the TS connectivity, geometries were generated by displacing the TS structure along the positive and negative directions of the imaginary vibrational mode, and each displaced structure was subsequently optimized. As a result, the structure displaced in the negative direction converged to S_{1min}, whereas that displaced in the positive direction converged to the INT structure.

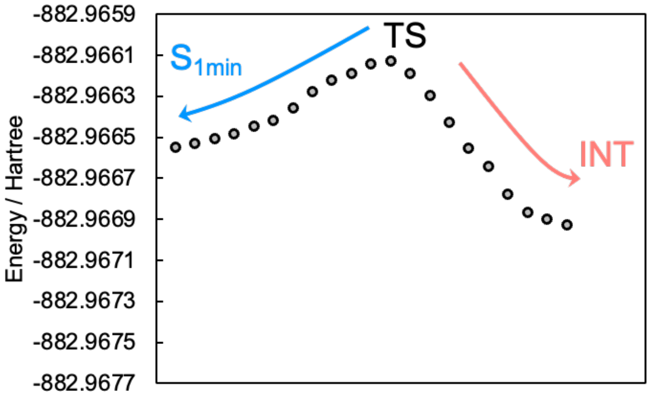

Figure S6. Linear interpolation in internal coordinates (LIIC) profiles generated by displacing the transition-state (TS) structure along the positive and negative directions of the imaginary vibrational mode.

S4. Photophysical properties

4-1. Optical properties

Table S1. Optical properties of **DCBS[6]**. Absorption coefficient (ε), maximum absorption wavelength (λ_{abs}), maximum fluorescence wavelength (λ_{fl}), Stokes shift, and fluorescence quantum yields (Φ_{fl}).

Solvent	ε / M^{-1} cm^{-1}	λ_{abs} / nm	λ_{fl} / nm	Stokes shift / nm/cm^{-1}	Φ_{fl}
n-Hexane	63000	371	440	69/4300	0.03
Toluene	52000	382	473	91/5000	0.06
THF	54000	382	491	109/5800	0.10
DCM	53000	384	500	116/6000	0.10
Acetonitrile	55000	380	521	141/7100	0.18
Solid	-	432	532	100/4400	0.18

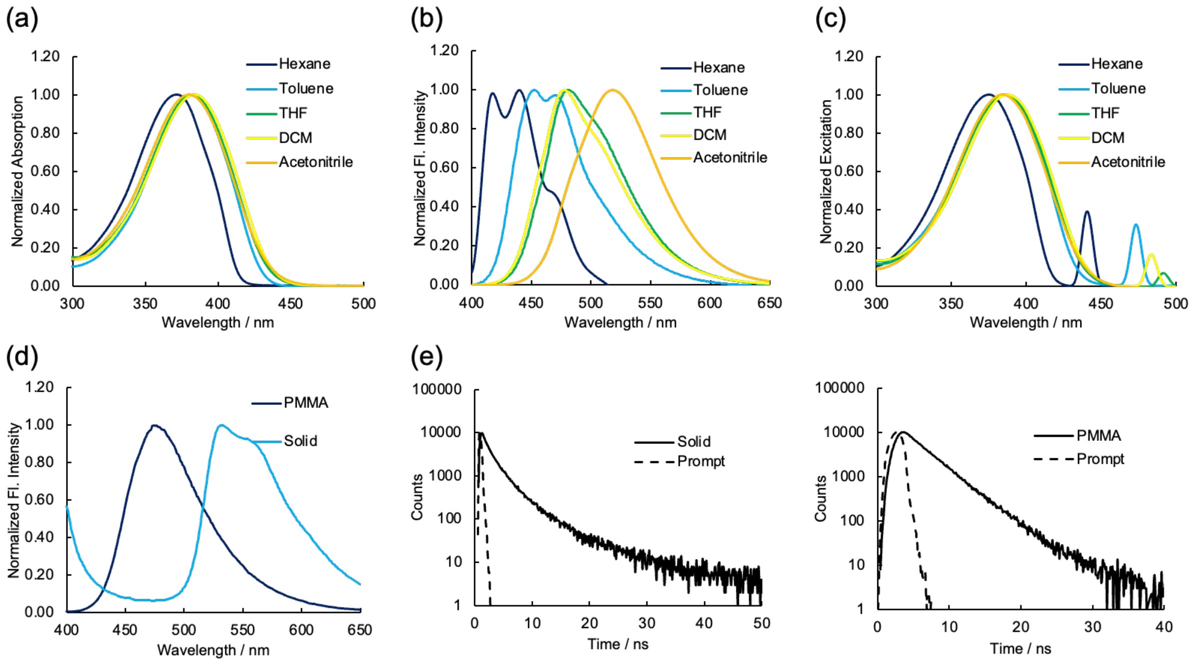

Figure S7. Optical measurement spectra of **DCBS[6]**. (a) absorption spectra.(b) fluorescence spectra. The excitation wavelength was maximum absorption wavelength in each solvent. (c) excitation spectra at maximum fluorescence wavelength (λ_{fl}). (d) fluorescence spectra in the solid-state and PMMA. (e) fluorescence lifetime decay profile in the solid-state and PMMA. (λ_{ex} = 379 nm).

Table S2. Optical properties of **DCBS[7]**. Absorption coefficient (ε), maximum absorption wavelength (λ_{abs}), maximum fluorescence wavelength (λ_{fl}), Stokes shift, and fluorescence quantum yields (Φ_{fl}).

Solvent	ε / M^{-1} cm^{-1}	λ_{abs} / nm	λ_{fl} / nm	Stokes shift / nm/cm^{-1}	Φ_{fl}
n-Hexane	35000	353	450	97/6100	0.01
Toluene	32000	363	473	110/6400	0.01
THF	31000	362	483	121/6900	0.02
DCM	38000	362	500	138/7600	0.02
Acetonitrile	33000	360	536	176/9100	0.02
Solid	-	370	519	149/7800	0.36

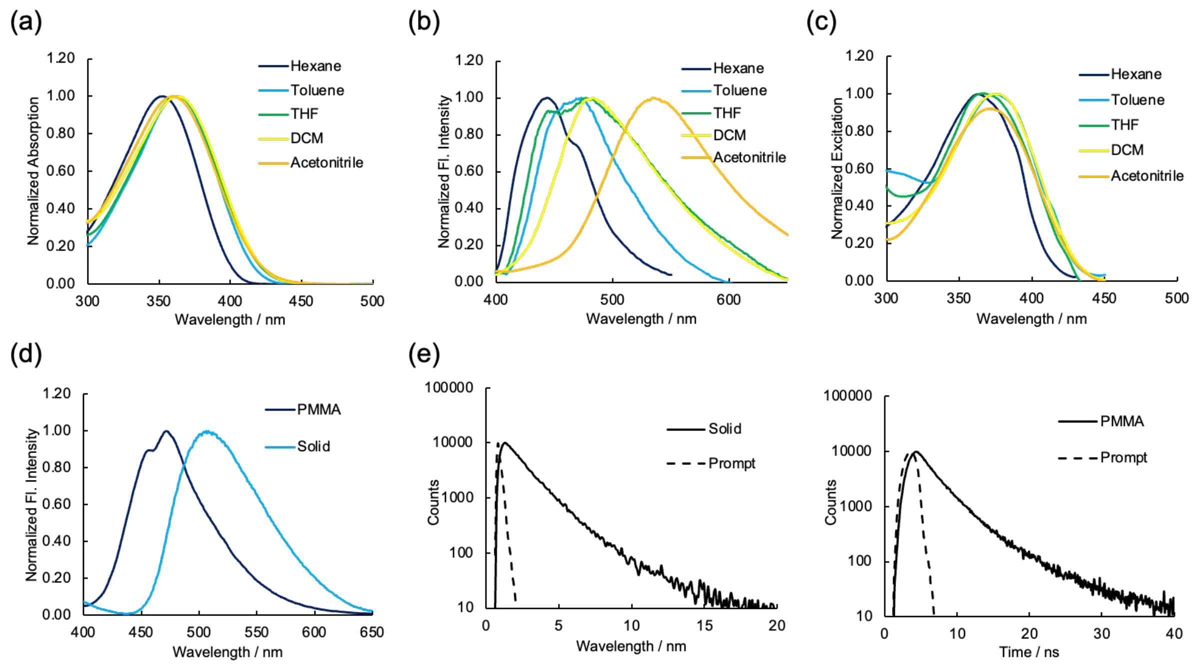

Figure S8. Optical measurement spectra of **DCBS[7]**. (a) absorption spectra.(b) fluorescence spectra. The excitation wavelength was maximum absorption wavelength in each solvent. (c) excitation spectra at maximum fluorescence wavelength (λ_{fl}). (d) fluorescence spectra in the solid-state. (e) fluorescence lifetime decay profile in the solid-state and PMMA. (λ_{ex} = 379 nm).

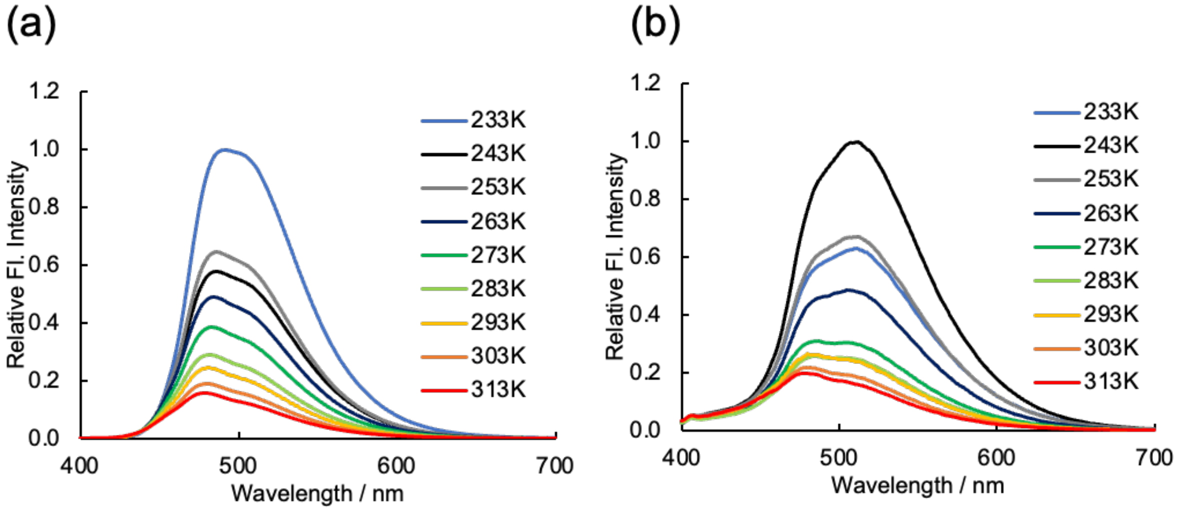

Figure S9. Temperature dependent fluorescence spectra for (a) **DCBS[6]**, and (b) **DCBS[7]** in THF in 1.0×10^{-5} M.

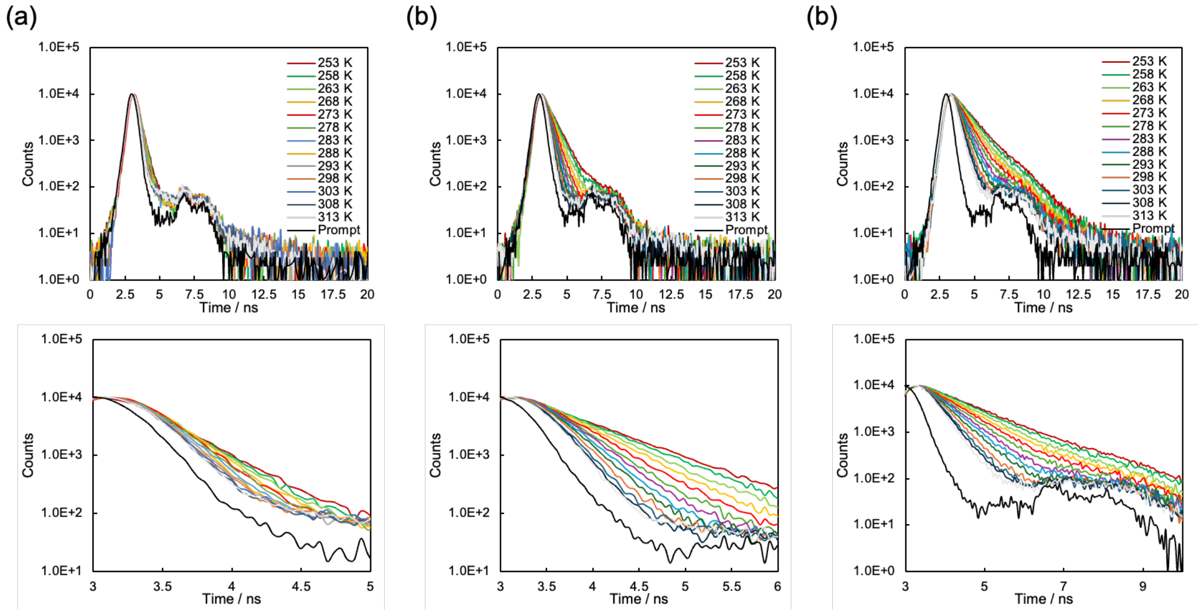

Figure S10. Temperature-dependent fluorescent lifetime profile of **DCBS[6]**: (a) in toluene, (b) in THF, and (c) in acetonitrile.

Table S3. Temperature-dependent fluorescence lifetime (τ) and radiative (k_r) and nonradiative (k_{nr}) transition rate constant for **DCBS[6]** in toluene, THF, and acetonitrile.

	Toluene		THF			Acetonitrile		
T / K	τ / ps	k_{nr} / 10^8 s^{-1}	τ / ps	k_r / 10^8 s^{-1}	k_{nr} / 10^8 s^{-1}	τ / ps	k_r / 10^8 s^{-1}	k_{nr} / 10^8 s^{-1}
313	92	109.2	192	4.28	47.9	310	4.05	28.2
308	104	96.1	187		49.1	354		24.2
303	128	78.4	225		40.1	396		21.2
298	138	72.7	234		38.5	445		18.4
293	134	74.8	287		30.5	509		15.6
288	160	62.3	301		28.9	574		13.4
283	173	57.8	336		25.4	624		12.0
278	188	53.3	365		23.1	700		10.2
273	-	-	403		20.5	809		8.3
268	183	54.6	467		17.1	851		7.7
263	194	51.7	526		14.7	917		6.9
258	220	45.5	596		12.5	1041		5.6
253	248	40.3	676		10.5	-		0.0

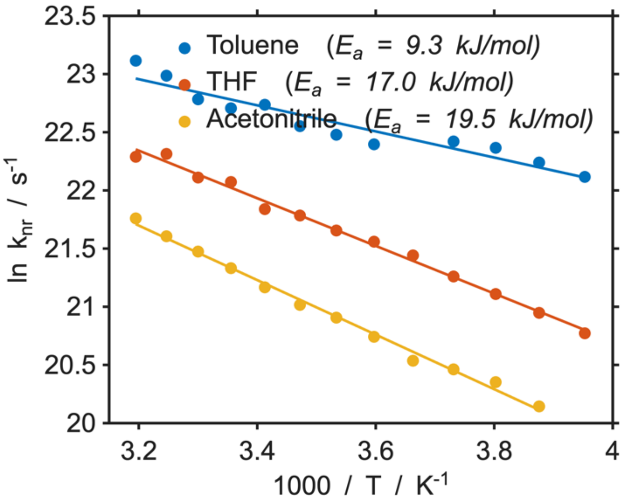

Figure S11. Arrhenius plots and nonradiative decay analysis for **DCBS[6]** in toluene (blue), THF (red), and acetonitrile (orange). Linear fits of ln k_{nr} versus 1000/T yield activation energies (E_a) of 9.3 kJ mol^{-1} (toluene), 17.0 kJ mol^{-1} (THF), and 19.5 kJ mol^{-1} (acetonitrile). The corresponding fit equations are: Toluene: ln $k_{nr} = -1.12\ (1000/T) + 26.5$ ($A = 3.38\times10^{11}\ s^{-1}$, $R^2 = 0.9124$), THF: ln $k_{nr} = -2.05\ (1000/T) + 28.9$ ($A = 3.54\times10^{12}\ s^{-1}$, $R^2 = 0.9933$), Acetonitrile: ln $k_{nr} = -2.34\ (1000/T) + 29.2$ ($A = 4.80\times10^{12}\ s^{-1}$, $R^2 = 0.9942$).

DPB[7]C and DPB[7]N

We next examined the photophysical properties of the π-extended derivatives **DPB[7]C** and **DPB[7]N**. **DPB[7]C** exhibited Φ_{fl} values of 0.09, 0.10, and 0.31 in toluene, THF, and acetonitrile, respectively, and reached Φ_{fl} = 0.88 in the solid state, clearly displaying AIEE behavior. In sharp contrast, **DPB[7]N** maintained high fluorescence efficiencies even in solution (Φ_{fl} = 0.88, 0.90, and 0.68) and further achieved Φ_{fl} = 0.95 in the solid state, establishing it as a strongly emissive dye. In both cases, the emission wavelengths were red-shifted relative to **DCBS[7]** and **DpCBS[7]**, consistent with the lowering of excited-state energy levels by π-conjugation extension,[27] in agreement with the screening and PES analyses presented in **Section 1**. Particularly noteworthy is the exceptionally high solid-state efficiency of **DPB[7]N**. Single-crystal X-ray diffraction analysis (**Figure S27**) revealed a brickwork packing motif,[28] which prevents excessive π–π overlap between adjacent chromophores. This packing arrangement effectively suppresses intermolecular nonradiative decay pathways, thereby enabling near-unity quantum yield in the solid state.

Table S4. Optical properties of **DPB[7]C**. Absorption coefficient (ε), maximum absorption wavelength (λ_{abs}), maximum fluorescence wavelength (λ_{fl}), Stokes shift, fluorescence quantum yields (Φ_{fl}), fluorescence lifetime (τ), radiative rata constant (k_r), and nonradiative rate constant (k_{nr}).

Solvent	ε / M^{-1} cm^{-1}	λ_{abs} / nm	λ_{fl} / nm	Stokes shift / nm/cm^{-1}	Φ_{fl}	τ / ns	k_r / 10^8 s^{-1}	k_{nr} /10^8 s^{-1}
n-Hexane	21000	354	458	104/6400	0.05	0.10	5.0	95
Toluene	18000	363	484	121/6900	0.09	0.15	6.0	61
THF	36000	362	552	190/9500	0.10	0.35	2.9	26
DCM	22000	360	578	218/10000	0.12	0.35	3.4	25
Acetonitrile	26000	359	622	263/12000	0.31	0.85	3.6	8.1
Solid	-		523	-	0.95	3.5	2.7	0.10

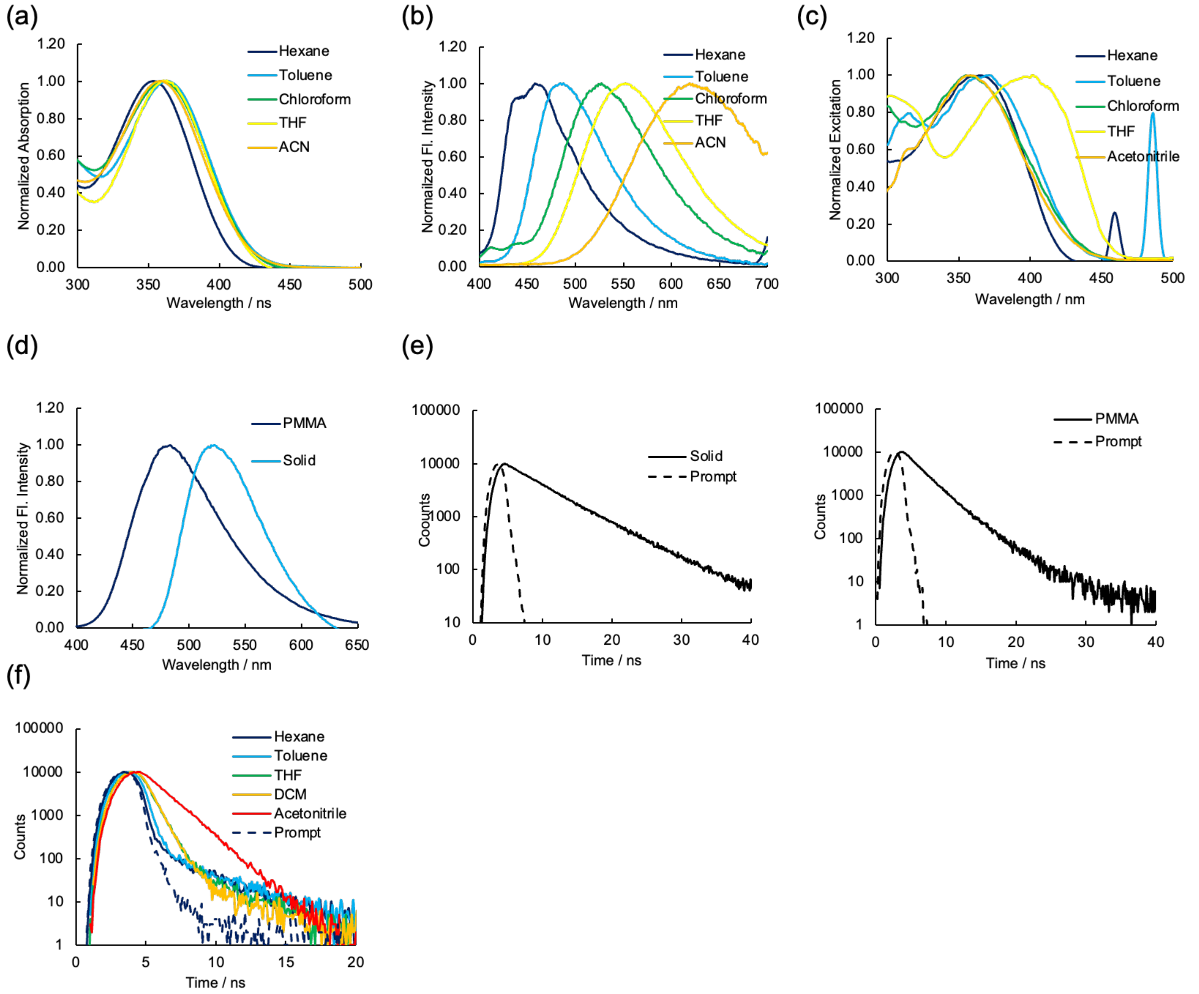

Figure S12. Optical measurement spectra of **DPB[7]C**. (a) absorption spectra.(b) fluorescence spectra. The excitation wavelength was maximum absorption wavelength in each solvent. (c) excitation spectra at maximum fluorescence wavelength (λ_{fl}). (d) fluorescence spectra in the solid-state. (e) fluorescence lifetime decay profile in the solid-state and PMMA. (λ_{ex} = 402.5 nm). (f) fluorescence lifetime decay profile in solution. (λ_{ex} = 402.5 nm).

Table S5. Optical properties of **DPB[7]N**. Absorption coefficient (ε), maximum absorption wavelength (λ_{abs}), maximum fluorescence wavelength (λ_{fl}), Stokes shift, fluorescence quantum yields (Φ_{fl}), fluorescence lifetime (τ), radiative rata constant (k_r), and nonradiative rate constant (k_{nr}).

Solvent	ε / M^{-1} cm^{-1}	λ_{abs} / nm	λ_{fl} / nm	Stokes shift / nm/cm^{-1}	Φ_{fl}	τ / ns	k_r / 10^8 s^{-1}	k_{nr} /10^8 s^{-1}
n-Hexane	38000	351	450	99/6300	0.78	0.88	8.9	2.5
Toluene	35000	361	479	118/6800	0.88	1.3	6.8	0.92
THF	31000	358	521	163/8700	0.90	2.2	4.1	0.45
DCM	39000	358	552	194/9800	0.92	2.3	4.0	0.35
Acetonitrile	41000	354	637	283/12600	0.68	2.2	3.1	1.5
Solid	-		490	-	0.88	1.6	5.4	0.70

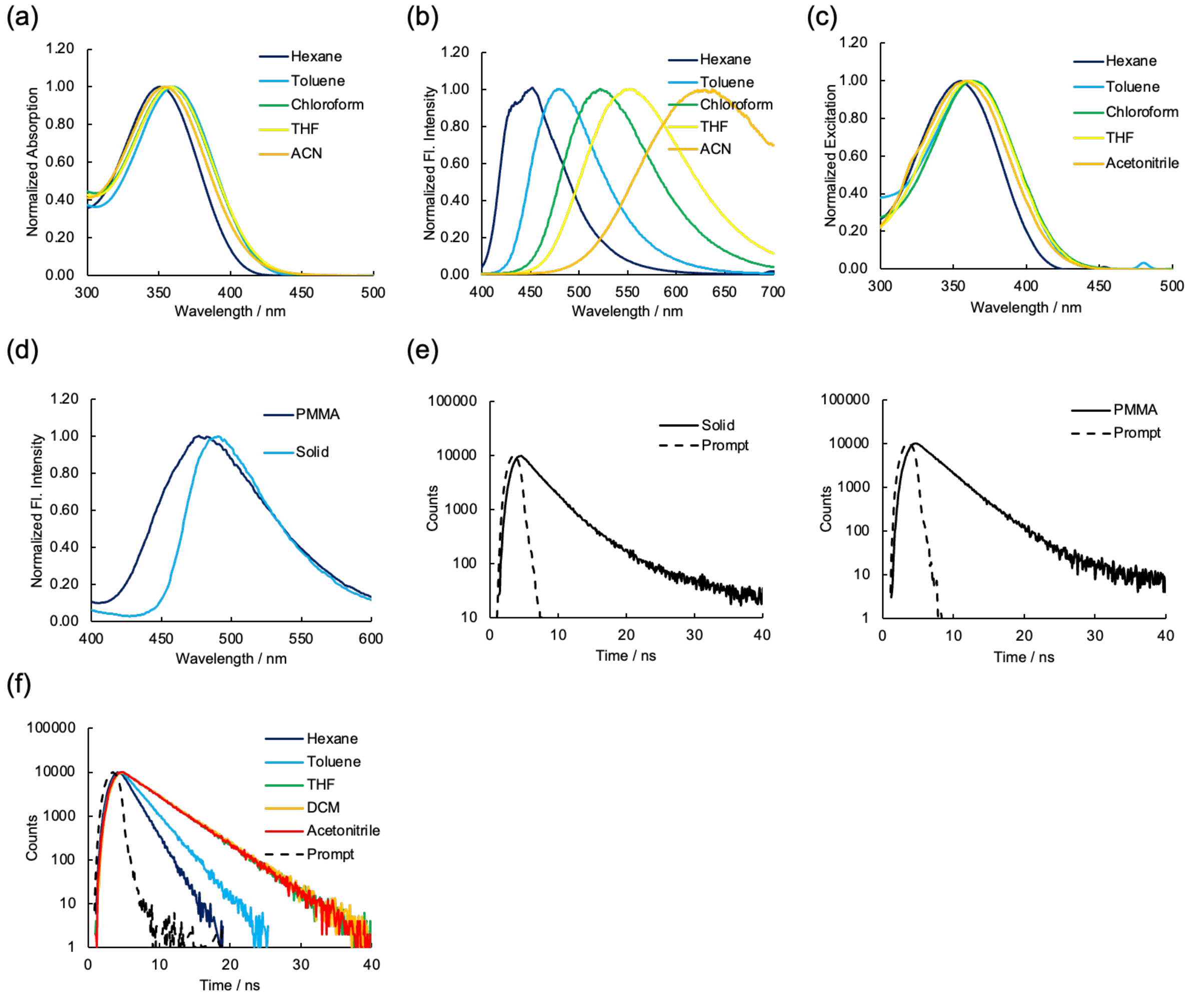

Figure S13. Optical measurement spectra of **DPB[7]N**. (a) absorption spectra.(b) fluorescence spectra. The excitation wavelength was maximum absorption wavelength in each solvent. (c) excitation spectra at maximum fluorescence wavelength (λ_{fl}). (d) fluorescence spectra in the

solid-state. (e) fluorescence lifetime decay profile in the solid-state and PMMA. (λ_{ex} = 402.5 nm). (f) fluorescence lifetime decay profile in solution. (λ_{ex} = 402.5 nm).

CpBS[6] and DBS[6]

Finally, to further validate the predictability of our screening (**Figures 3a** and **3b**), we examined molecules containing only an electron-donating or only an electron-withdrawing substituent, without a push–pull arrangement. As representative examples, **CpBS[6]** and **DBS[6]** were synthesized and their photophysical properties were investigated (**Table 2**). Because neither compound displayed solvent-dependent behavior, only the data in THF are presented. **CpBS[6]**, which was predicted not to be an AIE/AIEE candidate, showed a relatively high fluorescence quantum yield in solution (Φ_{fl} = 0.88), but its emission efficiency decreased substantially in the solid state (Φ_{fl} = 0.21). By contrast, **DBS[6]**, predicted as a promising AIE/AIEE candidate, exhibited weak fluorescence in solution (Φ_{fl} = 0.06) but a markedly enhanced efficiency in the solid state (Φ_{fl} = 0.38), demonstrating a clear AIE effect. These contrasting behaviors strongly corroborate the theoretical screening based on CI energy landscapes, providing direct experimental validation of substituent-dependent control of CI accessibility.

Table S6. Optical properties of **DBS[6]** and **CpBS[6]**. Absorption coefficient (ε), maximum absorption wavelength (λ_{abs}), maximum fluorescence wavelength (λ_{fl}) and fluorescence quantum yields (Φ_{fl}) in THF.

Entry	ε / M^{-1} cm^{-1}	λ_{abs} / nm	λ_{fl} / nm	Stokes shift / nm/cm^{-1}	Φ_{fl}
DBS[6]	31000	344	431	87/5870	0.06
CpBS[6]	34000	331	401	70/5270	0.88

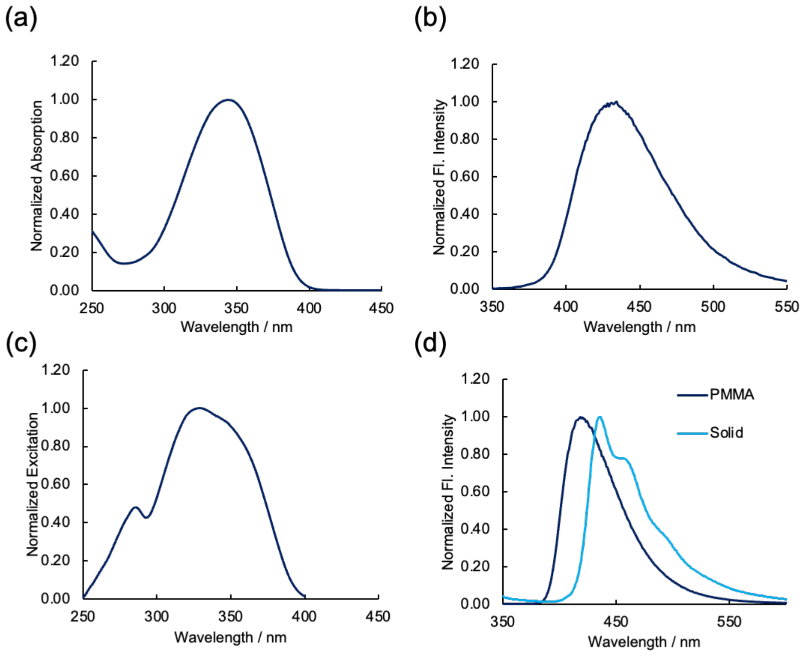

Figure S14. Optical measurement spectra of **DBS[6]**. (a) absorption spectra.(b) fluorescence spectra. The excitation wavelength was maximum absorption wavelength in THF. (c) excitation spectra at maximum fluorescence wavelength (λ_{fl}). (d) fluorescence spectra in the solid-state and PMMA.

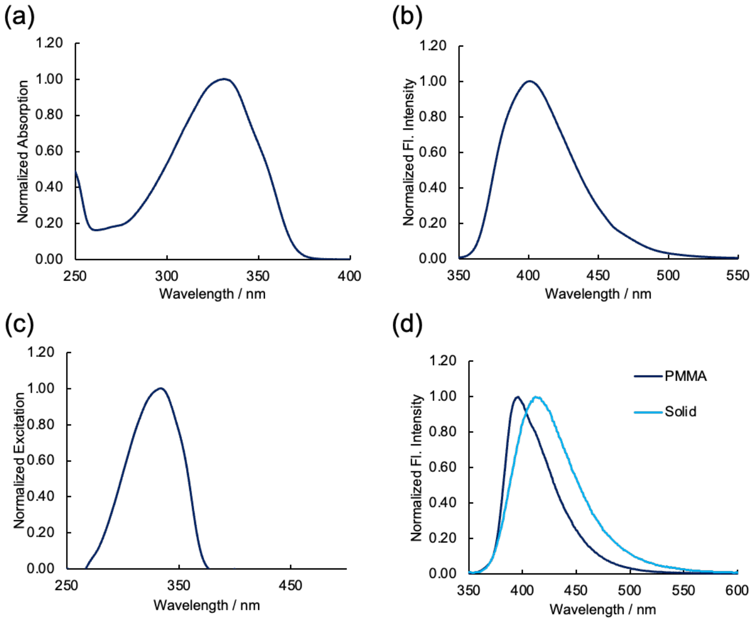

Figure S15. Optical measurement spectra of **CpBS[[6]**. (a) absorption spectra.(b) fluorescence spectra. The excitation wavelength was maximum absorption wavelength in THF. (c) excitation spectra at maximum fluorescence wavelength (λ_{fl}). (d) fluorescence spectra in the solid-state and PMMA.

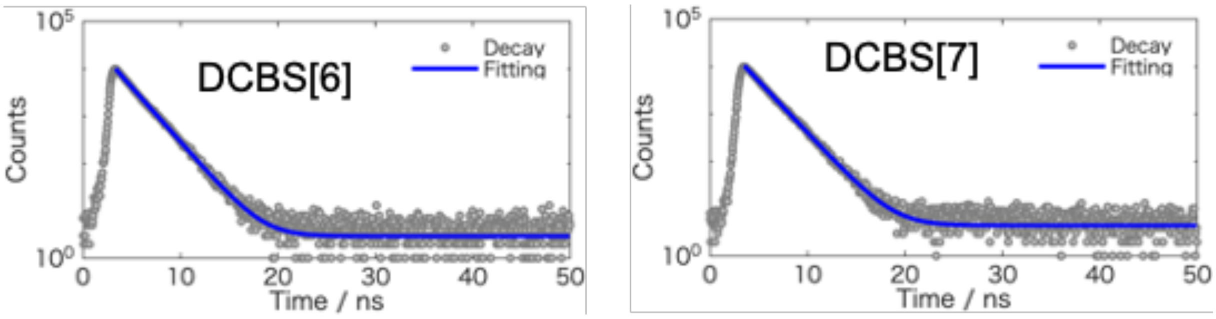

Figure S16. Fluorescence lifetime decay profiles and fitting curves of **DCBS[6]** and **DCBS[7]** measured in MeTHF at 78 K. The gray circles represent the experimental decay traces, and the solid blue lines show the corresponding fitting results. The decay curves were analyzed using a multi-exponential model to extract the fluorescence lifetimes.

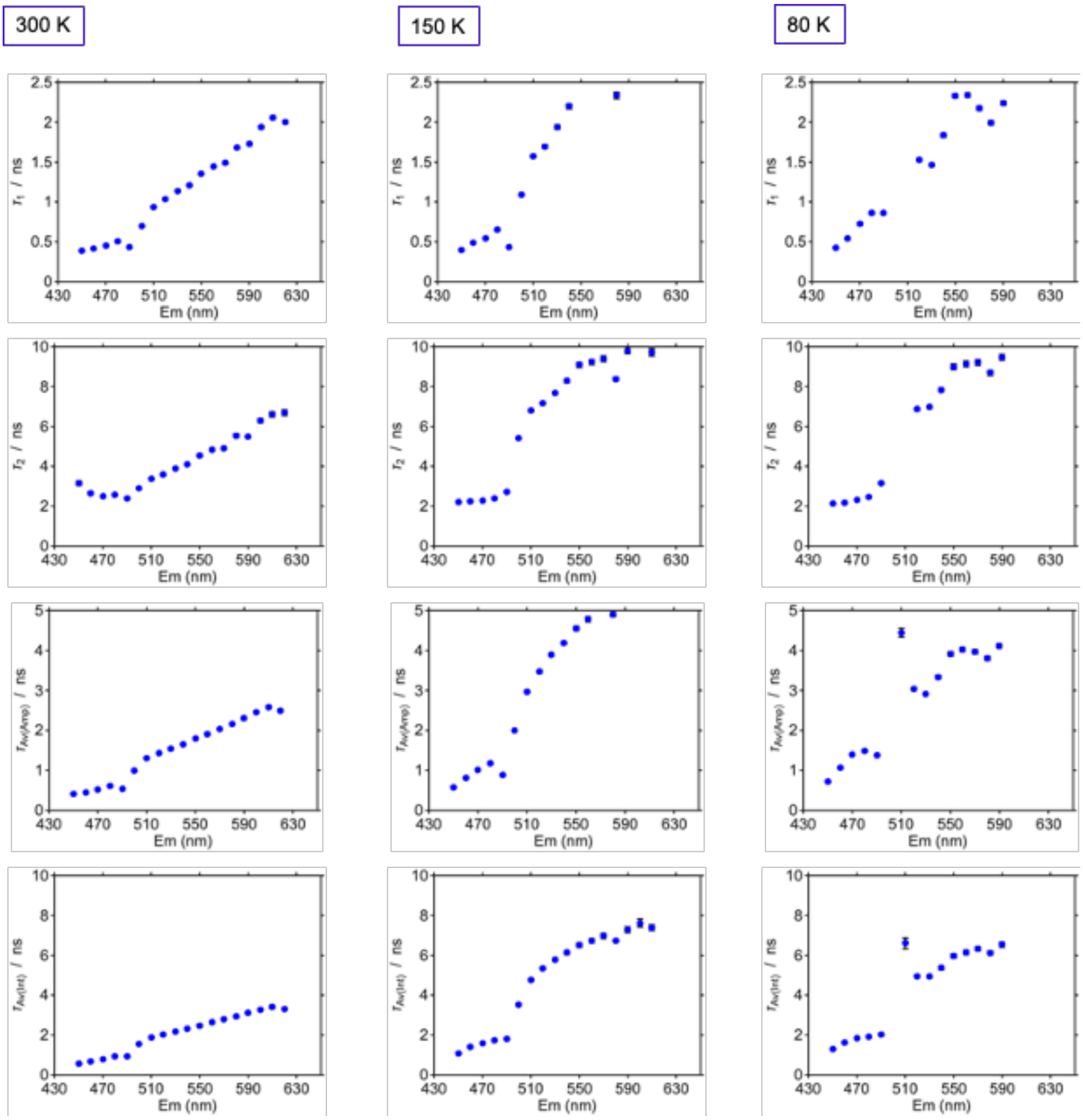

Figure S17. Emission-wavelength (λ_{em}) dependence of fluorescence lifetimes of **DCBS[6]** measured at different temperatures. The data were recorded at 300 K (left column), 150 K (middle column), and 80 K (right column). From top to bottom, the panels show the variation of the short lifetime component (τ_1), the long lifetime component (τ_2), the amplitude ratio (B_2/B_1), and the amplitude-weighted average lifetime (τ_{avg}) as a function of λem. All lifetimes were obtained by multi-exponential tail fitting of the time-resolved fluorescence decay profiles.

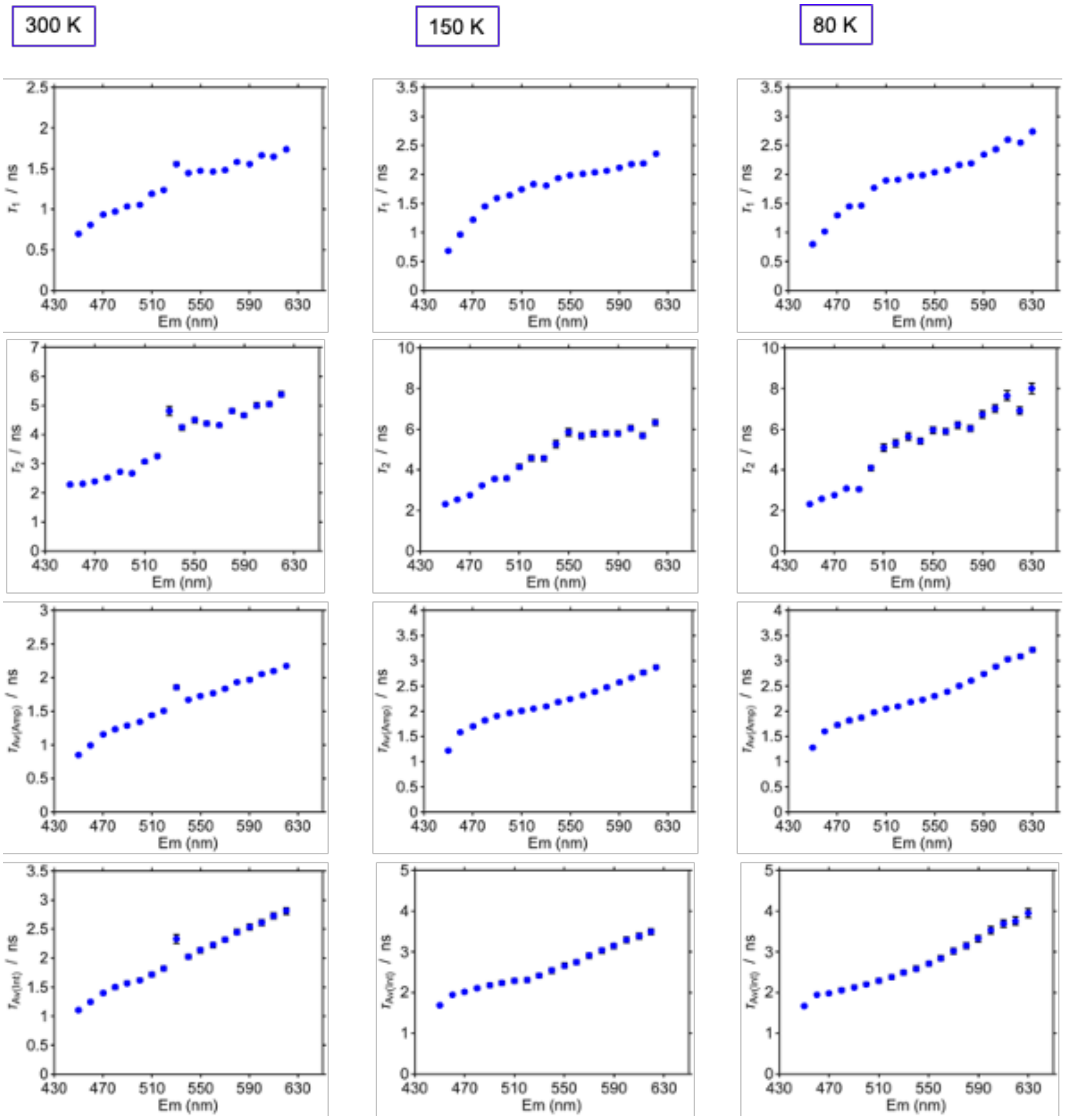

Figure S18. Emission-wavelength (λ_{em}) dependence of fluorescence lifetimes of **DCBS[7]** measured at different temperatures. The data were recorded at 300 K (left column), 150 K (middle column), and 80 K (right column). From top to bottom, the panels show the variation of the short lifetime component (τ_1), the long lifetime component (τ_2), the amplitude ratio (B_2/B_1), and the amplitude-weighted average lifetime (τ_{avg}) as a function of λem. All lifetimes were obtained by multi-exponential tail fitting of the time-resolved fluorescence decay profiles.

Table S7. Emission-wavelength-dependent fluorescence lifetime fitting parameters of **DCBS[6]** measured at 80 K. The fluorescence decay curves recorded at each emission wavelength (λ_{em}) were analyzed using a bi-exponential model, yielding the lifetimes τ_1 and τ_2, their standard deviations, and the corresponding pre-exponential factors (B_1 and B_2). The amplitude-weighted average lifetime $\langle\tau\rangle_{Amp}$ and the intensity-weighted average lifetime $\langle\tau\rangle_{Int}$,

together with their standard deviations, are also listed. The goodness of fit is evaluated by the reduced chi-square value (χ^2).

Em / nm	τ_1	τ_1 Std. Dev.	B_1	τ_2	τ_2 Std. Dev.	B_2	$\langle\tau\rangle_{Amp}$	$\langle\tau\rangle_{Amp}$ Std. Dev.	$\langle\tau\rangle$Int	$\langle\tau\rangle$Int Std. Dev.	χ^2
450	0.42	0.01	4893	2.15	0.02	1009	0.72	0.01	1.31	0.02	1.13
460	0.54	0.01	3933	2.16	0.01	1953	1.07	0.01	1.61	0.01	1.21
470	0.72	0.02	2911	2.31	0.02	2171	1.40	0.02	1.84	0.02	1.30
480	0.86	0.02	3158	2.48	0.02	2003	1.49	0.02	1.90	0.02	1.37
490	0.86	0.01	4187	3.15	0.03	1213	1.37	0.02	2.04	0.03	1.97
500	0.88	0.03	2964	3.17	0.10	2124	2.34	0.09	5.66	0.61	1.06
510	2.95	0.05	1952	10.85	0.39	457	4.45	0.11	6.61	0.27	1.26
520	1.53	0.02	3347	6.87	0.07	1328	3.04	0.03	4.95	0.06	1.89
530	1.46	0.02	3505	6.97	0.07	1253	2.91	0.03	4.93	0.06	1.73
540	1.83	0.02	3502	7.84	0.10	1171	3.34	0.04	5.37	0.08	1.69
550	2.33	0.03	3578	9.00	0.15	1113	3.92	0.05	5.97	0.12	1.40
560	2.34	0.03	3575	9.15	0.15	1167	4.02	0.05	6.16	0.12	1.51
570	2.17	0.03	3345	9.20	0.14	1149	3.97	0.05	6.34	0.12	1.61
580	2.00	0.03	3390	8.69	0.12	1255	3.80	0.05	6.13	0.10	1.62
590	2.24	0.03	3427	9.47	0.15	1196	4.11	0.06	6.55	0.13	1.47
600	0.85	0.06	1435	3.87	0.11	3084	4.74	0.25	11.36	1.40	1.10
610	0.75	0.05	1521	3.99	0.10	2907	5.06	0.33	14.36	2.25	1.07
620	0.48	0.01	2778	3.82	0.08	2203	3.58	0.22	14.40	2.16	1.05

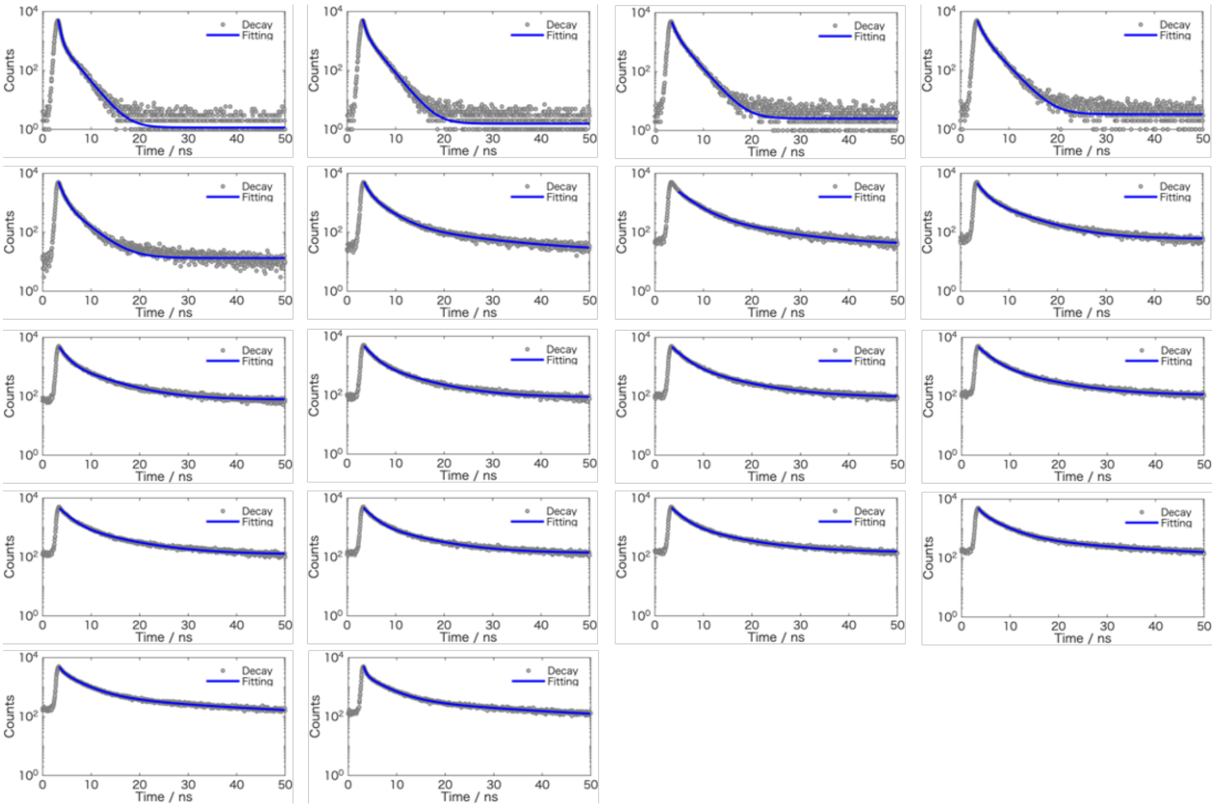

Figure S19. Representative fluorescence decay fitting curves of **DCBS[6]** recorded at 80 K for different emission detection wavelengths (λ_{em}).

Table S8. Emission-wavelength-dependent fluorescence lifetime fitting parameters of **DCBS[6]** measured at 160 K.

Em / nm	τ_1	τ_1 Std. Dev.	B_1	τ_2	τ_2 Std. Dev.	B_2	$\langle\tau\rangle_{Amp}$	$\langle\tau\rangle_{Amp}$ Std. Dev.	$\langle\tau\rangle$Int	$\langle\tau\rangle$Int Std. Dev.	χ^2
450	0.40	0.00	5775	2.20	0.02	611	0.57	0.01	1.07	0.02	1.40
460	0.49	0.01	4983	2.26	0.02	1110	0.81	0.01	1.39	0.02	1.26
470	0.55	0.01	4261	2.29	0.02	1541	1.01	0.01	1.60	0.02	1.23
480	0.65	0.01	4013	2.40	0.02	1731	1.18	0.01	1.73	0.02	1.41
490	0.43	0.01	4287	2.71	0.02	1053	0.88	0.01	1.81	0.02	1.72
500	1.09	0.01	3581	5.41	0.05	946	2.00	0.02	3.54	0.04	2.11
510	1.57	0.02	3503	6.79	0.06	1286	2.97	0.03	4.78	0.05	1.92
520	1.70	0.02	3219	7.16	0.06	1543	3.47	0.03	5.36	0.05	1.59
530	1.94	0.03	2970	7.69	0.07	1524	3.89	0.04	5.79	0.06	1.74
540	2.20	0.03	3123	8.29	0.09	1514	4.19	0.04	6.13	0.08	1.67
550	2.58	0.03	3233	9.10	0.12	1397	4.55	0.06	6.51	0.10	1.56
560	2.71	0.04	3186	9.25	0.13	1484	4.79	0.06	6.72	0.11	1.53
570	2.90	0.05	2990	9.40	0.14	1551	5.12	0.07	6.98	0.12	1.34
580	2.34	0.04	2581	8.39	0.09	1890	4.90	0.06	6.72	0.08	1.90
590	3.01	0.05	2917	9.81	0.17	1499	5.32	0.08	7.27	0.14	1.73

600	3.41	0.06	2969	10.61	0.24	1343	5.66	0.11	7.62	0.19	1.24
610	3.06	0.06	2647	9.73	0.17	1550	5.52	0.09	7.40	0.15	1.60
620	0.34	0.01	3388	4.76	0.10	2022	3.05	0.18	10.33	1.40	1.20

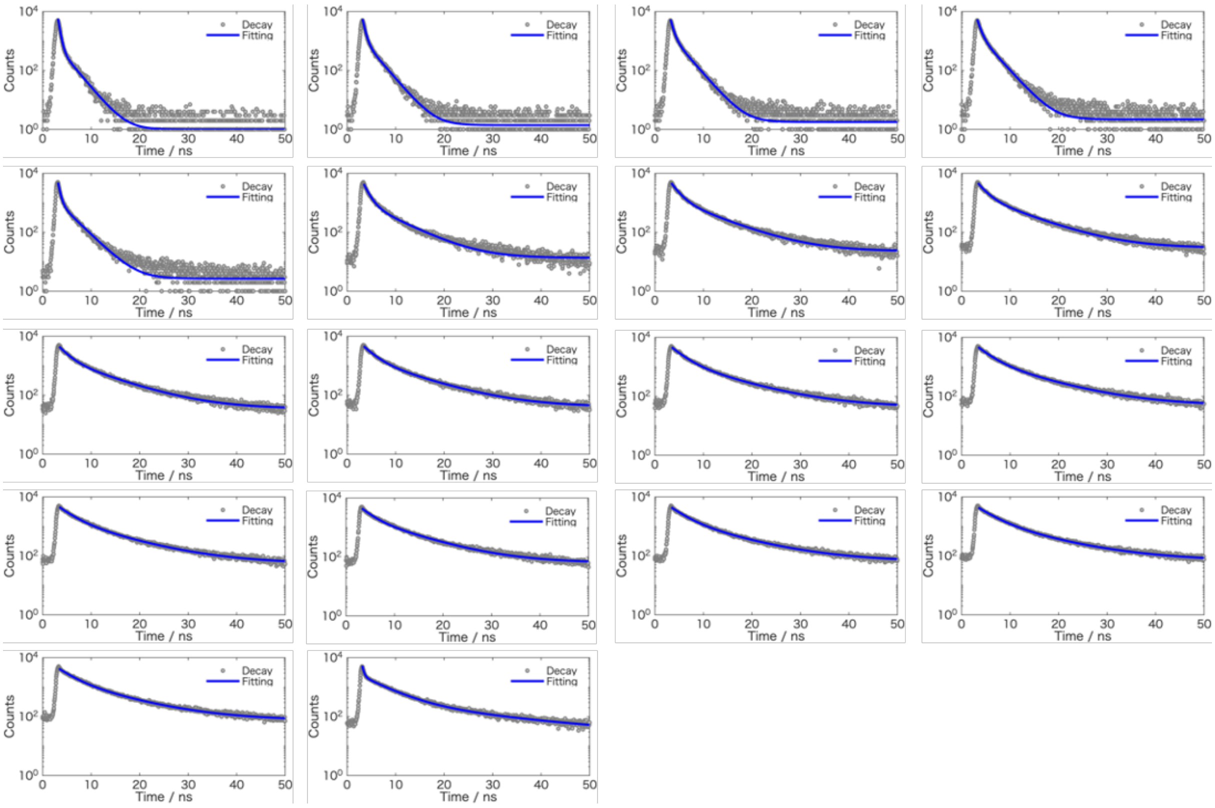

Figure S20. Representative fluorescence decay fitting curves of **DCBS[6]** recorded at 150 K for different emission detection wavelengths (λ_{em}).

Table S9. Emission-wavelength-dependent fluorescence lifetime fitting parameters of **DCBS[6]** measured at 300 K.

Em / nm	τ_1	τ_1 Std. Dev.	B_1	τ_2	τ_2 Std. Dev.	B_2	$\langle\tau\rangle_{Amp}$	$\langle\tau\rangle_{Amp}$ Std. Dev.	$\langle\tau\rangle$Int	$\langle\tau\rangle$Int Std. Dev.	χ^2
450	0.39	0.00	7093	3.15	0.10	57	0.41	0.00	0.56	0.02	3.60
460	0.41	0.00	6624	2.65	0.06	133	0.46	0.00	0.67	0.02	2.35
470	0.45	0.00	6270	2.49	0.04	232	0.52	0.00	0.80	0.02	1.96
480	0.50	0.00	6228	2.58	0.04	323	0.61	0.01	0.94	0.02	1.76
490	0.43	0.00	5857	2.39	0.03	359	0.55	0.01	0.93	0.02	1.06
500	0.70	0.01	5081	2.92	0.03	776	0.99	0.01	1.56	0.02	1.55
510	0.94	0.01	4616	3.38	0.04	832	1.31	0.01	1.90	0.03	1.74
520	1.04	0.01	4753	3.58	0.04	895	1.44	0.01	2.04	0.03	1.66
530	1.14	0.01	4766	3.90	0.05	807	1.54	0.02	2.15	0.03	1.71
540	1.21	0.01	4768	4.09	0.05	863	1.65	0.02	2.30	0.03	1.72
550	1.35	0.01	4743	4.54	0.06	774	1.80	0.02	2.48	0.04	1.69

560	1.44	0.01	4641	4.83	0.06	751	1.92	0.02	2.63	0.04	1.69
570	1.49	0.01	4283	4.91	0.06	818	2.04	0.02	2.81	0.04	1.49
580	1.68	0.01	4751	5.54	0.08	683	2.17	0.02	2.92	0.05	1.59
590	1.73	0.02	4294	5.50	0.08	774	2.30	0.03	3.10	0.05	1.40
600	1.94	0.02	4602	6.29	0.11	629	2.46	0.03	3.28	0.06	1.27
610	2.06	0.02	4527	6.62	0.13	593	2.59	0.03	3.41	0.07	1.24
620	2.00	0.02	4458	6.70	0.13	519	2.49	0.03	3.32	0.07	1.43

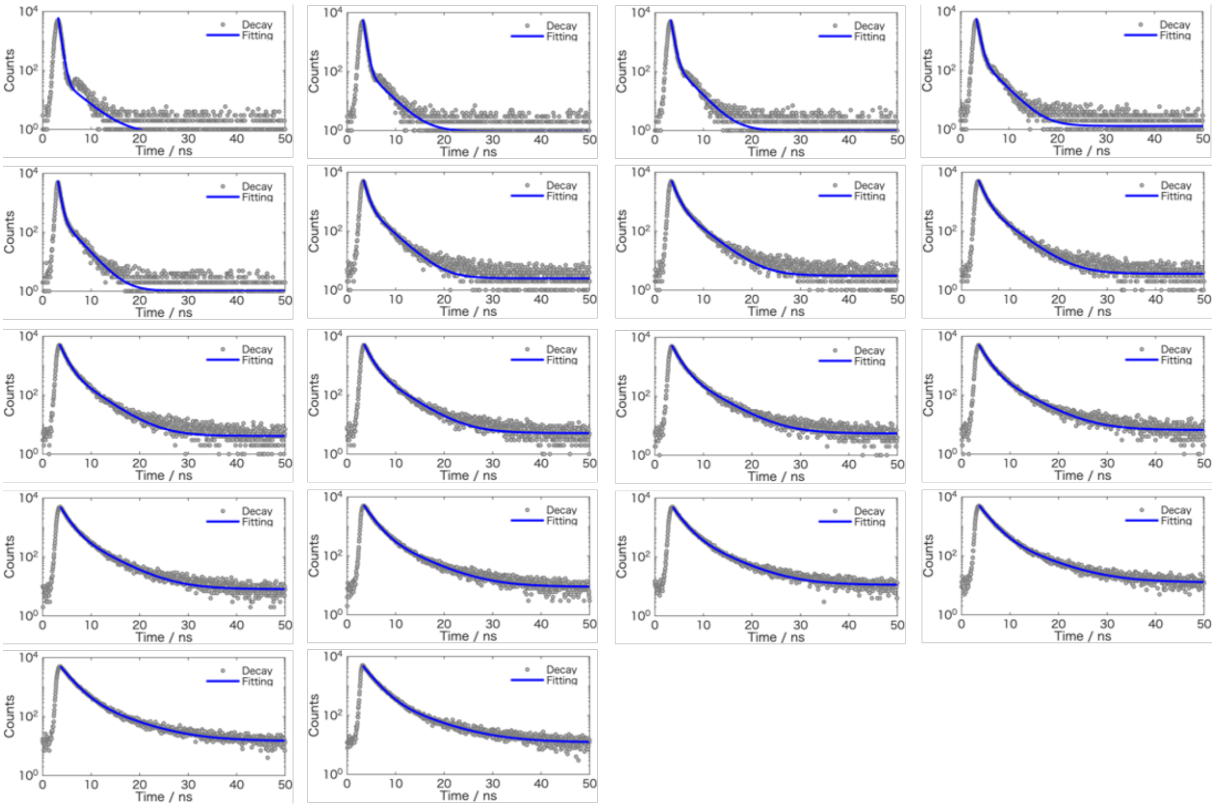

Figure S21. Representative fluorescence decay fitting curves of **DCBS[6]** recorded at 300 K for different emission detection wavelengths (λ_{em}).

Table S10. Emission-wavelength-dependent fluorescence lifetime fitting parameters of **DCBS[7]** measured at 80 K.

Em / nm	τ_1	τ_1 Std. Dev.	B_1	τ_2	τ_2 Std. Dev.	B_2	$\langle\tau\rangle_{Amp}$	$\langle\tau\rangle_{Amp}$ Std. Dev.	$\langle\tau\rangle$Int	$\langle\tau\rangle$Int Std. Dev.	χ^2
450	0.79	0.02	3633	2.31	0.02	1731	1.28	0.02	1.67	0.02	1.12
460	1.02	0.02	3286	2.56	0.03	1990	1.60	0.02	1.95	0.03	1.09
470	1.30	0.03	3896	2.77	0.05	1597	1.72	0.03	1.98	0.04	1.35
480	1.45	0.02	4026	3.07	0.07	1160	1.81	0.03	2.06	0.05	1.21
490	1.47	0.03	3877	3.05	0.06	1351	1.87	0.04	2.13	0.05	1.48
500	1.77	0.02	5014	4.09	0.13	507	1.98	0.03	2.21	0.05	1.49
510	1.89	0.01	5303	5.08	0.18	282	2.05	0.02	2.29	0.05	1.48

520	1.91	0.01	5036	5.31	0.18	293	2.09	0.02	2.38	0.05	1.49
530	1.98	0.01	4991	5.64	0.19	286	2.18	0.02	2.49	0.06	1.46
540	1.99	0.02	5044	5.42	0.16	392	2.23	0.03	2.59	0.06	1.30
550	2.04	0.01	5147	5.96	0.17	363	2.30	0.03	2.71	0.06	1.46
560	2.08	0.02	4841	5.89	0.16	426	2.39	0.03	2.84	0.07	1.40
570	2.16	0.02	4644	6.21	0.17	436	2.51	0.03	3.02	0.07	1.38
580	2.19	0.02	4734	6.04	0.15	565	2.60	0.03	3.14	0.07	1.22
590	2.34	0.02	4778	6.73	0.18	483	2.74	0.03	3.33	0.08	1.09
600	2.43	0.02	4851	7.03	0.19	528	2.88	0.04	3.53	0.09	1.26
610	2.60	0.02	5150	7.66	0.23	486	3.03	0.04	3.70	0.10	1.43
620	2.55	0.02	4663	6.92	0.18	658	3.09	0.04	3.76	0.10	1.27
630	2.74	0.02	4896	8.01	0.26	499	3.22	0.04	3.95	0.11	1.08

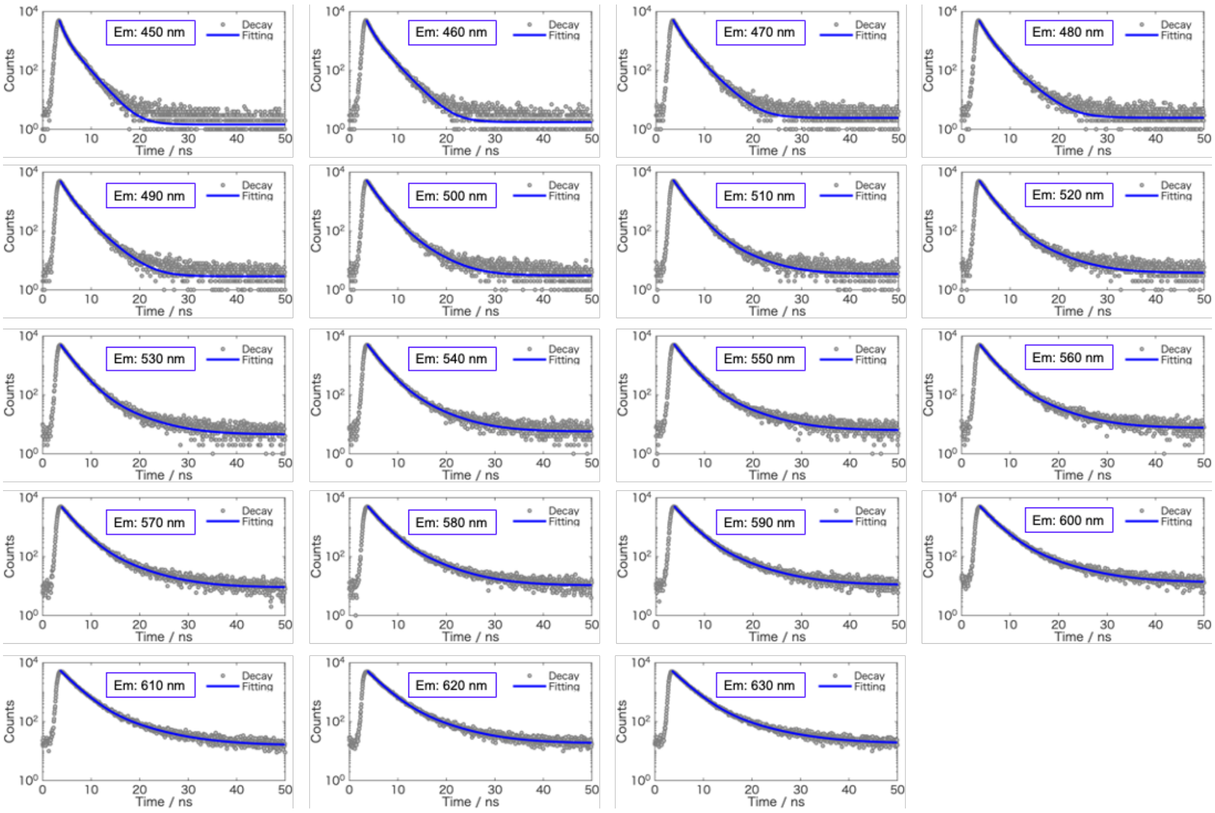

Figure S22. Representative fluorescence decay fitting curves of **DCBS[7]** recorded at 80 K for different emission detection wavelengths (λ_{em}).

Table S11. Emission-wavelength-dependent fluorescence lifetime fitting parameters of **DCBS[7]** measured at 150 K.

Em / nm	τ_1	τ_1 Std. Dev.	B_1	τ_2	τ_2 Std. Dev.	B_2	$\langle\tau\rangle_{Amp}$	$\langle\tau\rangle_{Amp}$ Std. Dev.	$\langle\tau\rangle_{Int}$	$\langle\tau\rangle_{Int}$ Std. Dev.	χ^2
450	0.68	0.01	3687	2.31	0.02	1812	1.22	0.01	1.70	0.02	1.08
460	0.96	0.02	3331	2.53	0.02	2160	1.58	0.02	1.95	0.02	1.19

470	1.21	0.02	3738	2.77	0.04	1727	1.71	0.03	2.01	0.03	1.27
480	1.45	0.02	4171	3.24	0.06	1088	1.82	0.03	2.11	0.04	1.38
490	1.59	0.02	4550	3.57	0.08	866	1.91	0.03	2.18	0.05	1.48
500	1.64	0.02	4392	3.58	0.09	885	1.96	0.03	2.23	0.05	1.42
510	1.74	0.02	4653	4.16	0.11	576	2.01	0.03	2.29	0.05	1.41
520	1.83	0.02	4806	4.58	0.15	409	2.05	0.03	2.31	0.06	1.39
530	1.81	0.02	4650	4.56	0.12	533	2.09	0.03	2.43	0.05	1.36
540	1.94	0.02	4825	5.27	0.17	390	2.18	0.03	2.54	0.06	1.17
550	1.99	0.01	4936	5.86	0.17	352	2.24	0.03	2.66	0.06	1.31
560	2.01	0.02	4931	5.68	0.15	438	2.31	0.03	2.75	0.06	1.35
570	2.03	0.02	4844	5.78	0.13	516	2.39	0.03	2.90	0.06	1.38
580	2.06	0.02	4553	5.79	0.13	569	2.48	0.03	3.03	0.07	1.12
590	2.12	0.02	4595	5.79	0.13	650	2.57	0.03	3.14	0.07	1.30
600	2.18	0.02	4634	6.05	0.13	669	2.67	0.03	3.29	0.07	1.23
610	2.19	0.03	4254	5.69	0.12	847	2.77	0.04	3.38	0.07	1.29
620	2.35	0.02	4581	6.33	0.15	686	2.87	0.04	3.50	0.08	1.23

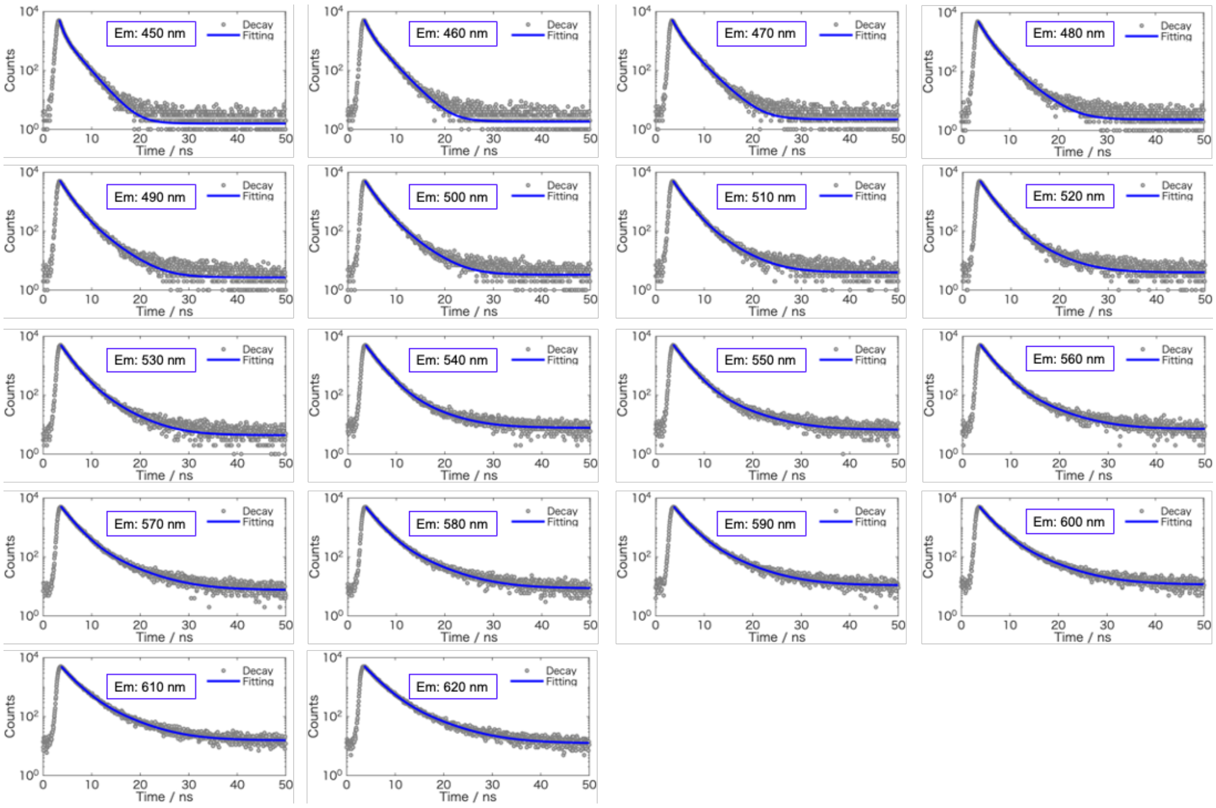

Figure S23. Representative fluorescence decay fitting curves of **DCBS[7]** recorded at 150 K for different emission detection wavelengths (λ_{em}).

Table S12. Emission-wavelength-dependent fluorescence lifetime fitting parameters of **DCBS[7]** measured at 300 K.

Em / nm	τ_1	τ_1 Std. Dev.	B_1	τ_2	τ_2 Std. Dev.	B_2	$<\tau>_{Amp}$	$<\tau>_{Amp}$ Std. Dev.	$<\tau>$Int	$<\tau>$Int Std. Dev.	χ^2
450	0.70	0.01	5428	2.27	0.04	570	0.85	0.01	1.10	0.02	1.39
460	0.80	0.01	4985	2.30	0.04	729	1.00	0.01	1.25	0.02	1.33
470	0.93	0.01	5023	2.39	0.04	914	1.16	0.02	1.40	0.03	1.52
480	0.97	0.01	4722	2.52	0.04	943	1.23	0.02	1.50	0.03	1.42
490	1.03	0.01	4680	2.72	0.05	833	1.29	0.02	1.57	0.03	1.45
500	1.06	0.01	4587	2.67	0.05	973	1.34	0.02	1.62	0.03	1.46
510	1.19	0.01	4813	3.07	0.06	728	1.44	0.02	1.72	0.04	1.55
520	1.24	0.01	4835	3.26	0.06	744	1.51	0.02	1.82	0.04	1.51
530	1.56	0.02	2098	4.81	0.15	212	1.86	0.03	2.33	0.07	1.32
540	1.45	0.01	5276	4.24	0.09	467	1.67	0.02	2.02	0.04	1.68
550	1.48	0.01	5043	4.50	0.09	460	1.73	0.02	2.13	0.04	1.55
560	1.46	0.01	4907	4.39	0.08	575	1.77	0.02	2.22	0.04	1.39
570	1.48	0.01	4765	4.32	0.07	684	1.84	0.02	2.32	0.04	1.45
580	1.58	0.01	4899	4.82	0.09	588	1.93	0.02	2.45	0.05	1.39
590	1.56	0.01	4495	4.67	0.08	686	1.97	0.02	2.54	0.05	1.53
600	1.66	0.01	4840	5.00	0.09	641	2.05	0.02	2.62	0.05	1.40
610	1.65	0.02	4473	5.04	0.08	678	2.10	0.02	2.72	0.05	1.26
620	1.74	0.01	4712	5.38	0.09	635	2.17	0.02	2.81	0.05	1.44

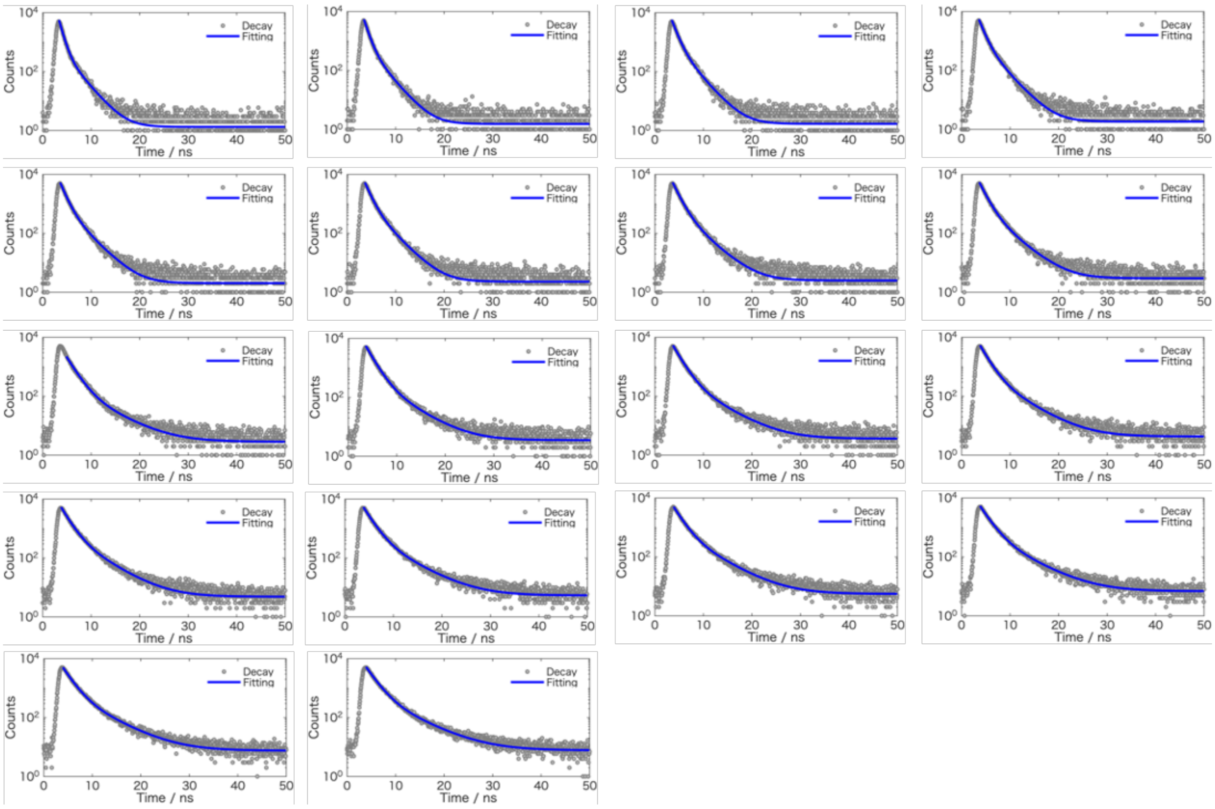

Figure S24. Representative fluorescence decay fitting curves of **DCBS[7]** recorded at 300 K for different emission detection wavelengths (λ_{em}).

To analyze the wavelength-dependence of the emissive behavior, fluorescence lifetimes were measured every 10 nm intervals over 450–620 nm for **DCBS[6]** and 450–630 nm for **DCBS[7]** (**Figure S25**). The two-dimensional wavelength-resolved lifetime maps reveal that both compounds exhibit higher fluorescence intensity and longer lifetimes at longer detection wavelengths. Notably, **DCBS[6]** shows a clear inflection in the lifetime trend around 500 nm. To quantify this behavior, the decay profiles were fitted with a biexponential function,

$$R(t) = B_1 e^{\frac{-t}{\tau_1}} + B_2 e^{\frac{-t}{\tau_2}} \; (S1)$$

where B_i is the amplitude of component i, τ_i is the corresponding lifetime, t is the time after excitation, and $R(t)$ is the fluorescence intensity at time t. From τ_1 and τ_2, the amplitude-weighted average lifetime $\tau_{Av,amp}$ (eq S2) and the intensity-weighted average lifetime $\tau_{Av,int}$ (eq S3), were calculated and plotted as a function of emission wavelength in **Figure S26**:

$$\tau_{Av\_amp} = \frac{\sum_i B_i \tau_i}{\sum_i B_i} \; (S2)$$

$$\tau_{Av\_int} = \frac{\int tR(t)dt}{\int R(t)dt} = \frac{\sum_i B_i \tau_i^2}{\sum_i B_i \tau_i} \; (S3)$$

Here, $\tau_{Av,amp}$ reflects the contribution of each component to the initial emission intensity, whereas $\tau_{Av,int}$ represents the average lifetime weighted by the total number of emitted photons. These averaged lifetimes are therefore suitable parameters for tracking changes in emissive species. For **DCBS[7]**, both $\tau_{Av,amp}$ and $\tau_{Av,int}$ increase monotonically with increasing emission

wavelength, consistent with the coexistence of monomeric and excimer-like species in a largely random and weakly temperature-dependent manner. In contrast, **DCBS[6]** exhibits a pronounced inflection near 500 nm, accompanied by strong temperature dependence, particularly at 80 and 150 K. This behavior indicates stabilization of a specific aggregated species at low temperatures. Based on the single-crystal structure (**Figure 6c**) and the persistent emission band near 540 nm (**Figure 7**), this emissive species is most plausibly assigned to *J*-aggregate-type packing.

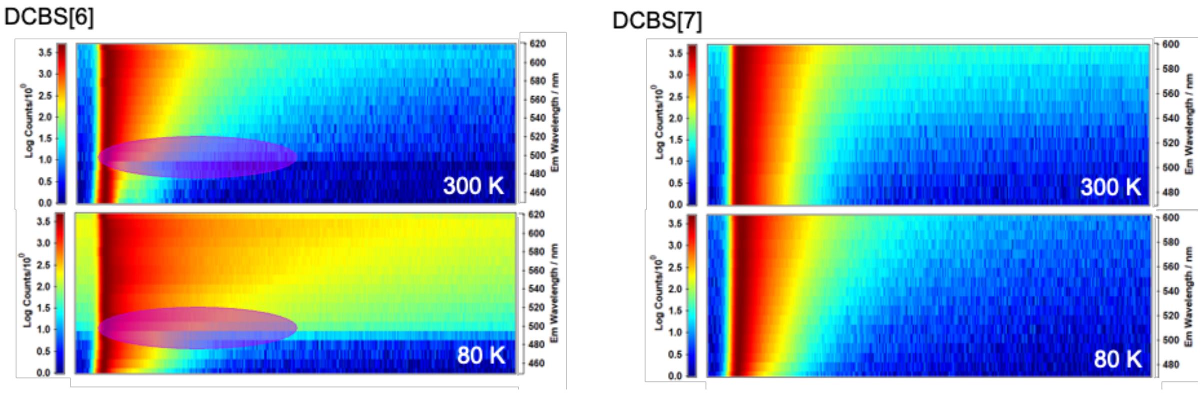

Figure S25. Two-dimensional wavelength-resolved time-resolved fluorescence maps of **DCBS[6]** and **DCBS[7]**, obtained from fluorescence decay profiles at different detection wavelengths. The color scale represents the normalized fluorescence intensity as a function of emission wavelength and delay time after excitation. Data at 300 K (top) and 80 K (bottom) are shown. The excitation wavelength was 402.5 nm.

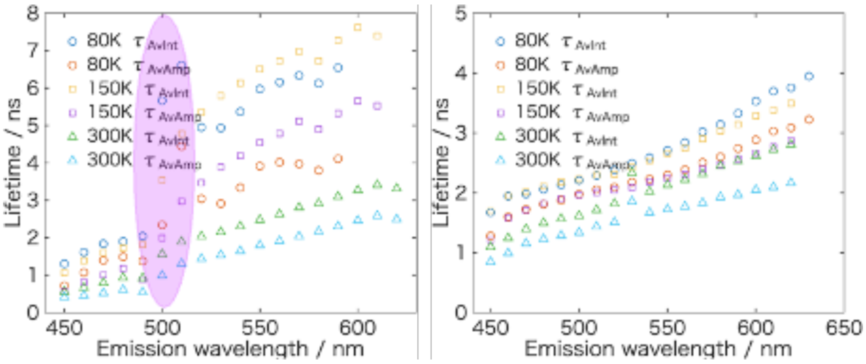

Figure S26. Detection-wavelength-dependent amplitude-averaged lifetime ($\tau_{Av,amp}$) and intensity-averaged lifetime ($\tau_{Av,int}$) extracted from the tail fitting of the data in panel (b). The decay profiles were fitted with a biexponential function according to eq. S1, and $\tau_{Av,amp}$ and $\tau_{Av,int}$ were calculated using eqs. S2 and S3, respectively.

4-2. Femtosecond time-resolve transient absorption spectroscopy

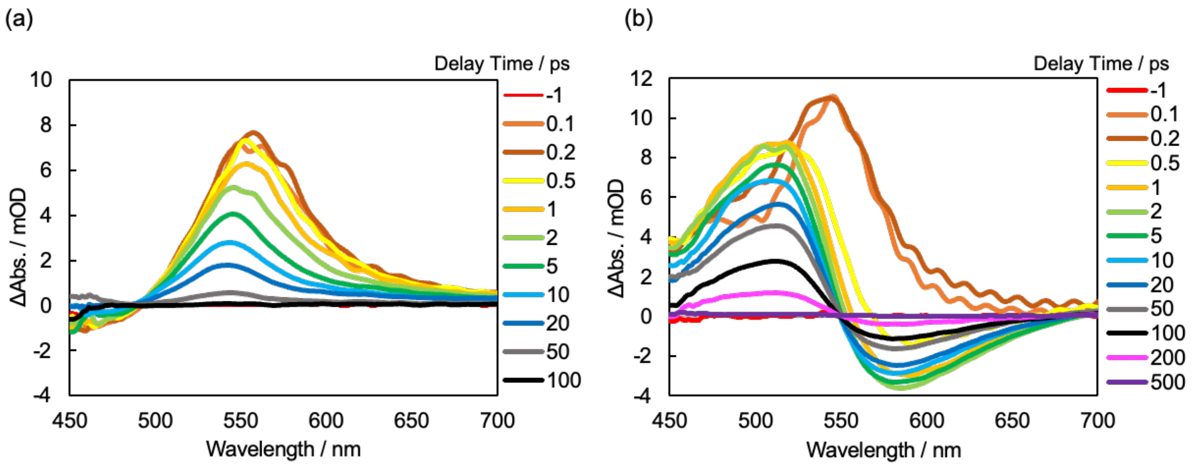

Figure S27. Transient absorption spectra of **DCBS[7]** captured at different time delays after excitation at 400 nm at 293 K in: (a) toluene (τ_1 = 1.8, τ_2 = 21.1 ps) and (b) acetonitrile (τ_1 = 3.2, τ_2 = 49.7 ps).

S5. Crystallographic information

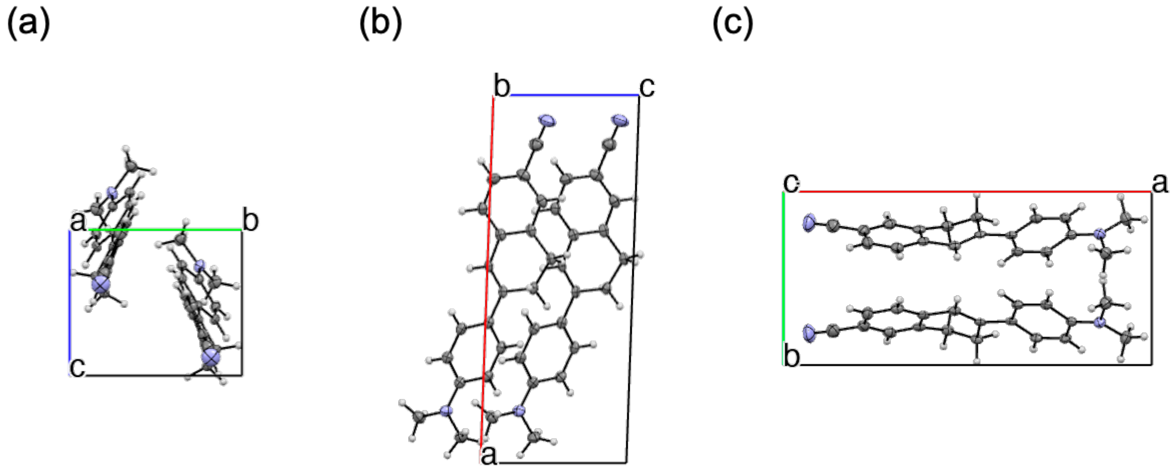

Figure S28. Crystal structure of **DCBS[6]** viewed from different directions. (a) a-axis. (b) b-axis. (c) c-axis. Hydrogen atoms except for those in the disordered groups with low occupancy are omitted for clarity. Thermal ellipsoids are scaled to 50% probability. CCDC2529606

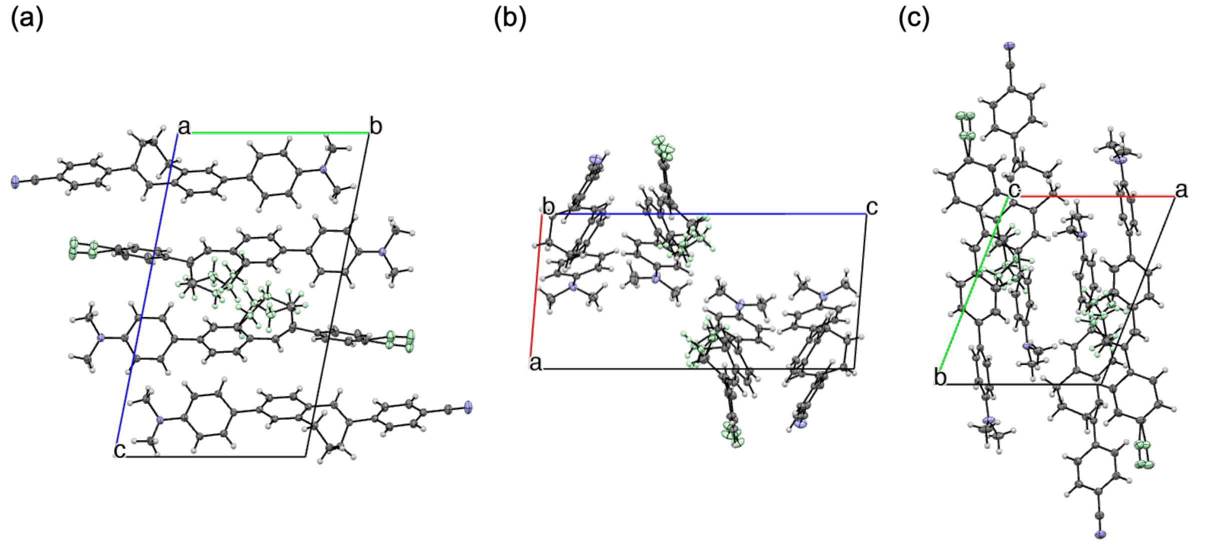

Figure S29. Crystal structure of **DPB[7]N** viewed from different directions. (a) a-axis. (b) b-axis. (c) c-axis. Hydrogen atoms except for those in the disordered groups with low occupancy are omitted for clarity. Thermal ellipsoids are scaled to 50% probability. Due to crystallographic disorder, the data should be regarded as preliminary.

Table S13. Crystallographic data and structure refinement details for **DCBS[6]**, and **DPB[7]N**.

	**DCBS[6]**	**DPB[7]N**
Empirical formula	$C_{19}H_{18}N_2$	$C_{26}H_{24.08}N_2$
Formula weight	274.35	364.55
Tempetature/K	293(2)	293(2)
Space group	Pc	P-1
Crystal system	monoclinic	triclinic
color	yellow	yellow
a/Å	15.8556(11)	9.5093(2)
b/Å	7.4517(5)	11.6771(3)
c/Å	6.2744(4)	18.7778(5)
α/°	90	100.201(2)
b/°	92.011(6)	90.575(2)
γ/°	90	111.079(2)

Volume/Å^3	740.87(9)	1908.65
Z	2	4
ρ_{calc}/g cm^{-3}	1.230	1.269
μ/mm^{-1}	0.073	0.074
F(000)	292.0	776.0
Goodness-of-fit on F^2	1.093	1.057
Final R indexes [I≧2ρ (I)]	R_1 = 0.0674, wR_2 = 0.1770	R_1 = 0.0657, wR_2 = 0.1815
Final R indexes [all data]	R_1 = 0.0742, wR_2 = 0.1809	R_1 = 0.0817, wR_2 = 0.1941
CCDC	2529606	-

S6. 2D Potential Energy Surface (2D-PES)

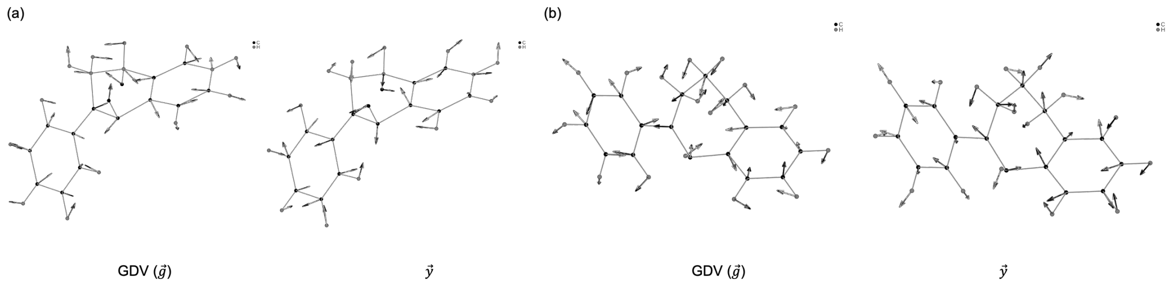

Figure S30. Branching vectors (GDV, $\vec{g}_{IJ}$ and $\vec{y}_{IJ}$) of (a) **BST[6]** and (b) **BST[7]** at MECIs.

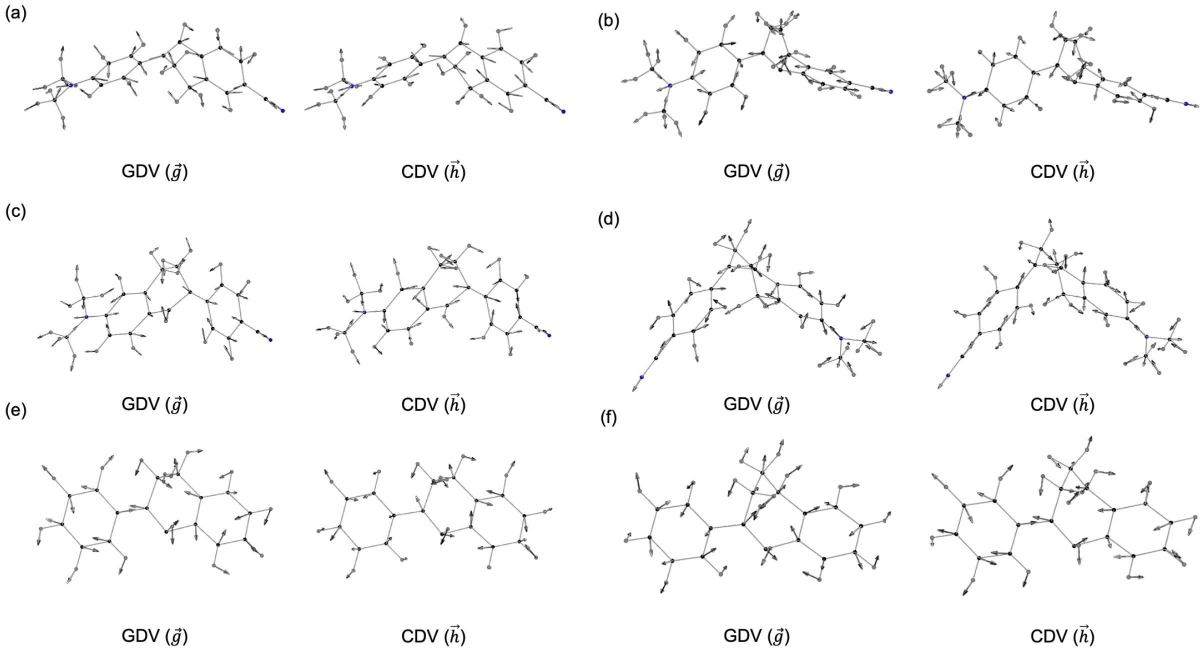

Figure S31. Branching vectors (GDV, $\vec{g}_{IJ}$ and CDV, $\vec{h}_{IJ}$) for (a) **DCBS[6]**, (b) **DCBS[7]**, (c) **DpCBS[6]**, (d) **DpCBS[7]**, (e) **BST[6]**, and (f) **BST[7]** at MECIs.

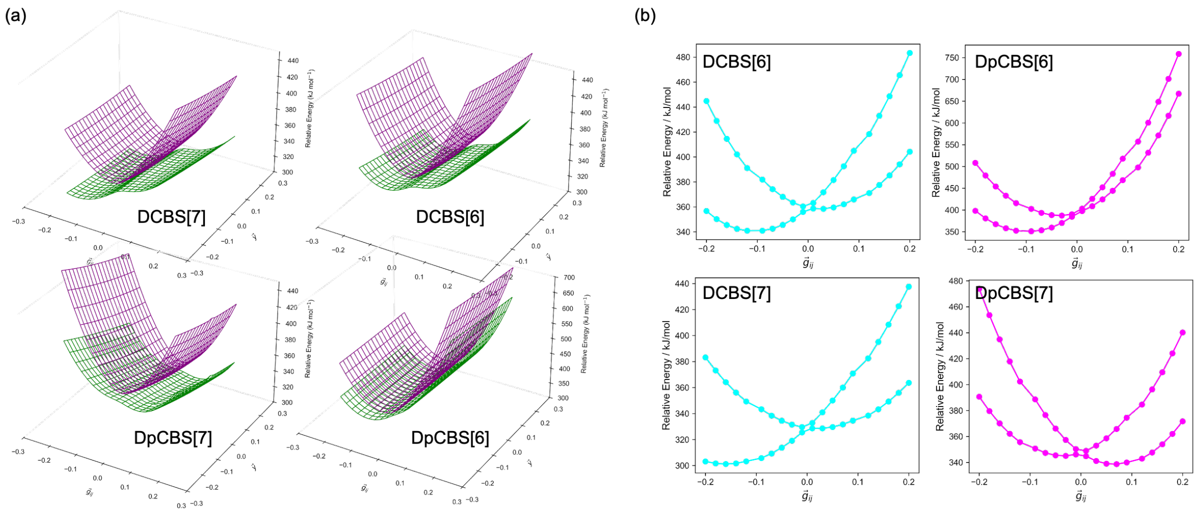

Figure S32. (a) 2D potential energy surfaces (in kJ mol^{-1}) of S_0 and S_1 in the two branching-plane vectors (GDV, $\vec{g}_{IJ}$ and $\vec{y}_{IJ}$)) and (b) One-dimensional PES along GDV, $\vec{g}_{IJ}$ centered at the MECI.

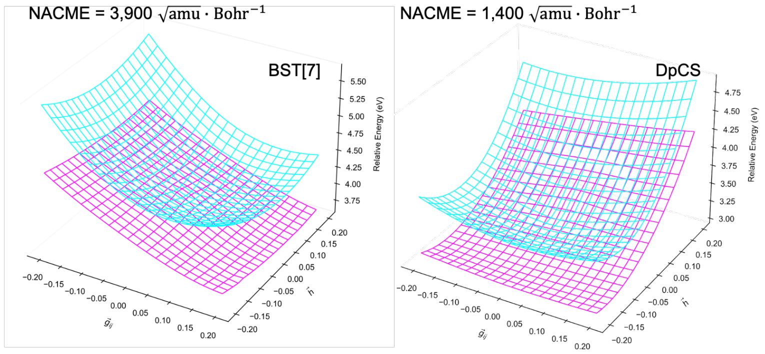

Figure S33. Two-dimensional potential energy surfaces (in eV) of S_0 and S_1 along two branching vectors (GDV, $\vec{g}_{IJ}$ and CDV, $\vec{h}_{IJ}$) with NACME values labeled for **BST[7]** and **DpCS**. NACME of **BST[6]** is 2200.

S7. Synthesis and characterization

7-1. Synthesis of 8-(4-(dimethylamino)phenyl)-6, 7-dihydro-5*H*-benzo[7]annulene-3-carbonitrile (DCBS[7])

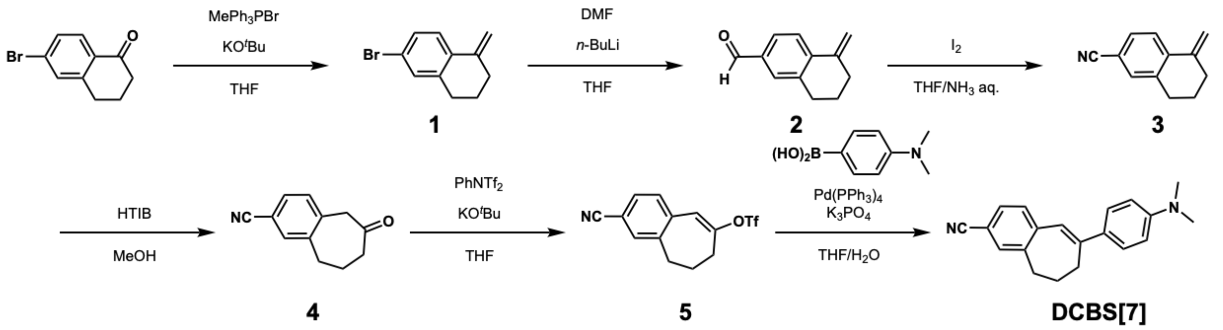

Scheme S1. synthesis of 8-(4-(dimethylamino)phenyl)-6,7-dihydro-5*H*-benzo[7]annulene-3-carbonitrile (**DCBS[7]**).

6-bromo-1-methylene-1,2,3,4-tetrahydronaphthalene (1) Methyl triphenyl phosphonium bromide (1.2 eq, 4.28 g, 12 mmol) was dissolved in THF (30 mL) under an argon atmosphere. The solution was cooled to 0°C, and then KO^{t}Bu (1.5 eq,1.68 g, 15 mmol) was added. Following stirring for 10 min, 6-bromo-1-tetralone (1.0 eq, 2.25 g, 10 mmol) was added and then warmed to room temperature and stirred for 2 h. The reaction mixture was quenched with sat. NH_4Cl aq. and organic products were extracted with dichloromethane. The organic layer was washed with water and brine. The combined organic layers were dried with $MgSO_4$, filtered, and then evaporated under reduced pressure to give a residue. Purification by column chromatography on silica (hexane/dichloromethane = 3:1 (v/v)) gave **6** as a colorless oil. Yield: 95 %; ^{1}H-NMR (399 MHz, $CDCl_3$) δ 7.49 (d, J = 9.1 Hz, 1H, Ar**H**), 7.25 (d, J = 2.3 Hz, 2H, Ar**H**), 5.46 (s, 1H, C=C**H**$_2$), 4.97 (s, 1H, C=C**H**$_2$), 2.81 (t, J = 6.4 Hz, 2H, C**H**$_2$), 2.53 (t, J = 6.2 Hz, 2H, C**H**$_2$), 1.89-1.83 (m, 2H, C**H**$_2$) ppm. (**Figure S34**)

5-methylene-5,6,7,8-tetrahydronaphthalene-2-carbaldehyde (2) 6 (1.0 eq, 1.29 g, 5.42 mmol) was dissolved in dehydrated THF (20 mL) under an argon atmosphere. The reaction mixture was cooled to -78°C, and 2.6 M *n*-BuLi (1.2 eq, 2.50 mL, 6.50 mmol) was added dropwise. The reaction mixture was stirred for 30 min at -78°C and then heated to r.t. DMF (1.5 eq, 0.70 g, 8.13 mmol) was added and stirred for 2 h. After a while, the reaction mixture was quenched with water, extracted with dichloromethane, washed with water and brine, and the combined organic layers were dried with $MgSO_4$, filtered, and then evaporated under reduced pressure to give a residue. Purification by column chromatography on silica (hexane/ethyl acetate = 3:1 (v/v)) gave **7** as a colorless oil. Yield: 92 %; ^{1}H-NMR (500 MHz, $CDCl_3$) δ 9.95 (s, 1H, C**H**O), 7.78 (d, J = 7.9 Hz, 1H, Ar**H**), 7.65 (d, J = 8.2 Hz, 1H, Ar**H**), 7.62 (s, 1H, Ar**H**), 5.63 (s, 1H, C=C**H**$_2$), 5.14 (s, 1H, C=C**H**$_2$), 2.92 (t, J = 6.4 Hz, 2H, C**H**$_2$), 2.58 (t, J = 6.1 Hz, 2H, C**H**$_2$), 1.94-1.89 (m, 2H, C**H**$_2$) ppm. (**Figure S35**)

5-methylene-5,6,7,8-tetrahydronaphthalene-2-carbonitrile (3) A mixture of **7** (1.0 eq, 0.858 g, 4.98 mmol), 30 % NH_3 aq (10 mL), I2 (1.2 eq, 1.51 g, 5.97 mmol) was dissolved in THF (7

mL) and stirred for 3 h. When the reaction mixture turned clear, sat. $Na_2S_2O_3$ aq was added for quenching, extracted with dichloromethane, washed with water and brine, and the combined organic layers were dried with $MgSO_4$, filtered, and then evaporated under reduced pressure to give a residue. Obtained **8** as a colorless oil was used for the next reaction without further purification. Yield: 99 %; ^{1}H-NMR (500 MHz, $CDCl_3$) δ 7.69 (d, J = 8.2 Hz, 1H, Ar**H**), 7.41 (d, J = 7.6 Hz, 2H, Ar**H**), 5.58 (s, 1H, C=C**H**$_2$), 5.13 (s, 1H, C=C**H**$_2$), 2.85 (t, J = 6.3 Hz, 2H, C**H**$_2$), 2.56 (t, J = 6.3 Hz, 2H, C**H**$_2$), 1.88 (m, 2H, C**H**$_2$) ppm. (**Figure S36**)

6-oxo-6,7,8,9-tetrahydro-5*H*-benzo[7]annulene-2-carbonitrile (4) **8** (1.0 eq, 0.834 g, 4.93 mmol) was dissolved in methanol (10 mL), and [hydroxy(tosyloxy)iodo]benzene (HTIB) (1.2 eq, 4.46 g, 11.4 mmol) was added. The solid dissolved rapidly, evolution of heat to a yellow solution. The solution was stirred at room temperature for 2 h, and the solvent was removed in a vacuum. The organic products were extracted with dichloromethane and washed with water and brine. The combined organic layers were dried with $MgSO_4$, filtered, and then evaporated under reduced pressure to give a residue. Purification by column chromatography on silica (hexane/ethyl acetate = 3:1 (v/v)) gave **9** as a colorless solid. Yield: 76 %; ^{1}H-NMR (500 MHz, $CDCl_3$) δ 7.50 (dd, J = 7.8, 1H, Ar**H**), 7.47 (s, 1H, Ar**H**), 7.26 (d, J = 7.6 Hz, 2H, Ar**H**), 3.79 (s, 2H, C**H**$_2$), 3.00 (t, J = 6.4 Hz, 2H, C**H**$_2$), 2.59 (t, J = 6.9 Hz, 2H, C**H**$_2$), 2.05-2.00 (m, 2H, C**H**$_2$) ppm. (**Figure S37**)

3-cyano-6,7-dihydro-5*H*-benzo[7]annulen-8-yl trifluoromethanesulfonate (5) **9** (1.0 eq, 0.693 g, 3.74 mmol) was dissolved in dehydrated THF (15 mL). The mixture was cooled to -20°C under an argon atmosphere. KO^{t}Bu (1.5 eq, 0.631 g, 5.62 mmol) was added to the mixture, warmed to 0°C, stirred for 1 h, and then cooled to -20°C. $PhNTf_2$ (1.2 eq, 1.60 g, 4.48 mmol) was added to the mixture and stirred for 1 h, then warmed to 0°C, and stirred for 4 h. The organic products were extracted with dichloromethane, and the organic layer was washed with water and brine. The combined organic layers were dried with $MgSO_4$, filtered, and then evaporated under reduced pressure to give a residue. Purification by column chromatography on silica (hexane/ethyl acetate = 5:1 (v/v)) gave **10** as a colorless solid: Yield: 90 %; ^{1}H-NMR (500 MHz, $CDCl_3$) δ 7.50 (dd, J = 7.9, 1H, Ar**H**), 7.41 (s, 1H, Ar**H**), 7.28 (d, J = 7.0 Hz, 1H, Ar**H**), 6.59 (d, J = 8.5 Hz, 1H, C=C**H**), 2.91 (t, J = 5.2 Hz, 2H, C**H**$_2$), 2.82 (t, J = 6.3 Hz, 2H, C**H**$_2$), 2.03-2.00 (m, 2H, C**H**$_2$) ppm. (**Figure S38**)

8-(4-(dimethylamino)phenyl)-6,7-dihydro-5*H*-benzo[7]annulene-3-carbonitrile (DCBS[7]) A mixture of **10** (1.0 eq, 0.634 g, 2.0 mmol), 4-(dimethylaminno)phenylboronic acid (1.5 eq, 0.495 g, 3.0 mmol), K_3PO_4 (3.0 eq, 1.45 g, 6.0 mmol) and $Pd(PPh_3)_4$ (5.0 mol%, 0.115 g, 0.10 mmol) was dissolved in 5:1 (v/v) solution of THF and H_2O under argon atmosphere. The reaction mixture was heated to 50°C and stirred for 5 h, and then cooled to r.t. The reaction mixture was quenched with water. The organic products were extracted with dichloromethane, and the organic layer was washed with water and brine. The combined organic layers were dried with $MgSO_4$, filtered, and then evaporated under reduced pressure to give a residue. Purification by column chromatography on silica (hexane/ethyl acetate = 5:1 (v/v)) and recrystallization gave **DCBS[7]** as a yellow solid. Yield; 95 %; ^{1}H-NMR (500 MHz, $CDCl_3$) δ 7.45 (dd, J = 7.9, 1H, Ar**H**), 7.44 (s, 1H, Ar**H**), 7.42 (m, 2H, Ar**H**), 7.25 (d, J = 8.2

Hz, 1H, Ar**H**), 6.74 (d, J = 1.8 Hz, 3H, Ar**H** and C=C**H**), 2.99 (s, 6H, N(C**H**$_3$)), 2.78 (t, J = 6.3 Hz, 2H, C**H**$_2$), 2.64 (t, J = 6.9 Hz, 2H, C**H**$_2$), 2.24-2.19 (m, 2H, C**H**$_2$) ppm. (**Figure S39**) ^{13}C-NMR (100 MHz, CDCl$_3$)) δ 150.4, 146.4, 143.3, 142.0, 132.4, 130.7, 130.5, 129.7, 127.1, 124.0, 119.7, 112.2, 108.8, 40.6, 34.1, 32.2, 30.8 ppm. (**Figure S40**) HRMS (EI+) m/z Calcd. For $C_{20}H_{20}N_2$ $[M]^+$288.1626 Found: 288.3922. FT-IR (KBr): 2927w (νC=CH), 2216m (νCN), 1596s (νC=C), 1520m (νAr-Ar), 1359s (δAr-H) cm^{-1}; mp: 131.5-132.2°C.

7-2. Synthesis of 6-(4-(dimethylamino)phenyl)-7, 8-dihydronaphthalene-2-carbonitrile (DCBS[6])

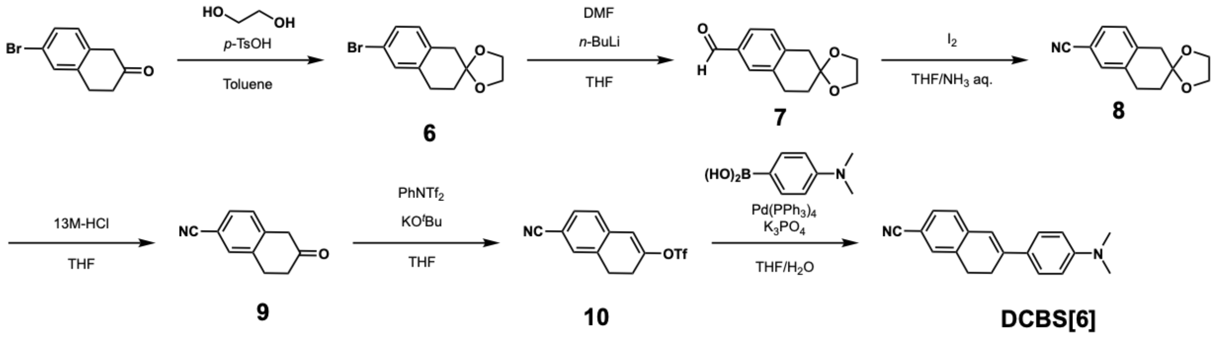

Scheme S2. synthesis of 6-(4-(dimethylamino)phenyl)-7,8-dihydronaphthalene-2-carbonitrile (**DCBS[6]**).

6-bromo-3,4-dihydro-1*H*-spiro[naphthalene-2,2'-[1,3]dioxolane] (6) A mixture of 6-Bromo-2-tetralone (1.0 eq, 5.0 g, 20 mmol), ethylene glycol (10 eq, 12.4 g, 200 mmol), and *p*-TsOH (5.0 mol%, 0.19 g, 1.0 mmol) was dissolved in Toluene (30 mL) equipped with Dean-stark. The mixture was heated to 130 °C and then stirred for 16 h at the same temperature. After cooling to room temperature, $NaHCO_3$ aq. was added to the solution, and organic products were extracted with dichloromethane. The organic layer was washed with water and brine. The combined organic layers were dried with $MgSO_4$, filtered, and then evaporated under reduced pressure to give a residue. Purification by column chromatography on silica (hexane/ethyl acetae = 5:1 (v/v)) gave **1** as a colorless solid. Yield: 97 %; ^{1}H-NMR (500 MHz, CDCl$_3$) δ 7.26 (s, 1H, Ar**H**), 7.23 (d, J = 8.5 Hz, 1H, Ar**H**), 6.91 (d, J = 8.2 Hz, 1H, Ar**H**), 4.02 (m, 4H, C**H**$_2$), 2.96 (t, J = 6.7 Hz, 2H, C**H**$_2$), 2.92 (s, 2H, C**H**$_2$), 1.93 (t, J = 6.7 Hz, 2H, C**H**$_2$) ppm.(**Figure S41**)

3,4-dihydro-1*H*-spiro[naphthalene-2,2'-[1,3]dioxolane]-6-carbaldehyde (7) **1** (1.0 eq, 2.69 g, 10 mmol) was dissolved in dehydrated THF (20 mL) under an argon atmosphere. The reaction mixture was cooled to -78°C, and 2.6 M *n*-BuLi (1.2 eq, 4.61 mL, 12 mmol) was added dropwise. The reaction mixture was stirred for 30 min at -78°C and then heated to r.t. DMF (1.5 eq, 1.00 g, 15 mmol) was added and stirred for 2 h. After a while, the reaction mixture was quenched with water, extracted with dichloromethane, washed with water and brine, and combined organic layers were dried with $MgSO_4$, filtered, and then evaporated under reduced pressure to give a residue. Purification by column chromatography on silica (hexane/ethyl acetate = 3:1 (v/v)) gave **2** as a colorless solid. Yield: 49 %; ^{1}H-NMR (500 MHz, CDCl$_2$) δ 9.94

(s, 1H, CHO), 7.63 (d, J = 8.9 Hz, 2H, Ar**H**), 7.21 (d, J = 7.6 Hz, 1H, Ar**H**), 4.06 (m, 4H, C**H**$_2$), 3.07 (d, J = 6.4 Hz, 4H, C**H**$_2$), 1.99 (t, J = 6.6 Hz, 2H, C**H**$_2$) ppm. (**Figure S42**)

3,4-dihydro-1*H*-spiro[naphthalene-2,2'-[1,3]dioxolane]-6-carbonitrile (**8**) A mixture of **2** (1.0 eq, 0.979 g, 4.49 mmol), 30 % NH_3 aq (10 mL), I_2 (1.2 eq, 1.36 g, 5.38 mmol) was dissolved in THF (7 mL) and stirred for 3 h. When the reaction mixture turned cleared, sat. $Na_2S_2O_3$ aq was added for quenching, extracted with DCM, washed with water and brine and combined organic layers were dried with $MgSO_4$, filtered, and then evaporated under reduced pressure to give a residue. Obtained **3** as a yellow solid was used for the next reaction without further purification. Yield: 95 %; ^{1}H-NMR (500 MHz, $CDCl_3$) δ 7.42 (s, 1H, Ar**H**), 7.39 (d, J = 7.9 Hz, 1H, Ar**H**), 7.14 (d, J = 7.9 Hz, 1H, Ar**H**), 4.04 (m, 4H, C**H**$_2$), 3.01 (t, J = 7.2 Hz, 4H, C**H**$_2$), 1.96 (t, J = 6.7 Hz, 2H, C**H**$_2$) ppm. (**Figure S43**)

6-oxo-5,6,7,8-tetrahydronaphthalene-2-carbonitrile (**9**) **3** (1.0 eq, 0.92 g, 4.27 mmol) was dissolved in THF (10 mL). 13M-HCl (2.4 eq, 0.78 mL, 10.2 mmol) was inserted dropwise into the reaction mixture at r.t. The mixture was stirred for 30 min at the same temperature. After 30 min, a large amount of $NaHCO_3$ aq. was added to quench the reaction. The organic products were extracted with dichloromethane, and the organic layer was washed with water and brine. The combined organic layers were dried with $MgSO_4$, filtered, and then evaporated under reduced pressure to give a residue. Obtained **4** was used for the next reaction without further purification. **4** was given as a colorless solid. Yield: 98 %; ^{1}H-NMR (500 MHz, $CDCl_3$) δ 7.55 (s, 1H, Ar**H**), 7.53 (d, J = 7.6 Hz, 1H, Ar**H**), 7.25 (d, J = 7.9 Hz, 1H, Ar**H**), 3.65 (s, 2H, C**H**$_2$), 3.12 (t, J = 6.6 Hz, 2H, C**H**$_2$), 2.58 (t, J = 6.7 Hz, 2H, C**H**$_2$) ppm. (**Figure S44**)

6-cyano-3,4-dihydronaphthalen-2-yl trifluoromethane sulfonate (**10**) **4** (1.0 eq, 0.73 g, 4.27 mmol) was dissolved in dehydrated THF (15 mL). The mixture was cooled to -20°C under an argon atmosphere. KO^{t}Bu (1.5 eq, 0.716 g, 6.40 mmol) was added to the mixture, warmed to 0°C, stirred for 1 h, and then cooled to -20°C. $PhNTf_2$ (1.2 eq, 1.82 g, 5.12 mmol) was added to the mixture and stirred for 1 h, then warmed to 0°C and stirred for 4 h. The organic products were extracted with dichloromethane, and the organic layer was washed with water and brine. The combined organic layers were dried with $MgSO_4$, filtered, and then evaporated under reduced pressure to give a residue. Purification by column chromatography on silica (hexane/ethyl acetate = 6:1) gave **5** as a colorless oil. Yield: 73 %; ^{1}H-NMR (500 MHz, $CDCl_3$) δ 7.50 (d, J = 7.6 Hz, 1H, Ar**H**), 7.43 (s, 1H, Ar**H**), 7.17 (d, J = 7.9 Hz, 1H, Ar**H**), 6.52 (s, 1H, C=C**H**), 3.10 (t, J = 8.4 Hz, 2H, C**H**$_2$), 2.73 (t, J = 8.4 Hz, 2H, C**H**$_2$) ppm. (**Figure S45**)

6-(4-(dimethylamino)phenyl)-7,8-dihydronaphthalene-2-carbonitrile (**DCBS[6]**) A mixture of **5** (1.0 eq, 0.61 g, 2.0 mmol), 4-(dimethylamino)phenylboronic acid (1.5 eq, 0.495 g, 3.0 mmol), K_3PO_4 (3.0 eq, 1.45 g, 6.0 mmol) and $Pd(PPh_3)_4$ (5.0 mol%, 0.115 g, 0.10 mmol) was dissolved in 5:1 (v/v) solution of THF and water under argon atmosphere. The reaction mixture was heated to 50°C and stirred for 5 h, and then cooled to r.t. The reaction mixture was quenched with water. The organic products were extracted with dichloromethane, and the organic layer was washed with water and brine. The combined organic layers were dried with $MgSO_4$, filtered, and then evaporated under reduced pressure to give a residue. Purification by

column chromatography on silica (hexane/ethyl acetate = 5:1 (v/v)) and recrystallization gave **DCBS[6]** as a yellow solid. Yield: 95 %; ^{1}H-NMR (500 MHz, $CDCl_3$) *δ* 7.49 (d, *J* = 8.2 Hz, 2H, Ar**H**), 7.43 (d, *J* = 7.6 Hz, 1H, Ar**H**), 7.38 (s, 1H, Ar**H**), 7.12 (d, *J* = 7.6 Hz, 1H, Ar**H**), 6.76 (s, 1H, C=C**H**), 6.73 (d, *J* = 7.9 Hz, 2H, Ar**H**), 3.00 (s, 6H, N(C**H**)$_3$), 2.94 (t, *J* = 7.9 Hz, 2H, C**H**$_2$), 2.75 (t, *J* = 7.8 Hz, 2H, C**H**$_2$) ppm. (**Figure S46**) ^{13}C-NMR (100 MHz, $CDCl_3$) *δ* 150.6, 142.9, 140.1, 135.2, 130.8, 130.3, 127.4, 126.4, 126.2, 119.9, 119.4, 112.2, 108.5, 40.5, 27.9, 25.7 ppm. (**Figure S47**) HRMS (EI$^+$) *m/z* Calcd. For $C_{19}H_{18}N_2$ [M]$^+$274.1470 Found: 274.3653. FT-IR (KBr): 1684 (C=O stretch), 1605 (C=C stretch), 1502 (C-H), 820 (Ar-H) cm^{-1}. mp: 190.5-193.0°C.

7-3. Synthesis of 4-(8-(4-(dimethylamino)phenyl)-6,7-dihydro-5*H*-benzo[7]annulen-3-yl)benzonitrile (DPB[7]C) and 4-(3-(4-(dimethylamino)phenyl)-6,7-dihydro-5*H*-benzo[7]annulen-8-yl)benzonitrile (DPB[7]N)

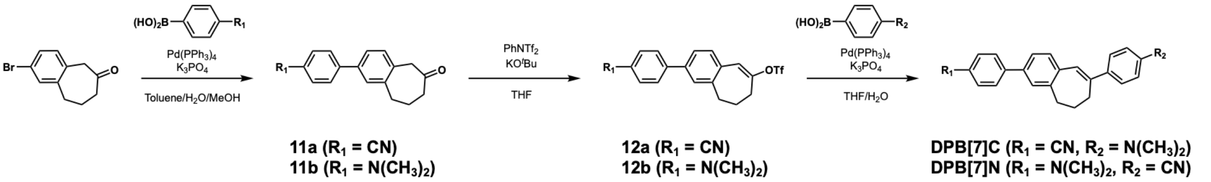

Scheme S3. Synthesis of 4-(8-(4-(dimethylamino)phenyl)-6,7-dihydro-5*H*-benzo[7]annulen-3-yl)benzonitrile (**DPB[7]C**) and 4-(3-(4-(dimethylamino)phenyl)-6,7-dihydro-5*H*-benzo[7]annulen-8-yl)benzonitrile (**DPB[7]N**)

4-(8-(4-(dimethylamino)phenyl)-6,7-dihydro-5*H*-benzo[7]annulen-3-yl)benzonitrile (DPB[7]C)

4-(6-oxo-6,7,8,9-tetrahydro-5*H*-benzo[7]annulen-2-yl)benzonitrile (**11a**) A mixture of 2-bromo-5,7,8,9-tetrahydro-6*H*-benzo[7]annulen-6-one (1.0 eq, 1.2 g, 5.0 mmol), 4-cyanophenyl boronic acid (1.5 eq, 1.1 g, 7.5 mmol), K_3PO_4 (3.0 eq, 3.2 g, 15 mmol) and $Pd(PPh_3)_4$ (5.0 mol%, 0.29 g, 0.15 mmol) was dissolved in 5:2:1 (v/v) solution of toluene, H_2O and MeOH under argon atmosphere. The reaction mixture was heated to 100°C and stirred for 12 h, and then cooled to r.t. The reaction mixture was quenched with water. The organic products were extracted with dichloromethane and the organic layer was washed with water and brine. The combined organic layers were dried with $MgSO_4$, filtered, and then evaporated under reduced pressure to give residue. Purification by column chromatography on silica (hexane/ethyl acetate = 2:1 (v/v)) and recrystallization gave **11a** as a colorless solid. Yield: 65 %; ^{1}H-NMR (500 MHz, $CDCl_3$) δ 7.71 (m, 4H, Ar**H**), 7.44-7.40 (m, 2H, Ar**H**), 7.28 (s, 1H, Ar**H**), 3.80 (s, 2H, C**H**$_2$), 3.05 (t, *J* = 6.4 Hz, 2H, C**H**$_2$), 2.62 (q, *J* = 7.1 Hz, 2H, C**H**$_2$), 2.10-2.03 (m, 2H, C**H**$_2$) ppm. (**Figure S48**)

3-(4-cyanophenyl)-6,7-dihydro-5*H*-benzo[7]annulen-8-yl trifluoromethanesulfonate (**12a**) **11a** (1.0 eq, 0.85 g, 3.25 mmol) was dissolved in dehydrated THF (30 mL). The mixture was cooled to -20°C under an argon atmosphere. KO^{t}Bu (1.5 eq, 0.47 g, 4.2 mmol) was added to the mixture, warmed to 0°C, stirred for 1 h, and then cooled to -20°C. $PhNTf_2$ (1.2 eq, 1.5 g,

4.2 mmol) was added to the mixture and stirred for 1 h, then warmed to 0°C, and stirred for 4 h. The organic products were extracted with dichloromethane, and the organic layer was washed with water and brine. The combined organic layers were dried with $MgSO_4$, filtered, and then evaporated under reduced pressure to give a residue. Purification by column chromatography on silica (hexane/ethyl acetate = 4:1 (v/v)) gave **12a** as a colorless solid: Yield: 99 %; ^{1}H-NMR (500 MHz, $CDCl_3$) δ 7.71 (dd, *J* = 23.8, 8.2 Hz, 8H, Ar**H**), 7.44 (dd, *J* = 7.9, 2.1 Hz, 2H, Ar**H**), 7.35 (s, 2H, Ar**H**), 7.28 (d, *J* = 7.9 Hz, 2H, Ar**H**), 6.63 (s, 2H, C=C**H**), 2.97 (t, *J* = 5.0 Hz, 4H, C**H**$_2$), 2.83 (t, *J* = 6.6 Hz, 4H, C**H**$_2$), 2.07-2.02 (m, 4H, C**H**$_2$) ppm. (**Figure S49**)

4-(8-(4-(dimethylamino)phenyl)-6,7-dihydro-5*H*-benzo[7]annulen-3-yl)benzonitrile (**DPB[7]C**) A mixture of **12a** (1.0 eq, 0.39 g, 1.0 mmol), 4-cyanophenylboronic acid (1.5 eq, 0.18 g, 1.1 mmol), K_3PO_4 (3.0 eq, 0.8 g, 3.0 mmol) and $Pd(PPh_3)_4$ (5.0 mol%, 0.06 g, 0.03 mmol) was dissolved in 5:1 (v/v) solution of THF and H_2O under argon atmosphere. The reaction mixture was heated to 50°C and stirred for 6 h, and then cooled to r.t. The reaction mixture was quenched with water. The organic products were extracted with dichloromethane, and the organic layer was washed with water and brine. The combined organic layers were dried with $MgSO_4$, filtered, and then evaporated under reduced pressure to give a residue. Purification by column chromatography on silica (hexane/ethyl acetate = 5:1 (v/v)) and recrystallization gave **DPB[7]C** as a yellow solid. Yield; 95 %; ^{1}H-NMR (500 MHz, $CDCl_3$) δ 7.72 (s, 4H, Ar**H**), 7.46-7.40 (m, 4H, Ar**H**), 7.30 (d, *J* = 7.8 Hz, 1H, Ar**H**), 6.79 (s, 1H, C=C**H**), 6.75 (d, *J* = 8.2 Hz, 1H, Ar**H**), 2.99 (s, 6H, N(C**H**$_3$)$_2$), 2.87 (t, *J* = 6.2 Hz, 2H, C**H**$_2$), 2.68 (t, *J* = 6.6 Hz, 2H, C**H**$_2$), 2.27-2.21 (m, 2H, C**H**$_2$) ppm. (**Figure S50**) ^{13}C-NMR (100 MHz, $CDCl_3$) δ 155.0, 145.6, 143.9, 141.9, 138.9, 136.4, 132.6, 131.1, 127.8, 127.5, 127.0, 124.9, 124.7, 112.4, 110.5, 40.6, 34.8, 32.5, 30.7 ppm. (**Figure S51**) HRMS (EI^+) m/z Calcd. For $C_{26}H_{24}N_2$ $[M]^+$364.1939, Found: 364.1933.; mp: 265.5-266.2°C.

4-(3-(4-(dimethylamino)phenyl)-6,7-dihydro-5*H*-benzo[7]annulen-8-yl)benzonitrile (DPB[7]N)

2-(4-(dimethylamino)phenyl)-5,7,8,9-tetrahydro-6*H*-benzo[7]annulen-6-one (**11b**) A mixture of 2-bromo-5,7,8,9-tetrahydro-6*H*-benzo[7]annulen-6-one (1.0 eq, 2.9 g, 12 mmol), 4-(dimethylamino)phenyl boronic acid (1.1 eq, 2.4 g, 13 mmol), K_3PO_4 (3.0 eq, 7.6 g, 36 mmol) and $Pd(PPh_3)_4$ (5.0 mol%, 0.69 g, 0.60 mmol) was dissolved in 5:2:1 (v/v) solution of toluene, H_2O and MeOH under argon atmosphere. The reaction mixture was heated to 100°C and stirred for 16 h, and then cooled to r.t. The reaction mixture was quenched with water. The organic products were extracted with dichloromethane and the organic layer was washed with water and brine. The combined organic layers were dried with $MgSO_4$, filtered, and then evaporated under reduced pressure to give residue. Purification by column chromatography on silica (hexane/ethyl acetate = 3:1 (v/v)) and recrystallization gave **11b** as a yellow solid. Yield: 71 %; ^{1}H-NMR (500 MHz, $CDCl_3$) δ 7.50-7.48 (m, 2H, Ar**H**), 7.39-7.36 (m, 2H, Ar**H**), 7.17 (d, *J* = 7.9 Hz, 1H, Ar**H**), 6.80 (d, *J* = 8.2 Hz, 2H, Ar**H**), 3.75 (s, 2H, N(C**H**$_3$)$_2$), 3.02-2.99 (m, 8H, C**H**$_2$), 2.60 (t, *J* = 6.9 Hz, 2H, C**H**$_2$), 2.06-2.01 (m, 2H, C**H**$_2$) ppm. (**Figure S52**)

3-(4-(dimethylamino)phenyl)-6,7-dihydro-5*H*-benzo[7]annulen-8-yl trifluoromethanesulfonate (**12b**) **11b** (1.0 eq, 2.4 g, 8.5 mmol) was dissolved in dehydrated

THF (50 mL). The mixture was cooled to -20°C under an argon atmosphere. KO^{t}Bu (1.5 eq, 1.2 g, 11 mmol) was added to the mixture, warmed to 0°C, stirred for 1 h, and then cooled to -20°C. $PhNTf_2$ (1.2 eq, 3.9 g, 11 mmol) was added to the mixture and stirred for 1 h, then warmed to 0°C, and stirred for 4 h. The organic products were extracted with dichloromethane, and the organic layer was washed with water and brine. The combined organic layers were dried with $MgSO_4$, filtered, and then evaporated under reduced pressure to give a residue. Purification by column chromatography on silica (hexane/dichloromethane = 3:4 (v/v)) gave **12b** as a yellow solid: Yield: 87%; ^{1}H-NMR (500 MHz, $CDCl_3$) δ 7.51 (d, *J* = 8.9 Hz, 2H, Ar**H**), 7.40 (dd, *J* = 7.9, 2.1 Hz, 1H, Ar**H**), 7.31 (d, *J* = 1.5 Hz, 1H, Ar**H**), 7.19 (d, *J* = 7.9 Hz, 1H, Ar**H**), 6.79 (d, *J* = 8.5 Hz, 2H, Ar**H**), 6.60 (s, 1H, C=C**H**), 3.00 (s, 6H, C**H**$_2$), 2.94 (t, *J* = 5.0 Hz, 2H, C**H**$_2$), 2.81 (t, *J* = 6.3 Hz, 2H, C**H**$_2$), 2.05-2.00 (m, 2H, C**H**$_2$) ppm. (**Figure S53**)

4-(3-(4-(dimethylamino)phenyl)-6,7-dihydro-5*H*-benzo[7]annulen-8-yl)benzonitrile (**DPB[7]N**) A mixture of **12b** (1.0 eq, 0.41 g, 1.0 mmol), 4-cyanophenylboronic acid (1.5 eq, 0.22 g, 1.5 mmol), K_3PO_4 (2.0 eq, 0.66 g, 2.0 mmol) and $Pd(PPh_3)_4$ (5.0 mol%, 0.06 g, 0.05 mmol) was dissolved in 5:1 (v/v) solution of THF and H_2O under argon atmosphere. The reaction mixture was heated to 50°C and stirred for 6 h, and then cooled to r.t. The reaction mixture was quenched with water. The organic products were extracted with dichloromethane, and the organic layer was washed with water and brine. The combined organic layers were dried with $MgSO_4$, filtered, and then evaporated under reduced pressure to give a residue. Purification by column chromatography on silica (hexane/ethyl acetate = 5:1 (v/v)) and recrystallization gave **DPB[7]N** as a yellow solid. Yield; 96%; ^{1}H-NMR (500 MHz, $CDCl_3$) δ 7.65 (d, *J* = 8.5 Hz, 2H), 7.60 (d, *J* = 8.2 Hz, 2H, Ar**H**), 7.54 (d, *J* = 8.9 Hz, 2H, Ar**H**), 7.42 (dd, *J* = 7.8, 2.0 Hz, 1H, Ar**H**), 7.39 (s, 1H, Ar**H**), 7.26 (d, *J* = 7.9 Hz, 3H, Ar**H**), 6.88 (s, 1H, C=C**H**), 6.81 (d, *J* = 8.9 Hz, 2H, Ar**H**), 3.01 (s, 6H, C**H**$_2$), 2.90 (t, *J* = 6.1 Hz, 2H, C**H**$_2$), 2.69 (t, *J* = 6.6 Hz, 2H, C**H**$_2$), 2.27-2.22 (m, 2H, C**H**$_2$) ppm. (**Figure S54**) ^{13}C-NMR (100 MHz, $CDCl_3$) δ 150.1, 149.0, 141.8, 140.3, 140.2, 134.2, 132.3, 131.7, 131.4, 128.6, 127.6, 127.0, 126.8, 123.9, 119.2, 112.8, 110.3, 40.6, 35.0, 32.9, 29.8 ppm. (**Figure S55**) HRMS (EI^+) m/z For $C_{26}H_{24}N_2$ $[M]^+$364.1939, Found: 364.1939. ; mp: 268.0-269.2°C.

7-4. Synthesis of 4-(3,4-dihydronaphthalen-2-yl)-*N*,*N*-dimethylaniline (DBS[6]) and 4-(3,4-dihydronaphthalen-2-yl)benzonitrile (CpBS[6])

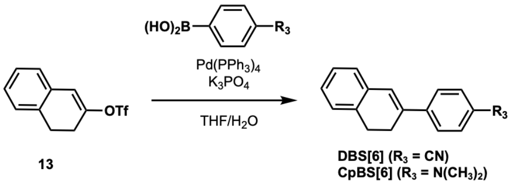

Scheme S4. Synthesis of 4-(3,4-dihydronaphthalen-2-yl)benzonitrile (**CpBS[6]**) and 4-(3,4-dihydronaphthalen-2-yl)-*N*,*N*-dimethylaniline (**DBS[6]**)

4-(3,4-dihydronaphthalen-2-yl)benzonitrile (**CpBS[6]**) A mixture of 3,4-dihydronaphthalen-2-yl trifluoromethanesulfonate (1.0 eq, 0.27 g, 1.0 mmol), 4-cyanophenylboronic acid (1.5 eq,

0.22 g, 1.5 mmol), K_3PO_4 (3.0 eq, 0.64 g, 3.0 mmol) and $Pd(PPh_3)_4$ (5.0 mol%, 0.06 g, 0.05 mmol) was dissolved in 5:1 (v/v) solution of THF and H_2O under argon atmosphere. The reaction mixture was heated to 50°C and stirred for 6 h, and then cooled to r.t. The reaction mixture was quenched with water. The organic products were extracted with dichloromethane, and the organic layer was washed with water and brine. The combined organic layers were dried with $MgSO_4$, filtered, and then evaporated under reduced pressure to give a residue. Purification by column chromatography on silica (hexane/ethyl acetate = 5:1 (v/v)) and recrystallization gave **CpBS[6]** as a yellow solid. Yield; 70%; ^{1}H-NMR (500 MHz, $CDCl_3$) ppm. (**Figure S56**) ^{13}C-NMR (100 MHz, $CDCl_3$) δ 145.7, 136.7, 135.0, 134.0, 132.4, 128.1, 127.5, 127.3, 126.9, 125.6, 119.2, 110.5, 28.0, 26.1 ppm. (**Figure S57**) HRMS (EI^+) m/z Calcd. $C_{17}H_{13}N$ $[M]^+$231.1048, Found: 231.1047. ; mp: 89.5-80.7°C.

4-(3,4-dihydronaphthalen-2-yl)-*N*,*N*-dimethylaniline (**DBS[6]**) A mixture of 3,4-dihydronaphthalen-2-yl trifluoromethanesulfonate (1.0 eq, 0.27 g, 1.0 mmol), 4-(dimethyamino)phenylboronic acid (1.5 eq, 0.25 g, 1.5 mmol), K_3PO_4 (3.0 eq, 0.63 g, 3.0 mmol) and $Pd(PPh_3)_4$ (5.0 mol%, 0.06 g, 0.05 mmol) was dissolved in 5:1 (v/v) solution of THF and H_2O under argon atmosphere. The reaction mixture was heated to 50°C and stirred for 6 h, and then cooled to r.t. The reaction mixture was quenched with water. The organic products were extracted with dichloromethane, and the organic layer was washed with water and brine. The combined organic layers were dried with $MgSO_4$, filtered, and then evaporated under reduced pressure to give a residue. Purification by column chromatography on silica (hexane/ethyl acetate = 5:1 (v/v)) and recrystallization gave **DBS[6]** as a yellow solid. Yield; 85%; ^{1}H-NMR (500 MHz, $CDCl_3$) δ 7.48 (dt, *J* = 9.7, 2.6 Hz, 2H, **ArH**), 7.18-7.09 (m, 4H, Ar**H**), 6.75-6.80 (2H, Ar**H**), 6.74 (s, 1H, C=C**H**), 2.99 (s, 6H, N(C**H**$_3$)$_2$), 2.94 (t, *J* = 7.9 Hz, 2H, C**H**$_2$), 2.74-2.71 (m, 2H, C**H**$_2$)ppm. (**Figure S58**) ^{13}C-NMR (100 MHz, $CDCl_3$) δ 150.1, 138.7, 135.5, 134.6, 129.0, 127.2, 126.6, 126.2, 126.1, 121.0, 112.4, 40.6, 28.4, 26.2 ppm. (**Figure S59**) HRMS (EI^+) m/z Calcd. For $C_{18}H_{19}N_2$ $[M]^+$249.1517, Found: 249.1516.; mp: 124.8-125.6°C.

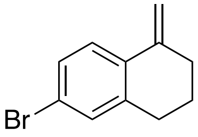

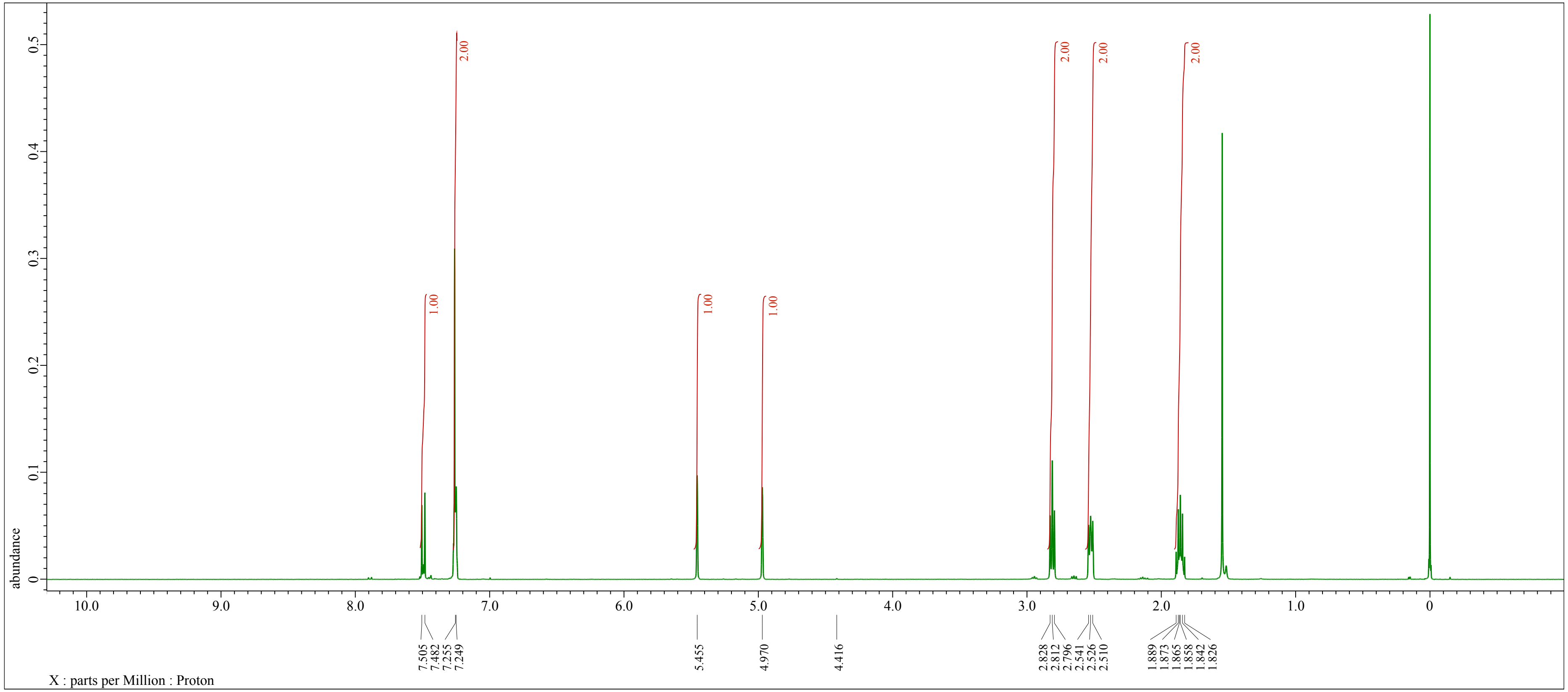

Figure S34. ^{1}H-NMR spectrum of **6** (399 MHz, $CDCl_3$).

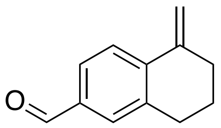

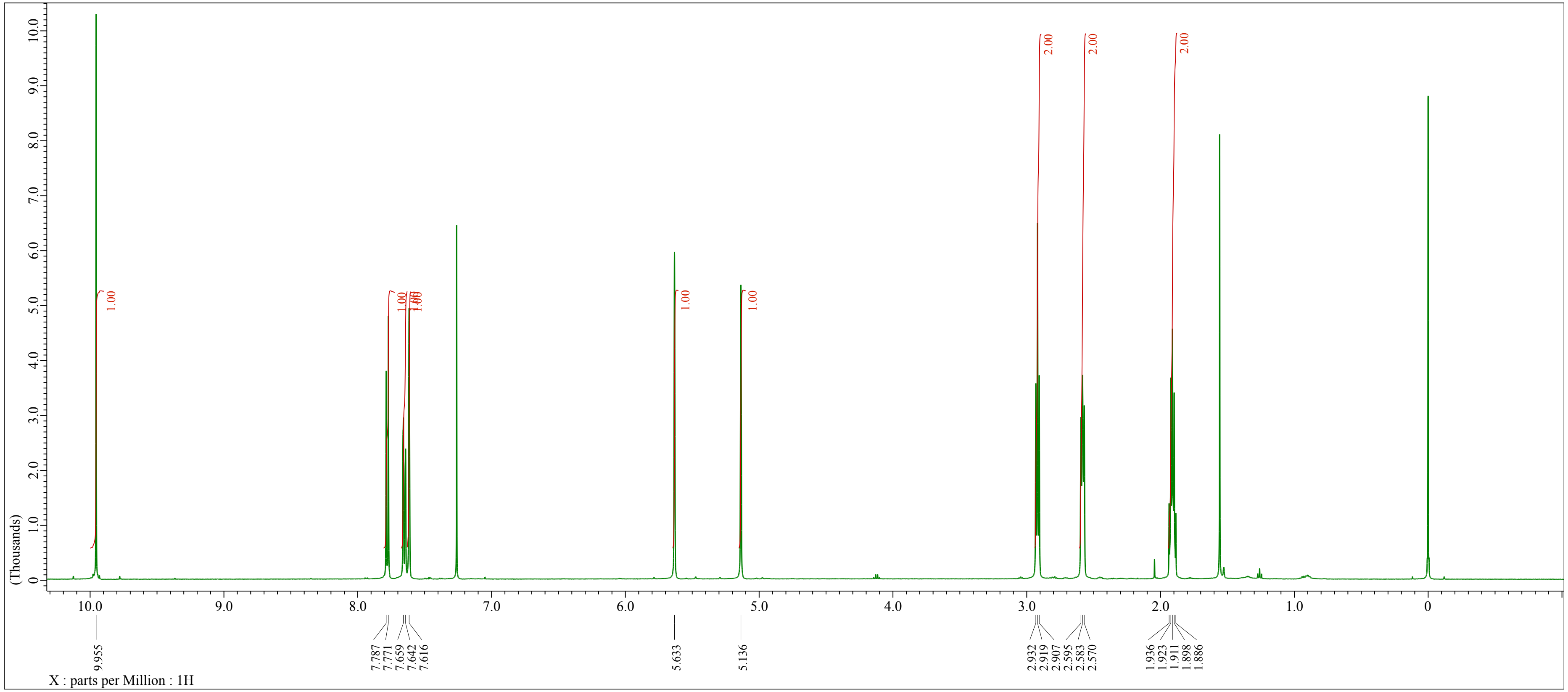

Figure S35. ^{1}H-NMR spectrum of **7** (500 MHz, $CDCl_3$).

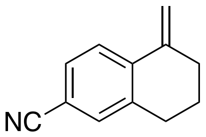

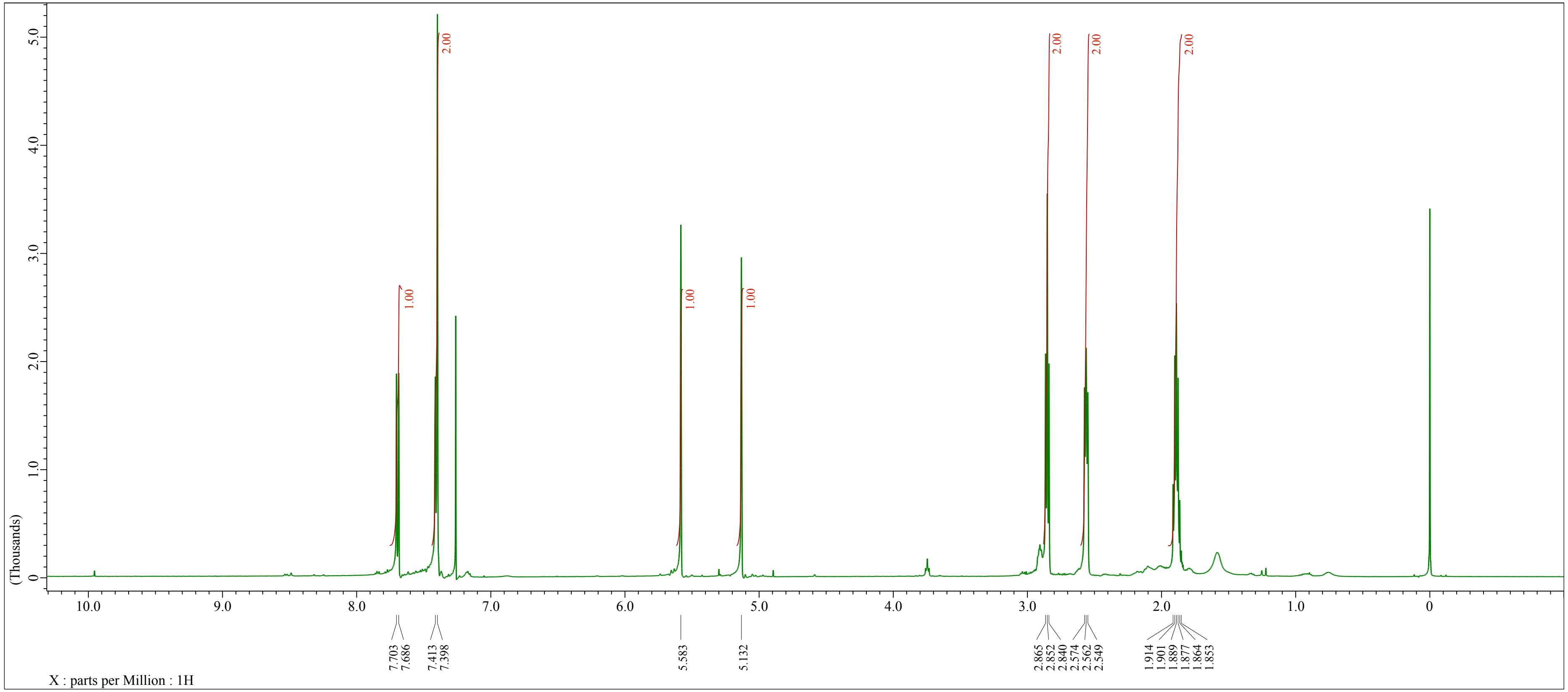

Figure S36. ^{1}H-NMR spectrum of **8** (500 MHz, $CDCl_3$).

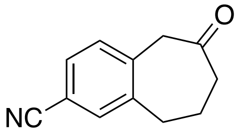

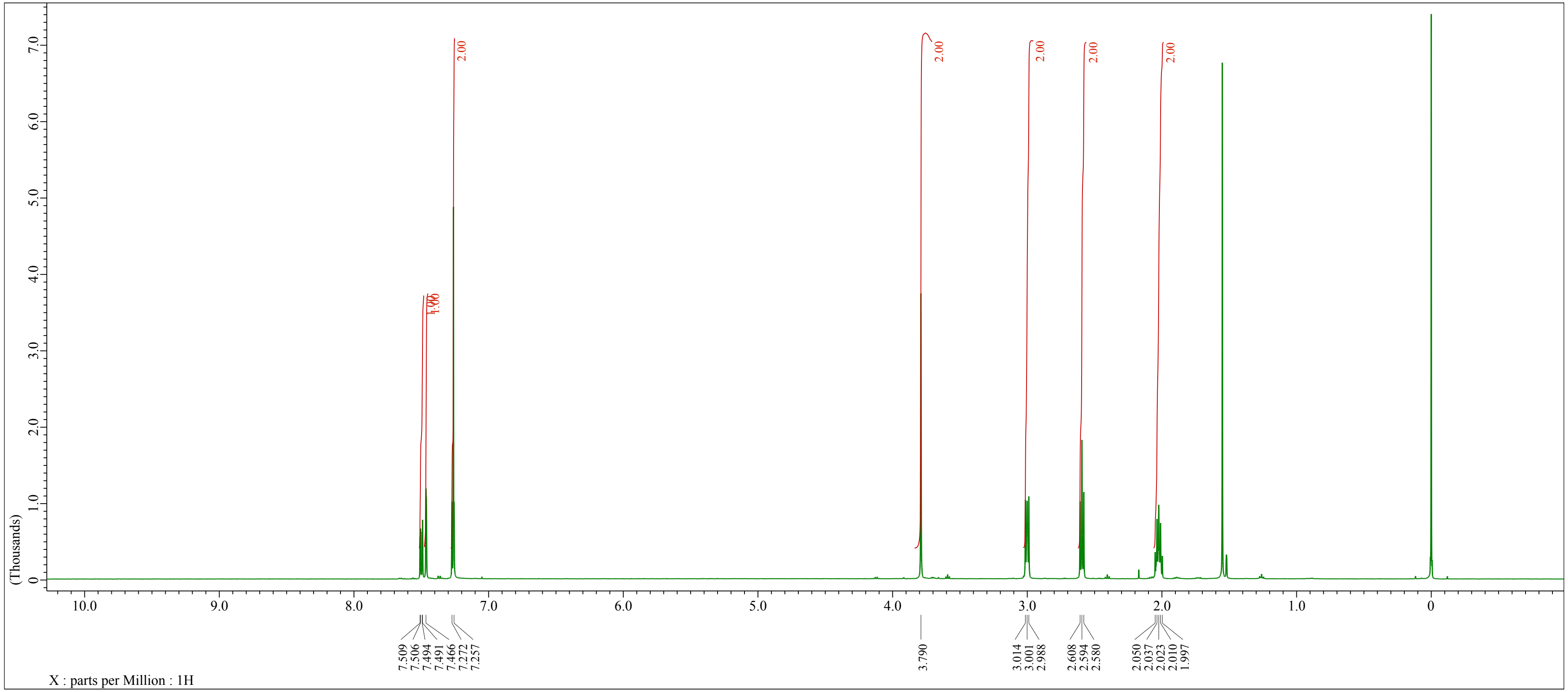

Figure S37. ^{1}H-NMR spectrum of **9** (500 MHz, $CDCl_3$).

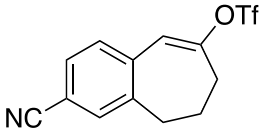

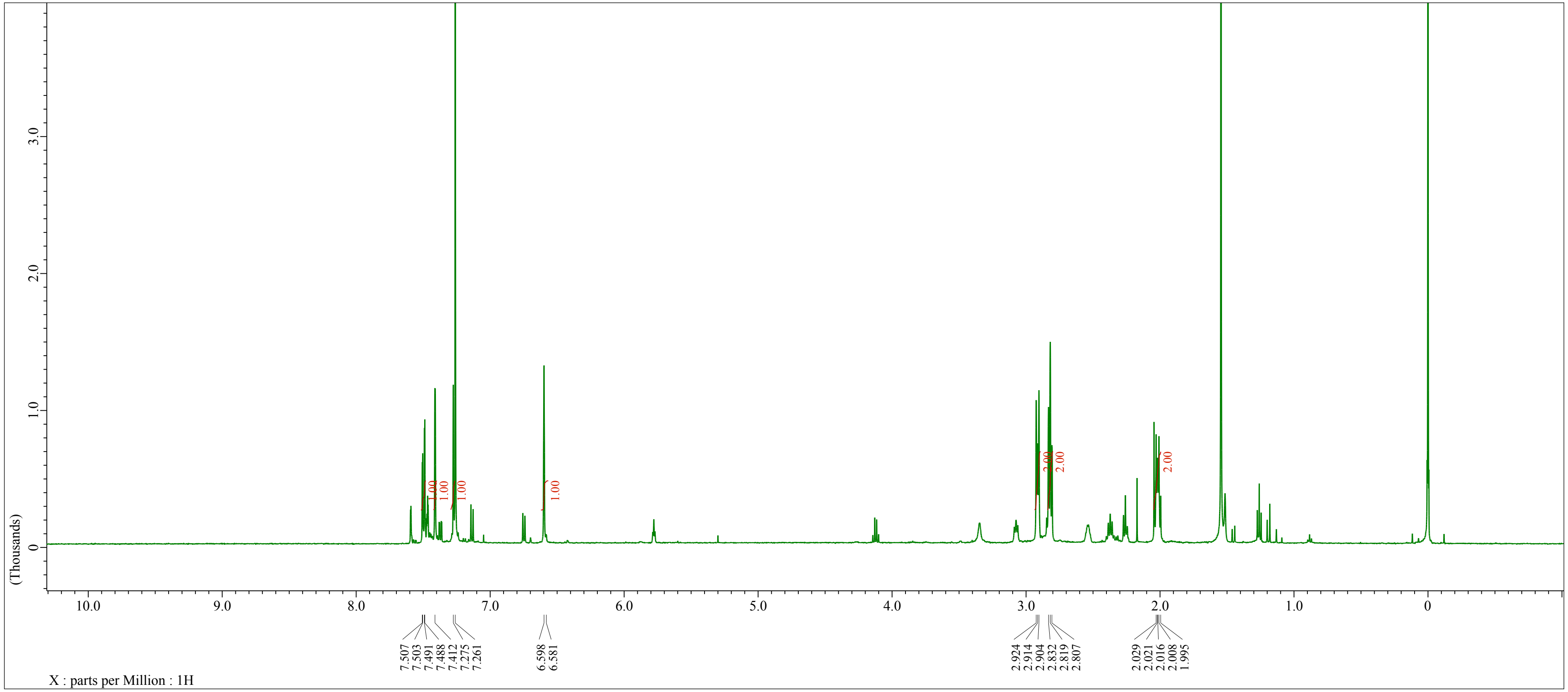

Figure S38. ^{1}H-NMR spectrum of **10** (500 MHz, $CDCl_3$).

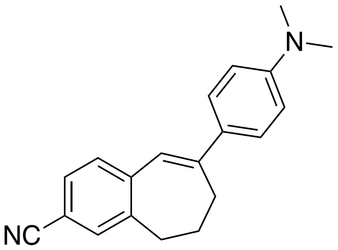

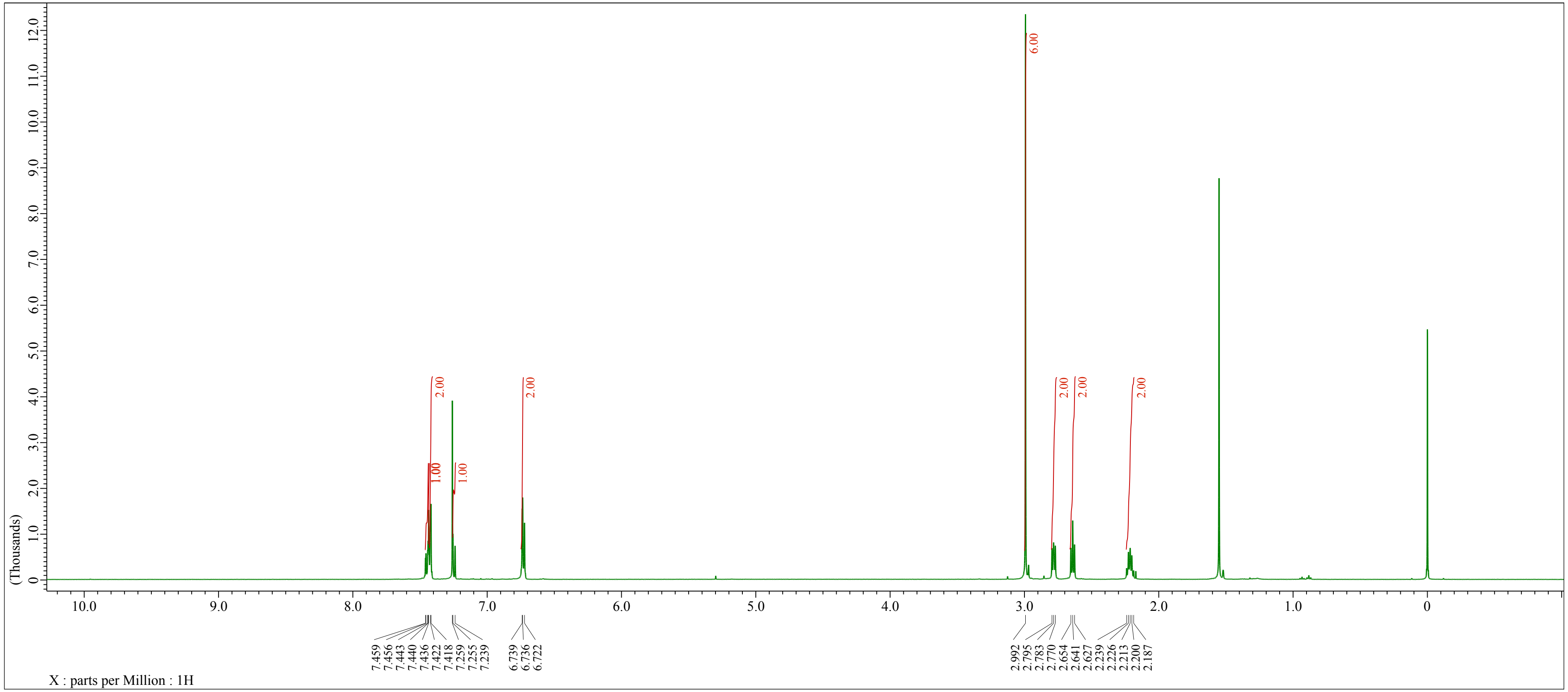

Figure S39. ^{1}H-NMR spectrum of **DCBS[7]** (500 MHz, $CDCl_3$).

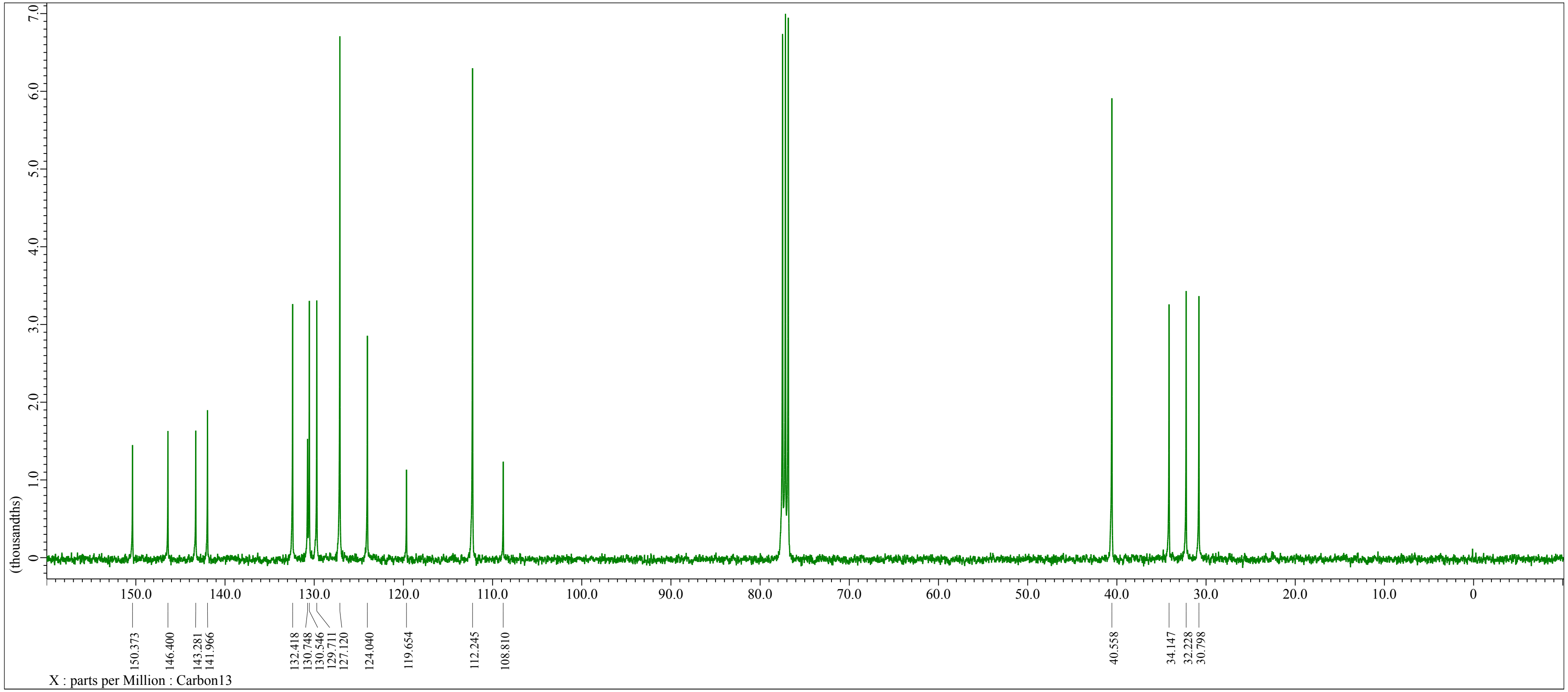

Figure S40. ^{13}C-NMR spectrum of **DCBS[7]** (100 MHz, $CDCl_3$).

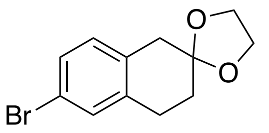

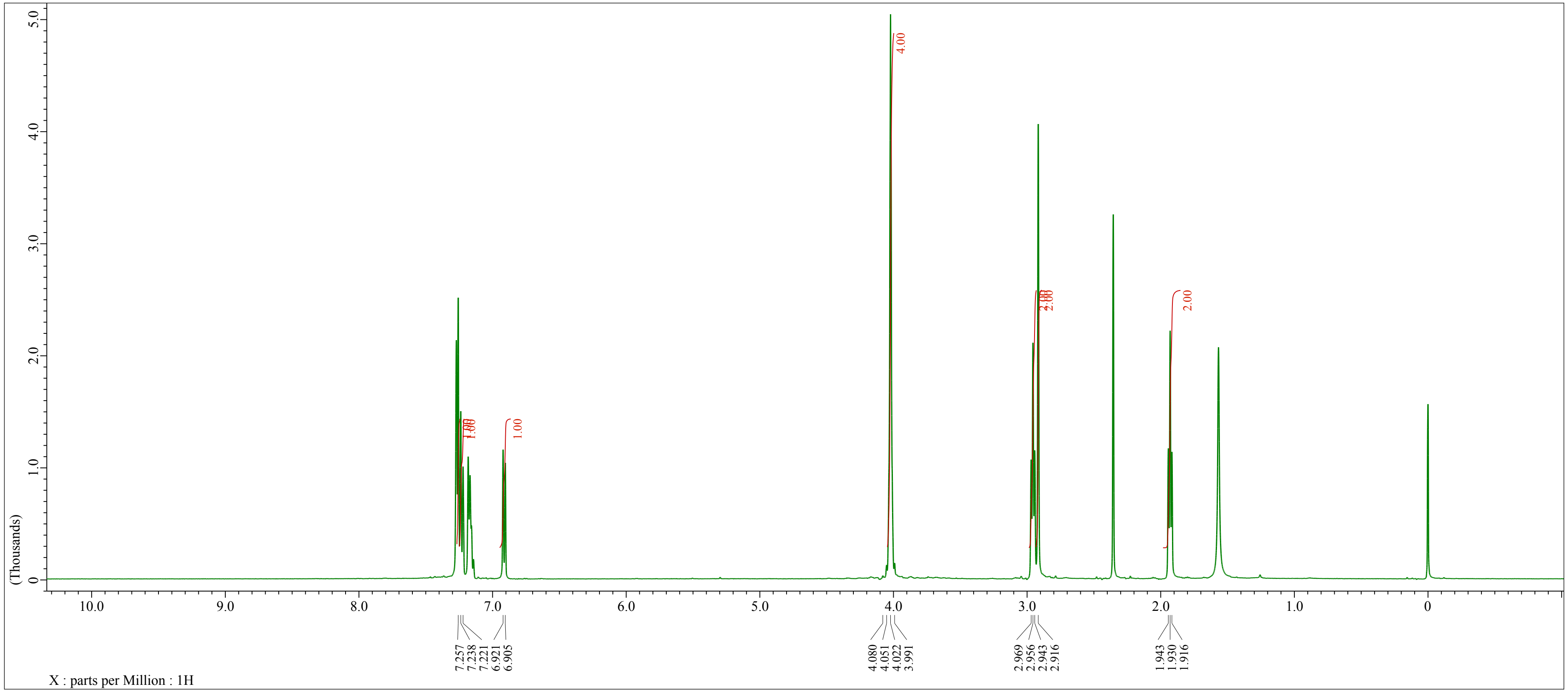

Figure S41. ^{1}H-NMR spectrum of **1** (500 MHz, $CDCl_3$).

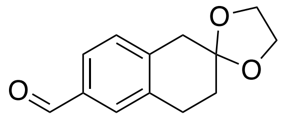

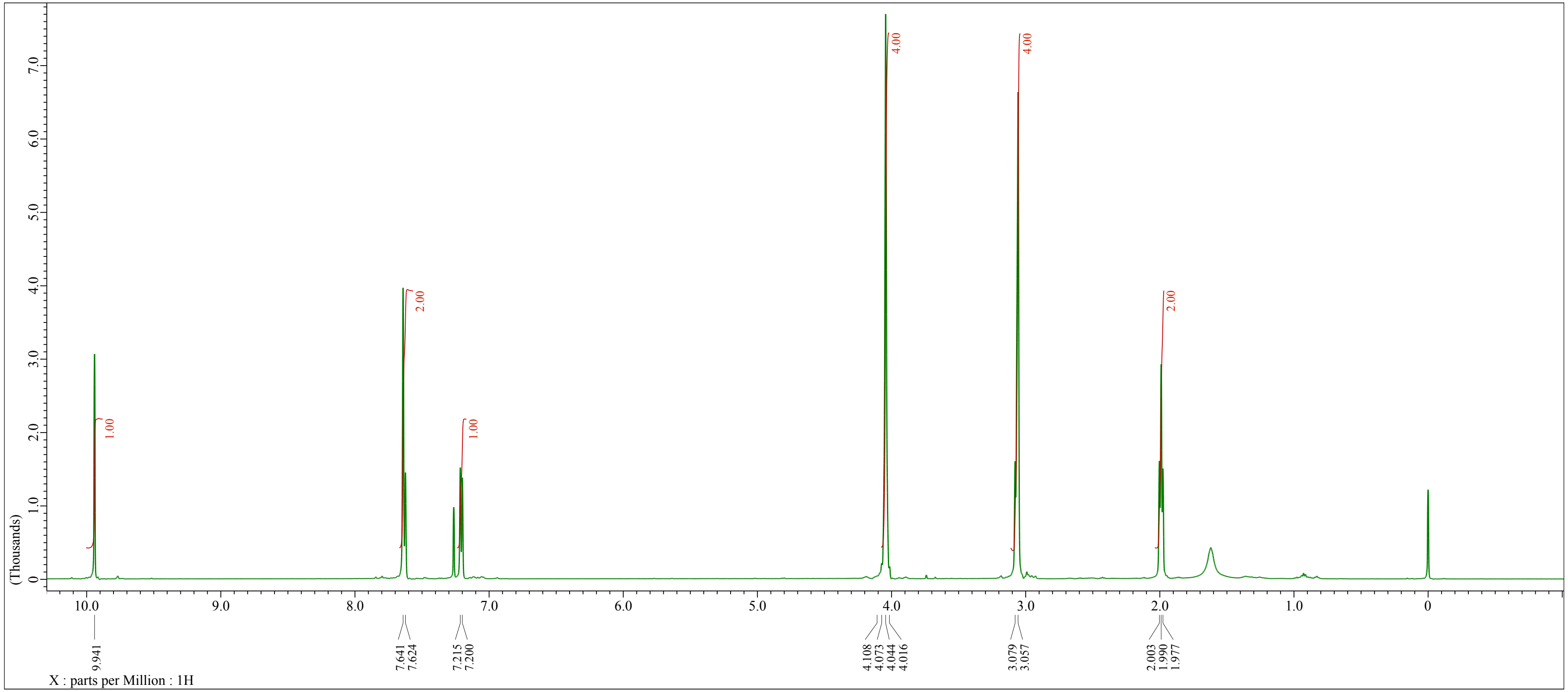

Figure S42. ^{1}H-NMR spectrum of **2** (500 MHz, $CDCl_3$).

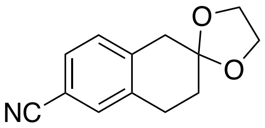

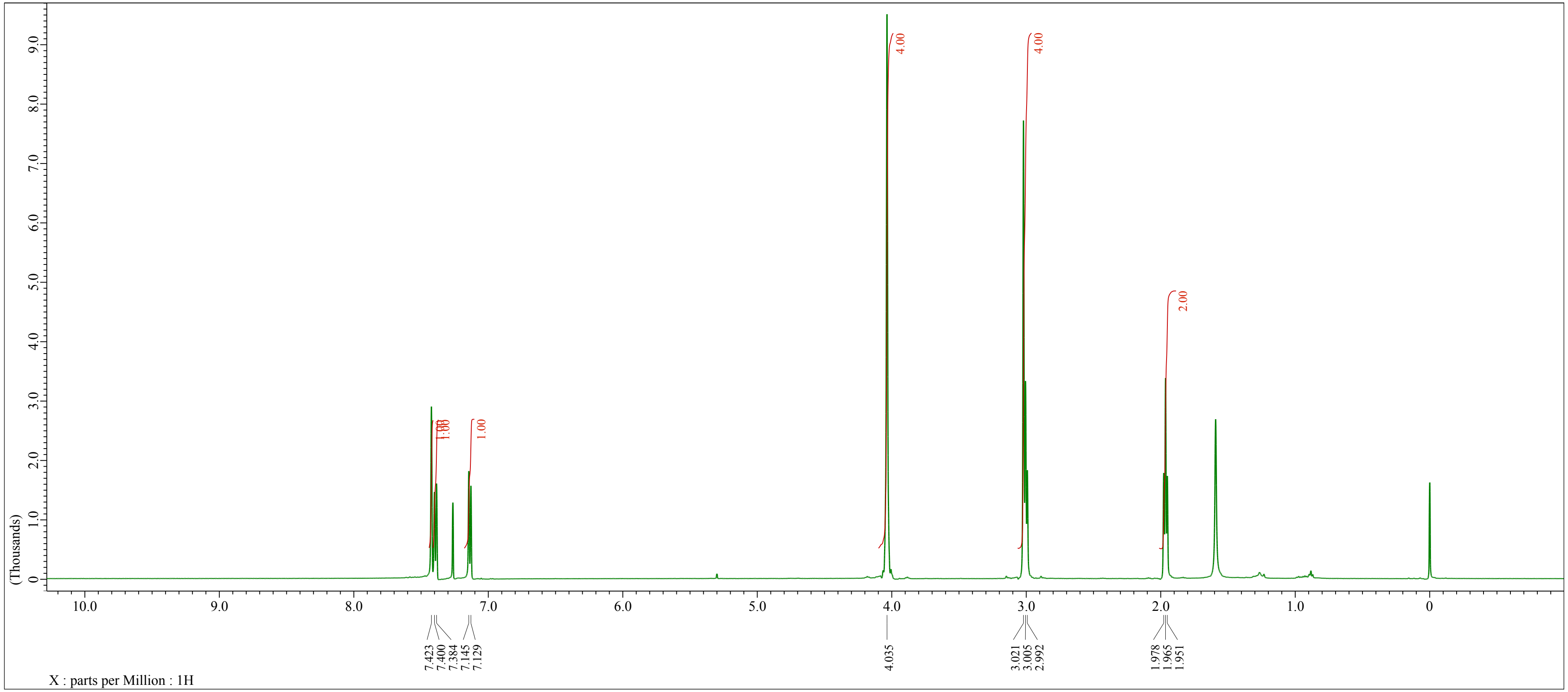

Figure S43. ^{1}H-NMR spectrum of **3** (500 MHz, $CDCl_3$).

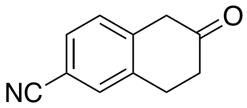

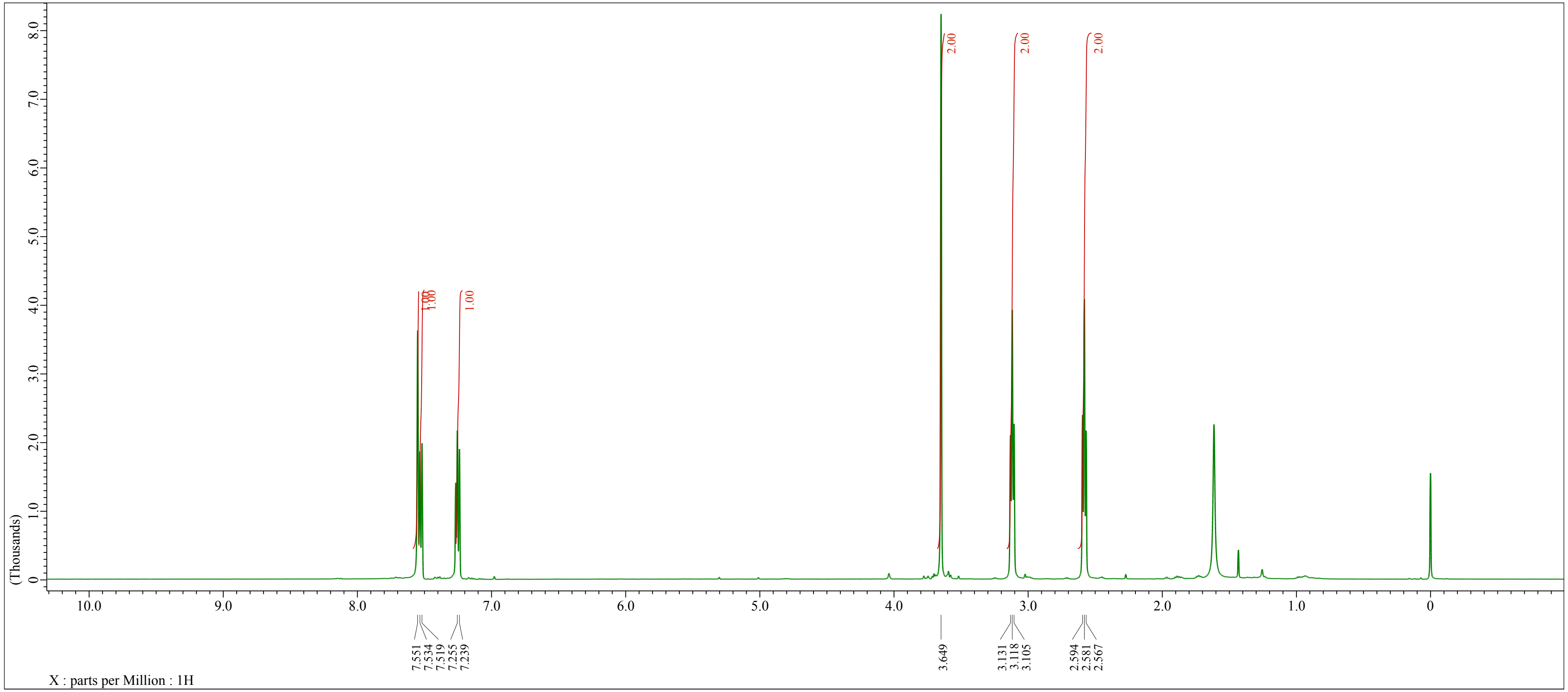

Figure S44. ^{1}H-NMR spectrum of **4** (500 MHz, $CDCl_3$).

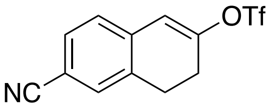

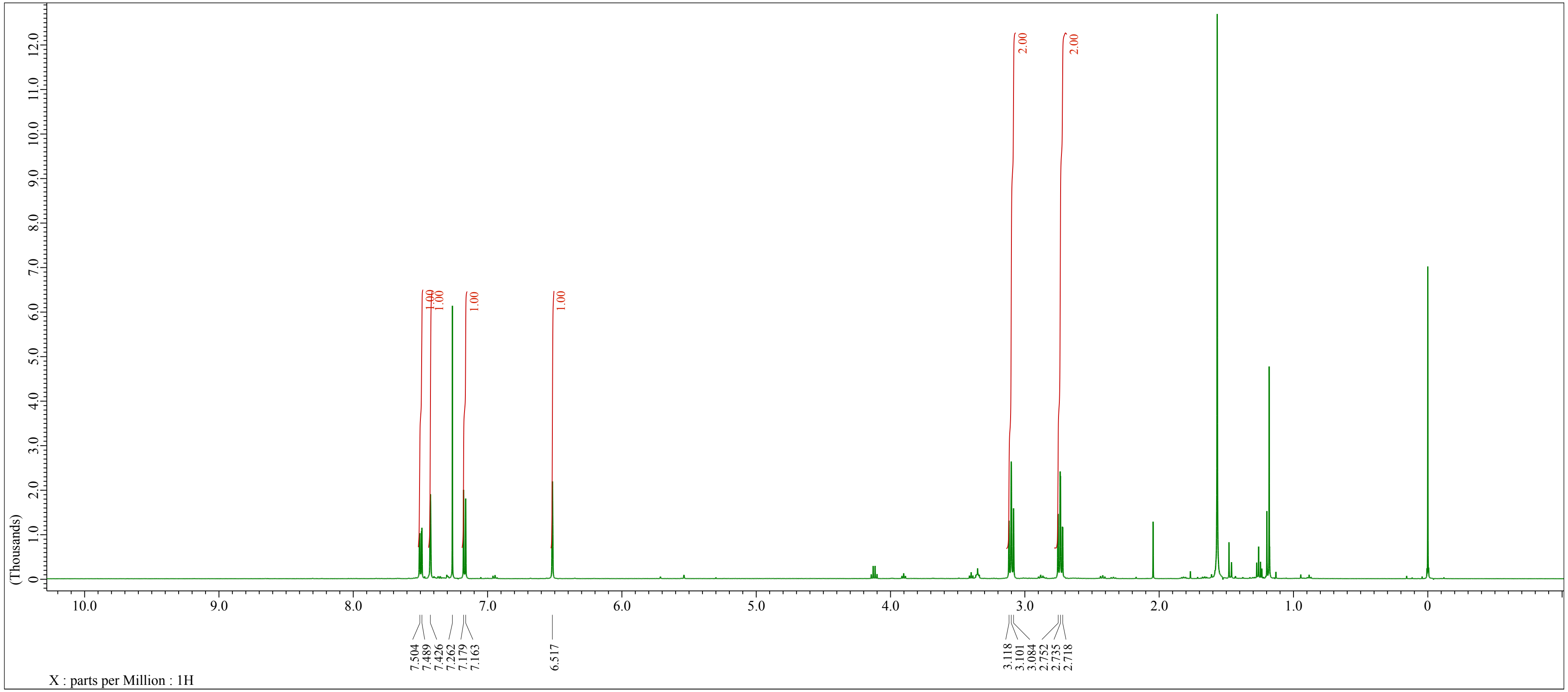

Figure S45. ^{1}H-NMR spectrum of **5** (500 MHz, $CDCl_3$).

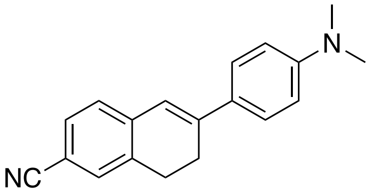

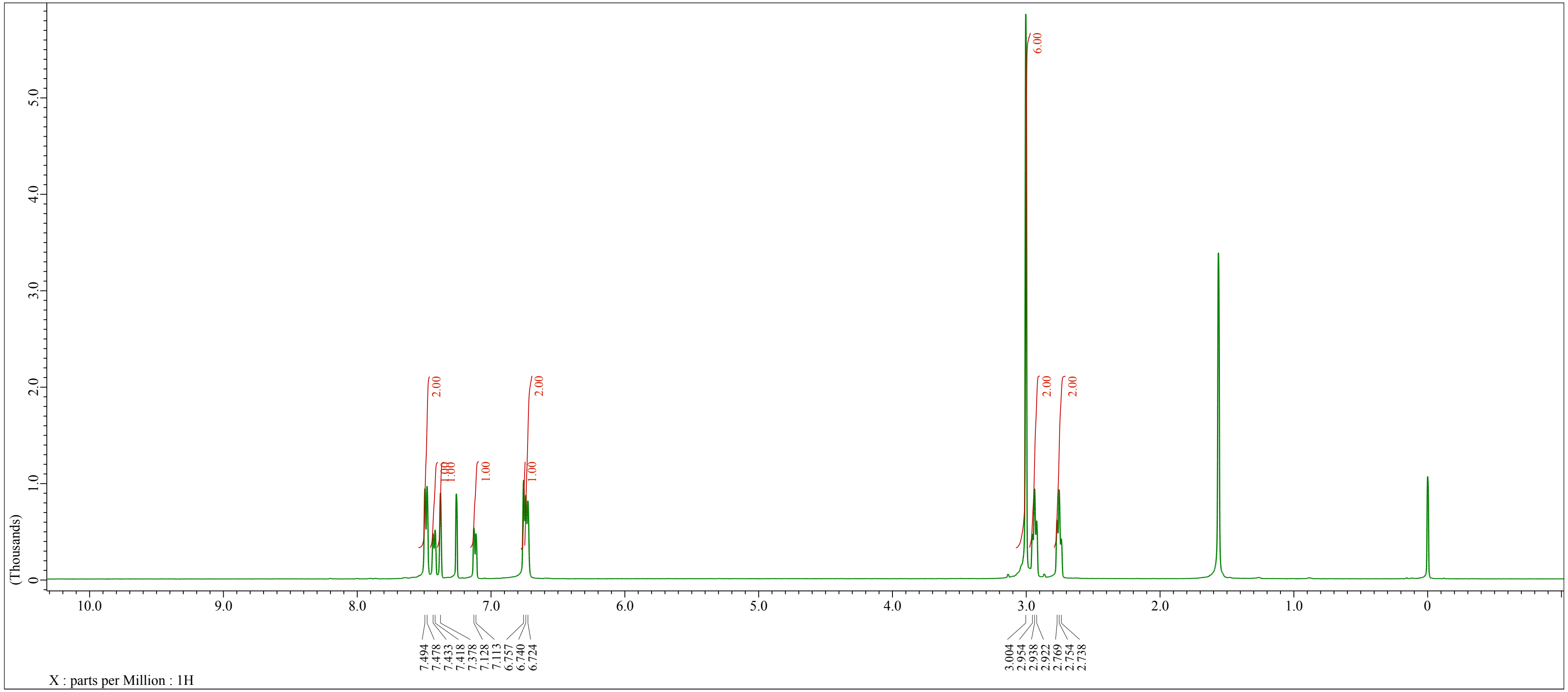

Figure S46. ^{1}H-NMR spectrum of **DCBS[6]** (500 MHz, $CDCl_3$).

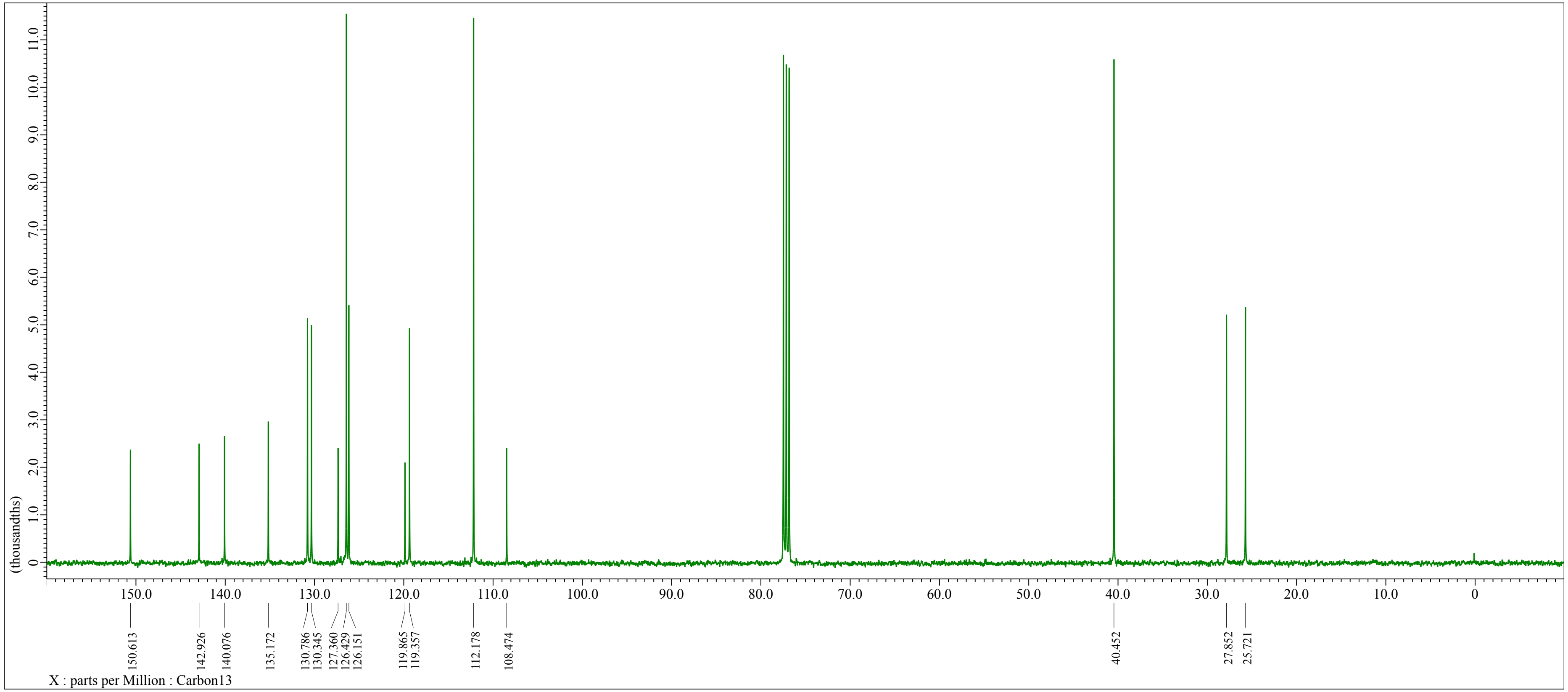

Figure S47. ^{13}C-NMR spectrum of **DCBS[6]** (100 MHz, $CDCl_3$).

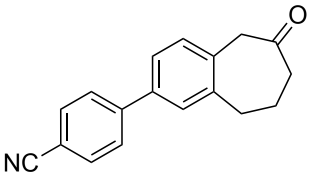

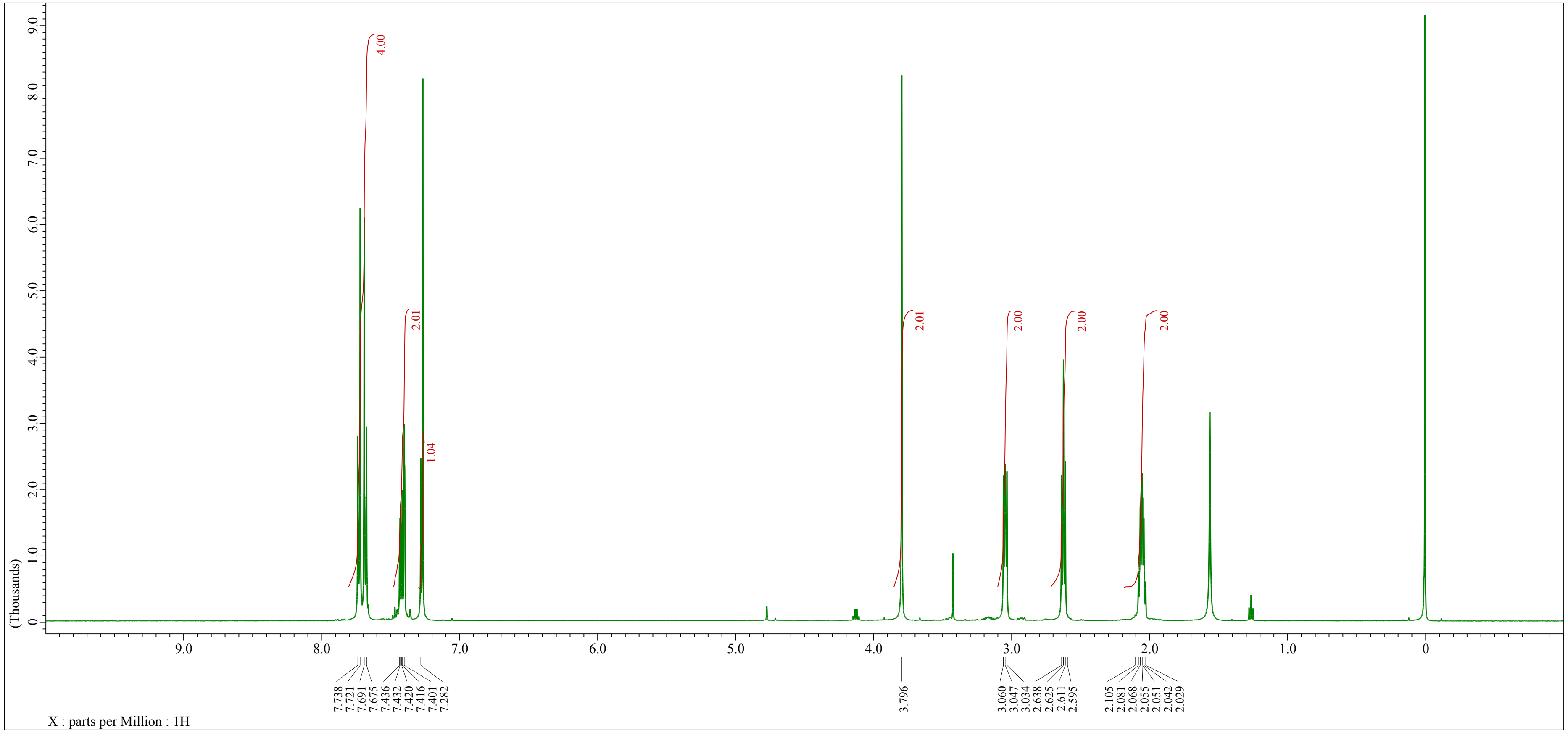

Figure S48. ^{1}H-NMR spectrum of **11a** (500 MHz, $CDCl_3$).

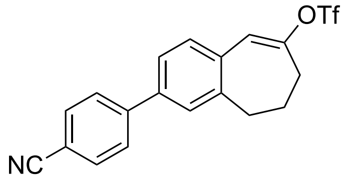

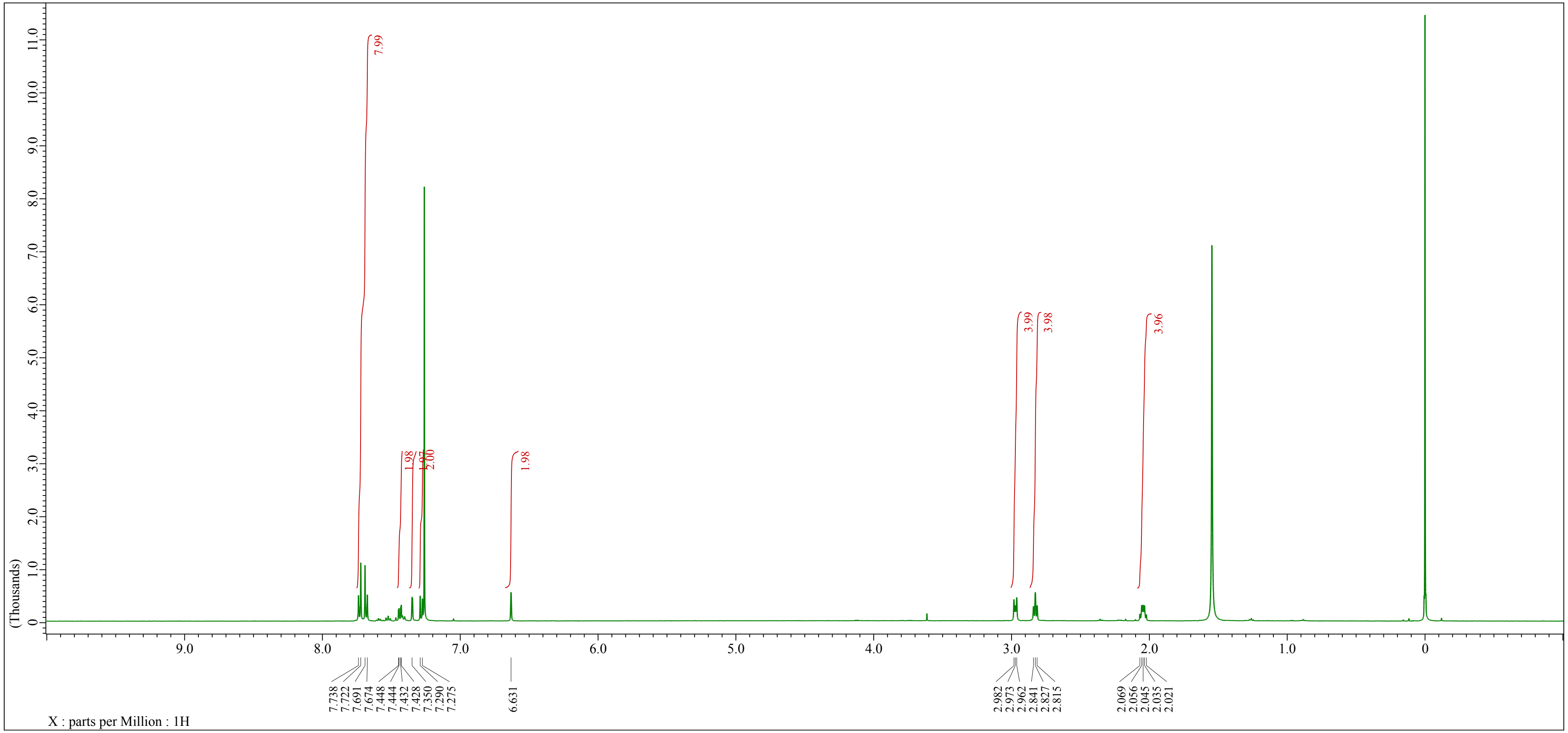

Figure S49. ^{1}H-NMR spectrum of **12a** (500 MHz, $CDCl_3$).

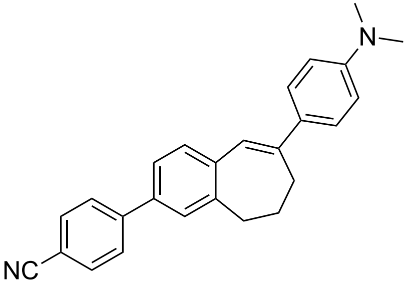

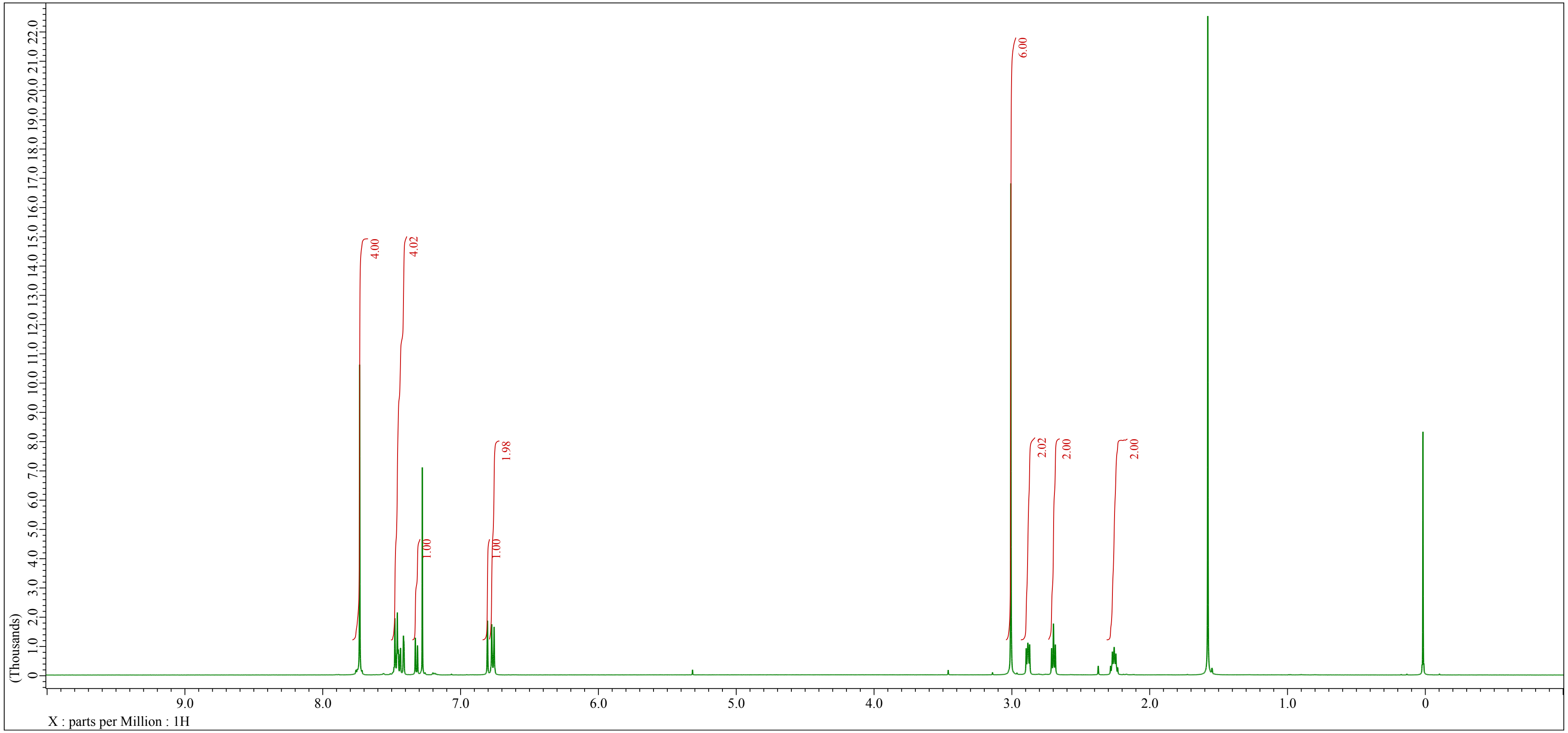

Figure S50. ^{1}H-NMR spectrum of **DPB[7]C** (500 MHz, $CDCl_3$).

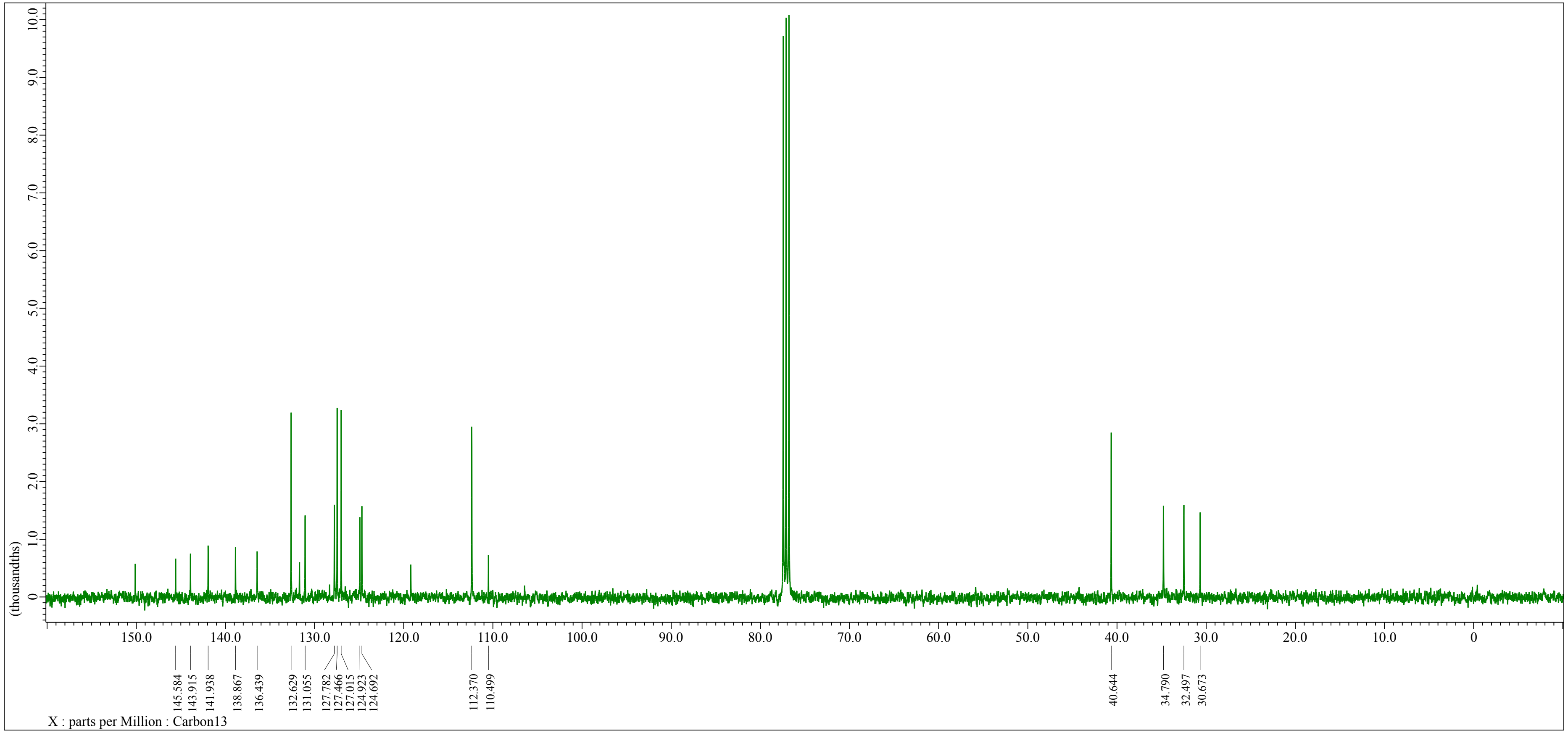

Figure S51. ^{13}C-NMR spectrum of **DPB[7]C** (100 MHz, $CDCl_3$).

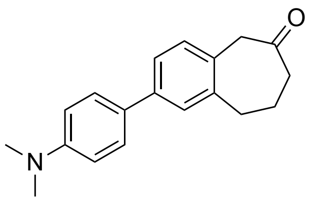

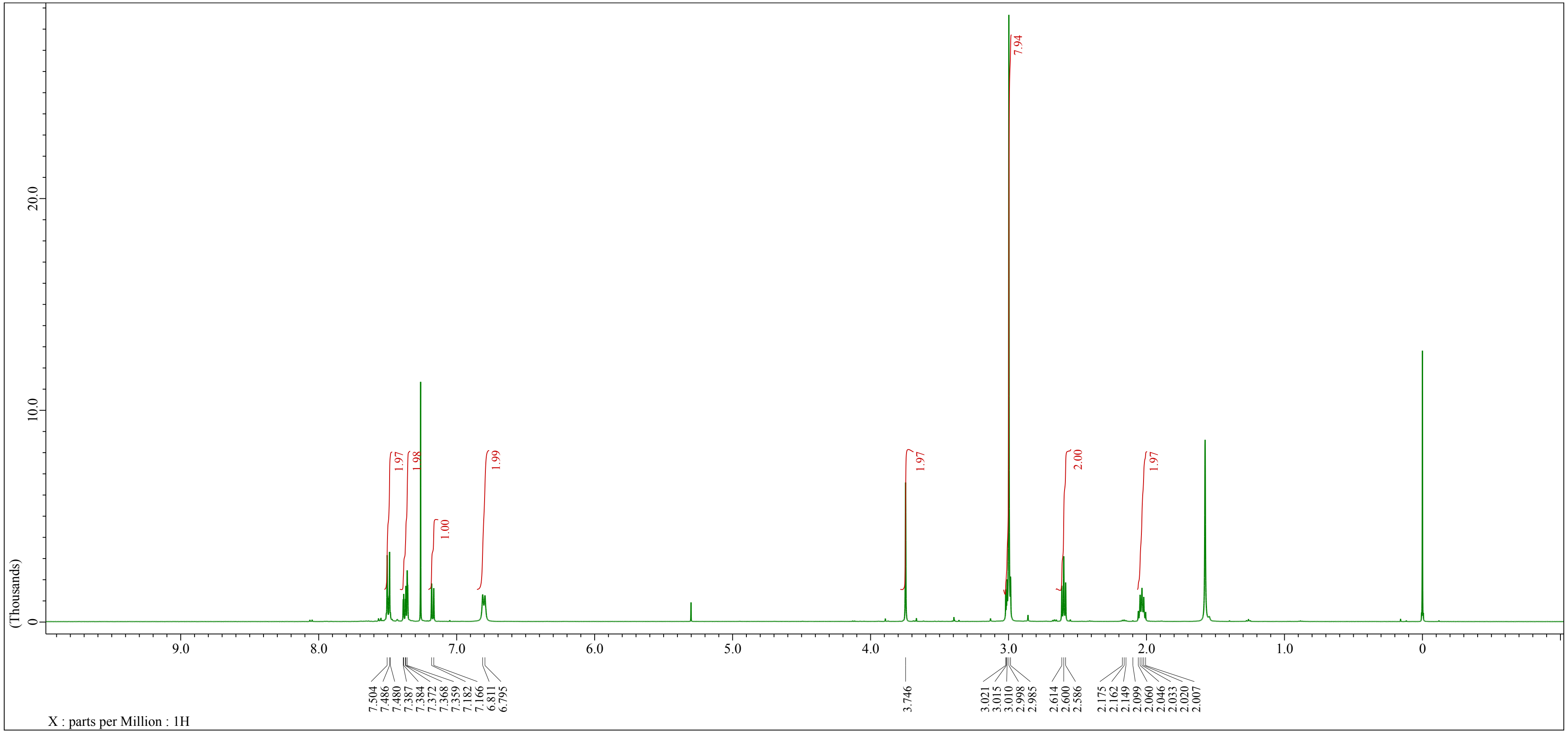

Figure S52. ^{1}H-NMR spectrum of **11b** (500 MHz, $CDCl_3$).

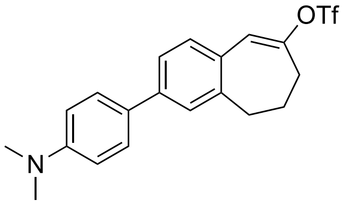

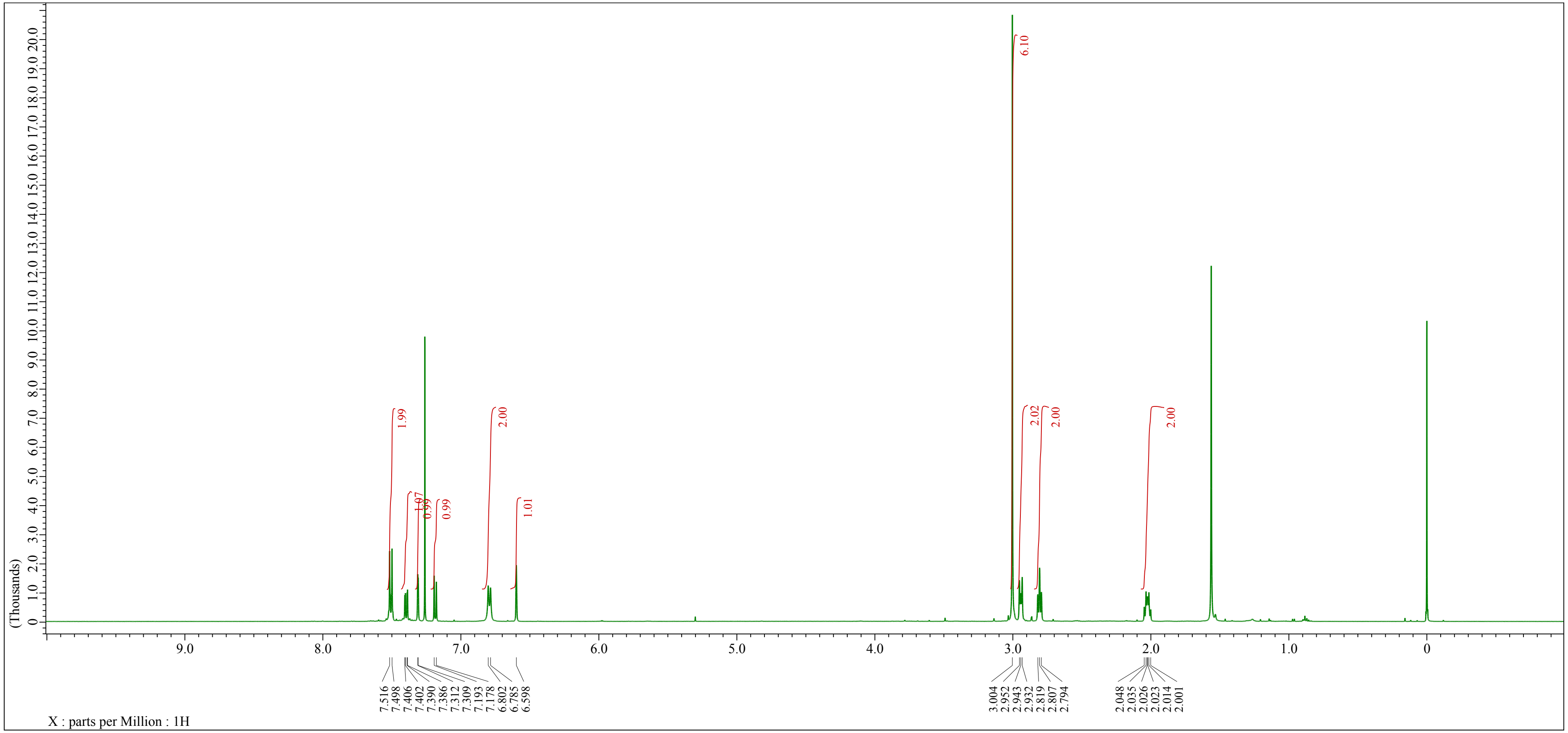

Figure S53. ^{1}H-NMR spectrum of **12b** (500 MHz, $CDCl_3$).

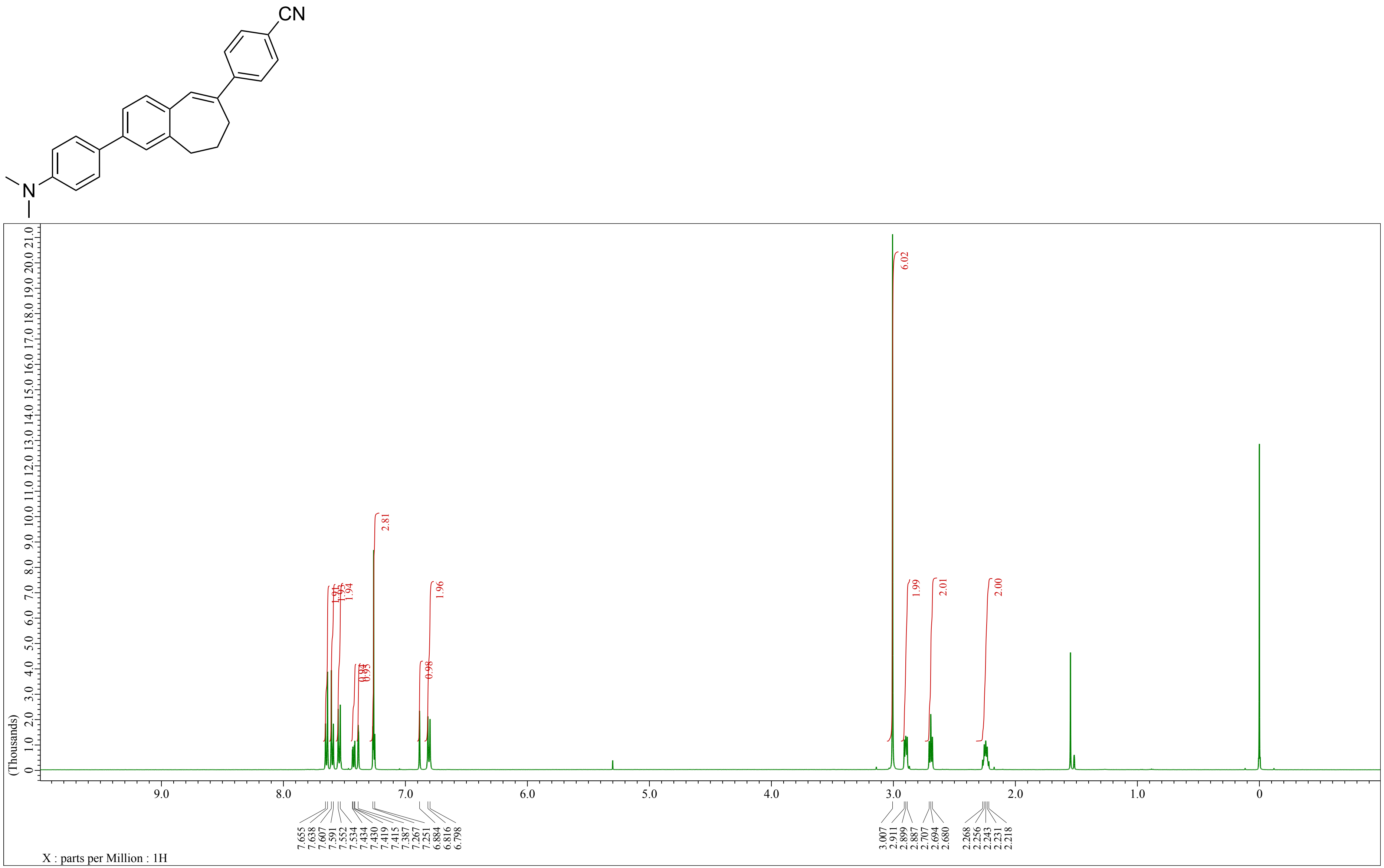

Figure S54. ^{1}H-NMR spectrum of **DPB[7]N** (500 MHz, $CDCl_3$).

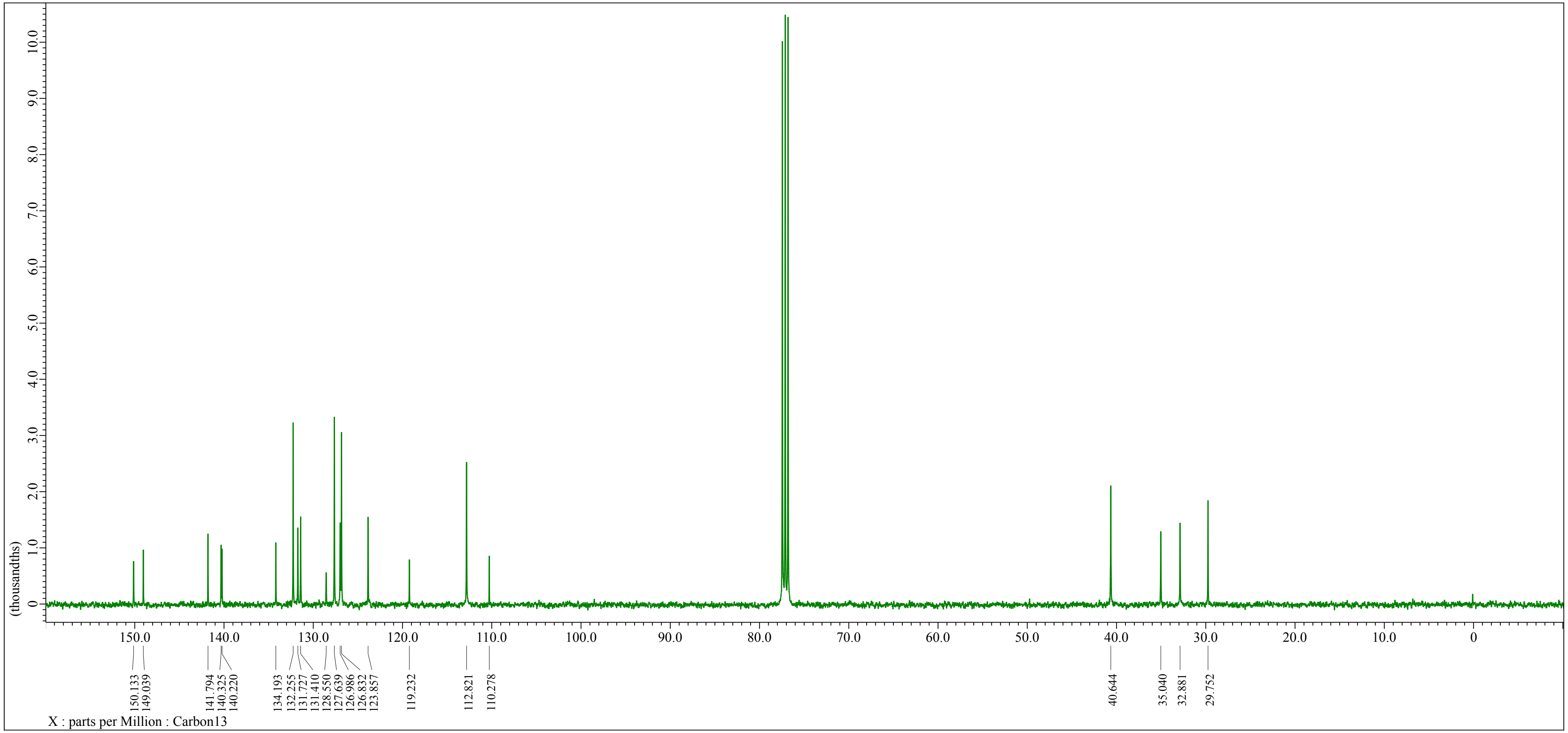

Figure S55. ^{13}C-NMR spectrum of **DPB[7]N** (100 MHz, $CDCl_3$)

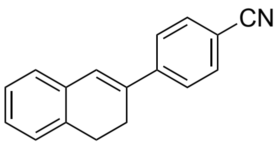

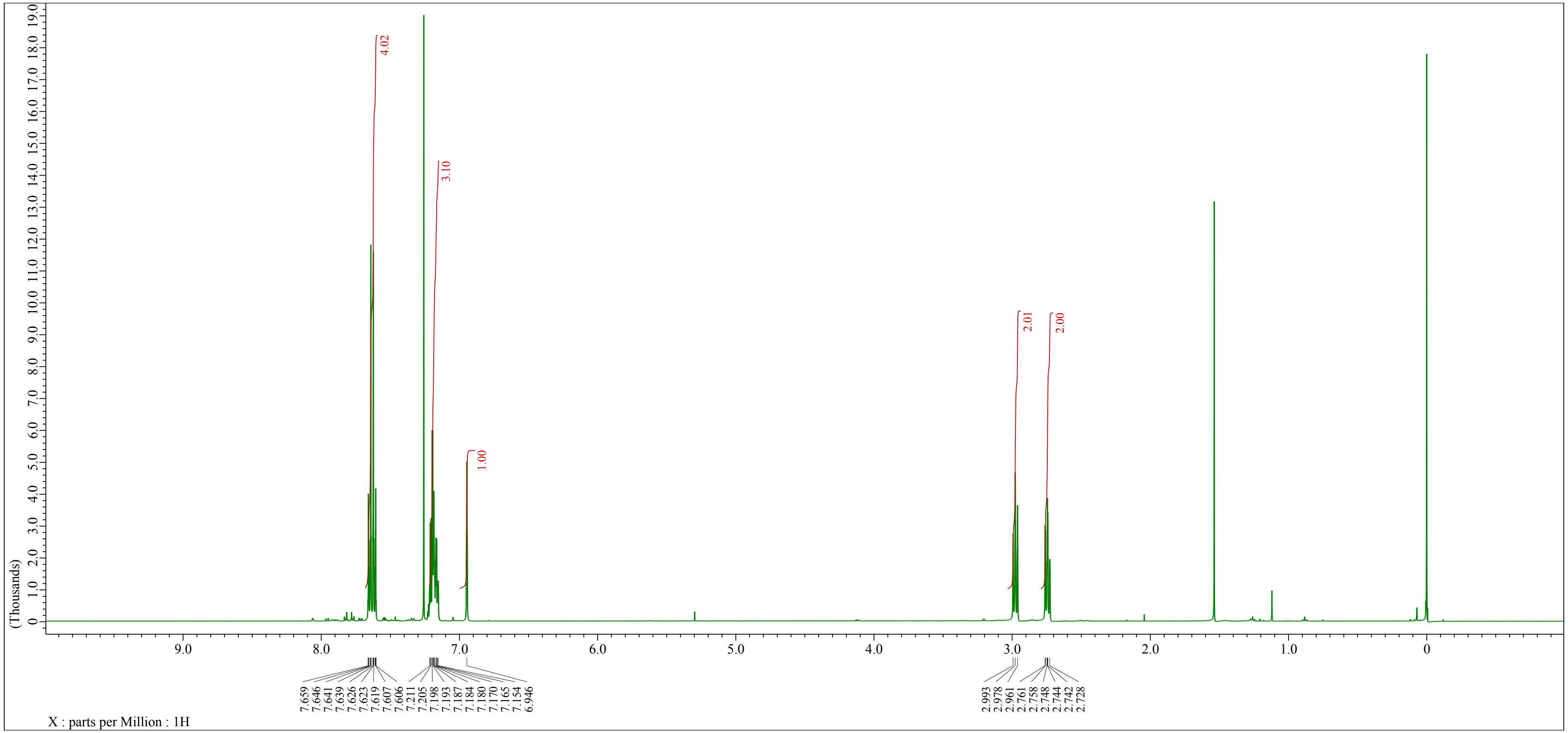

Figure S56. ^{1}H-NMR spectrum of **CpBS[6]** (500 MHz, $CDCl_3$).

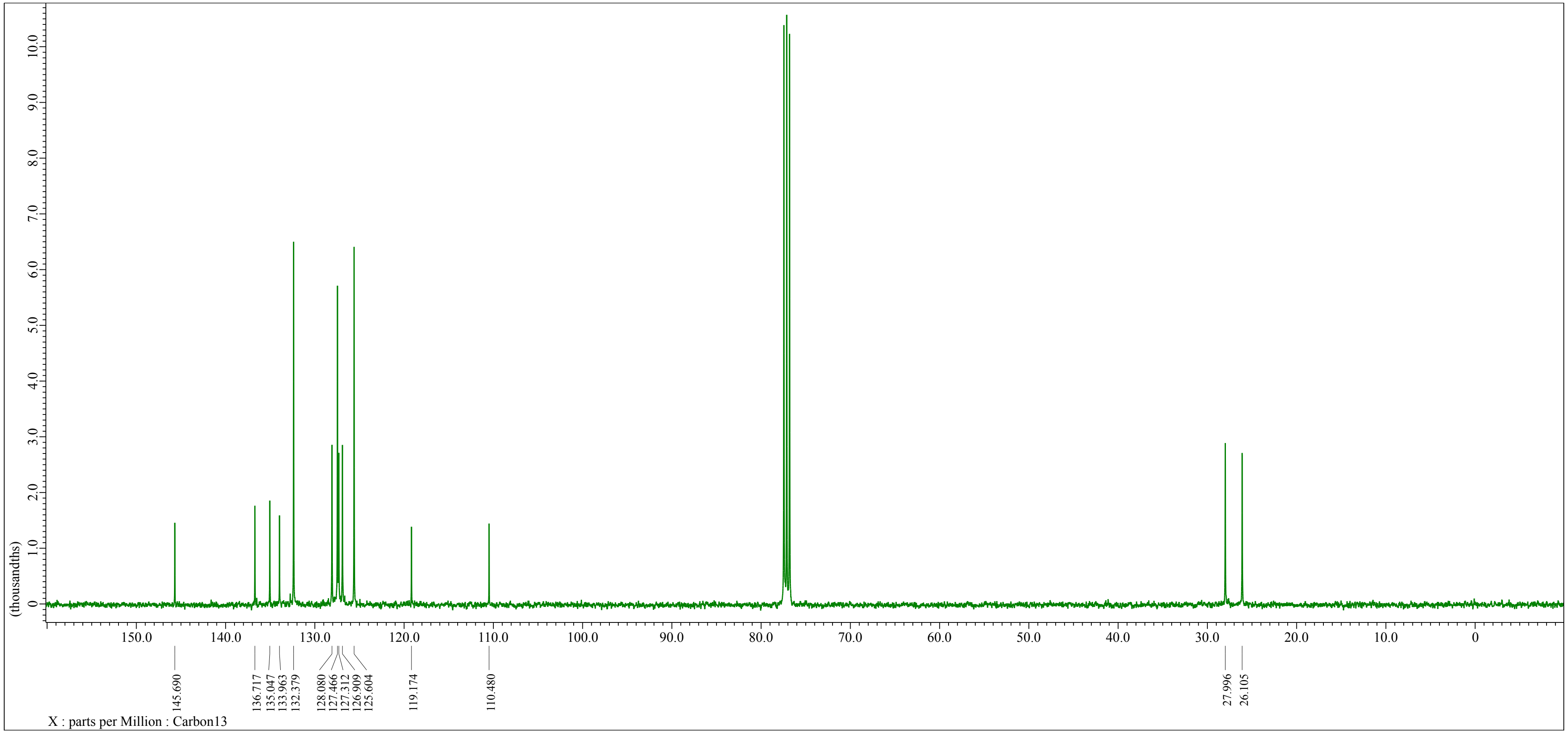

Figure S57. ^{13}C-NMR spectrum of **CpBS[6]** (100 MHz, $CDCl_3$).

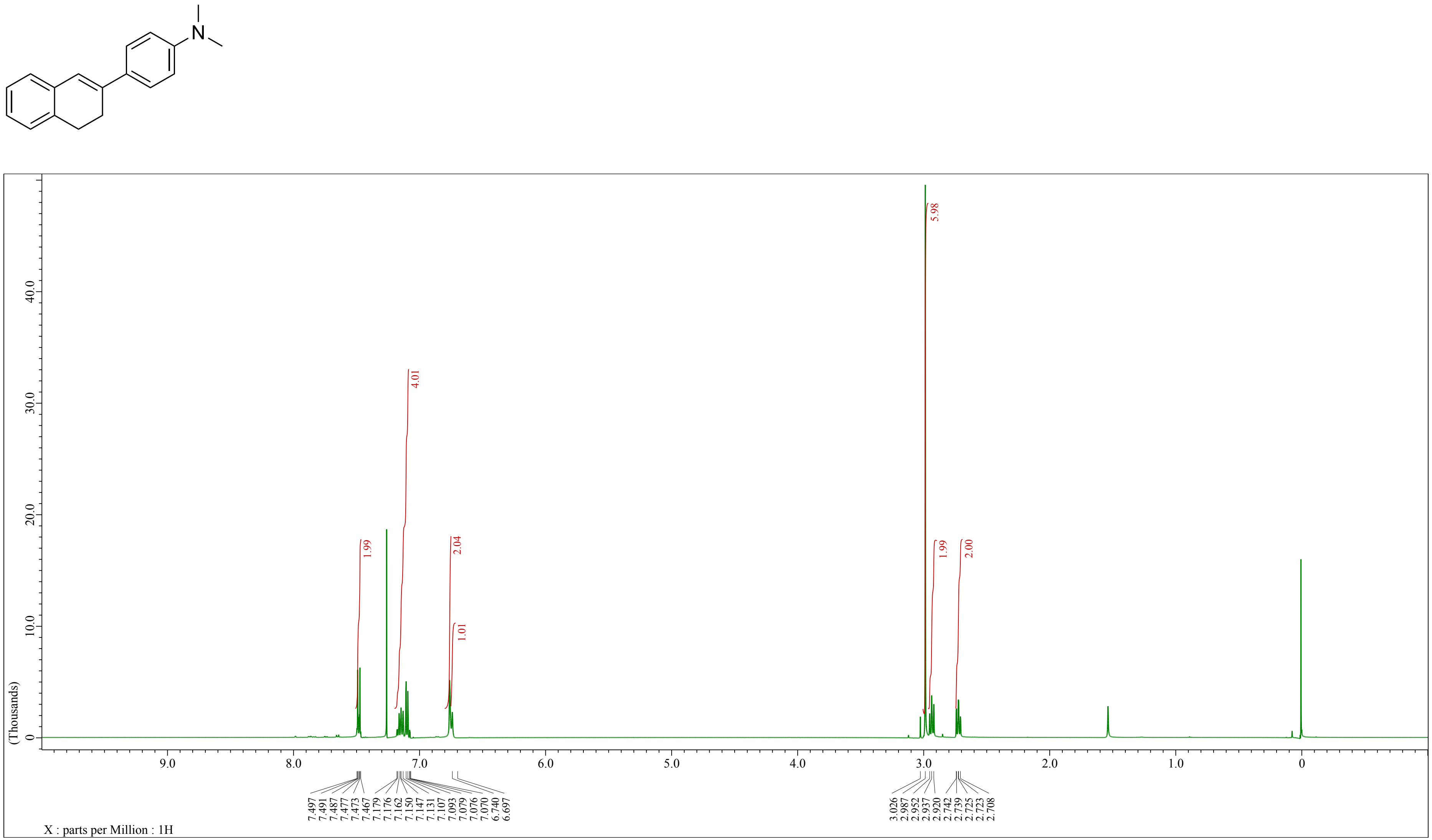

Figure S58. ^{1}H-NMR spectrum of **DBS[6]** (500 MHz, $CDCl_3$)

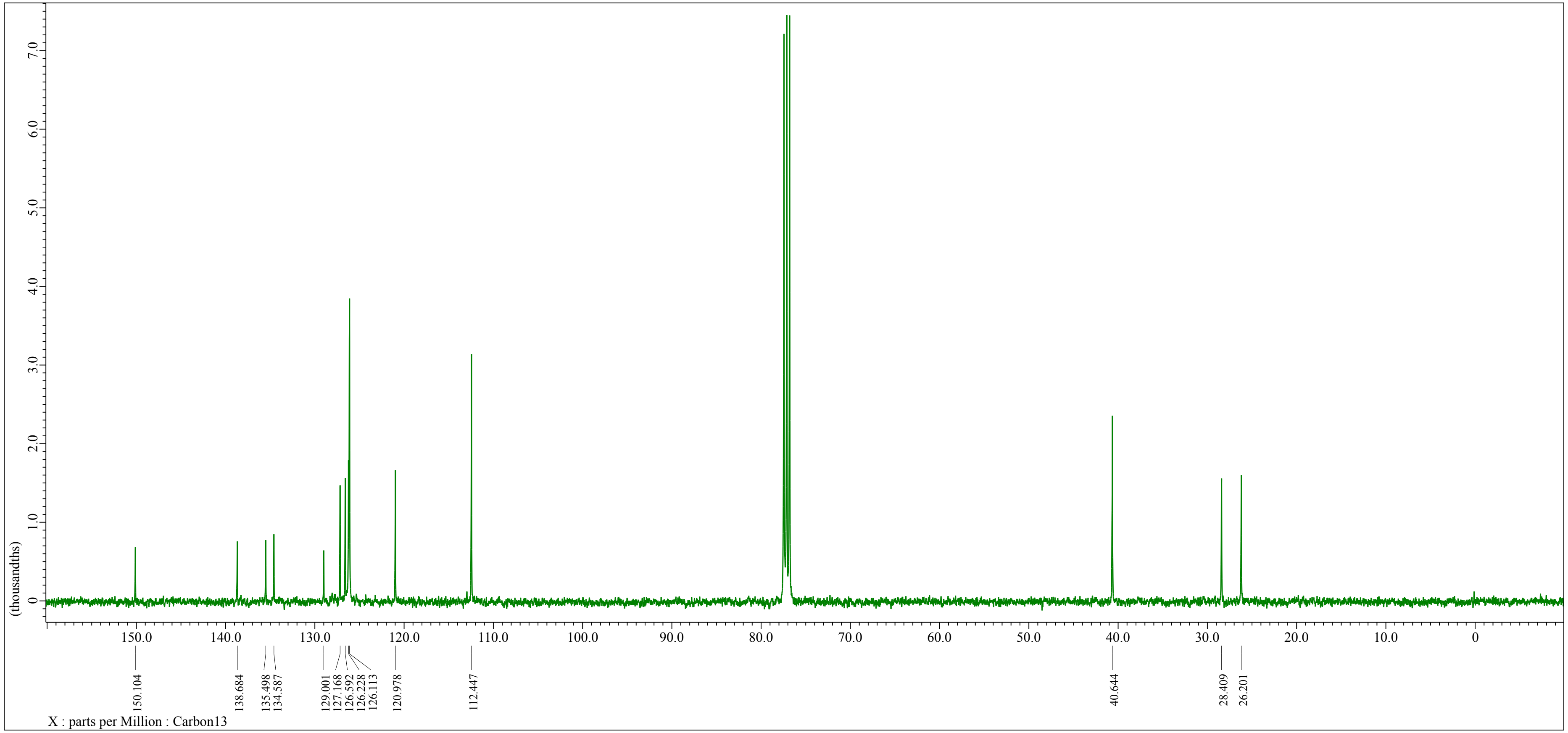

Figure S59. ^{13}C-NMR spectrum of **DBS[6]** (100 MHz, $CDCl_3$)

S8. Reference

S1. Gaussian 16, Revision C.01, M. J. Frisch, G. W. Trucks, H. B. Schlegel, G. E. Scuseria, M. A. Robb, J. R. Cheeseman, G. Scalmani, V. Barone, G. A. Petersson, H. Nakatsuji, X. Li, M. Caricato, A. V. Marenich, J. Bloino, B. G. Janesko, R. Gomperts, B. Mennucci, H. P. Hratchian, J. V. Ortiz, A. F. Izmaylov, J. L. Sonnenberg, D. Williams-Young, F. Ding, F. Lipparini, F. Egidi, J. Goings, B. Peng, A. Petrone, T. Henderson, D. Ranasinghe, V. G. Zakrzewski, J. Gao, N. Rega, G. Zheng, W. Liang, M. Hada, M. Ehara, K. Toyota, R. Fukuda, J. Hasegawa, M. Ishida, T. Nakajima, Y. Honda, O. Kitao, H. Nakai, T. Vreven, K. Throssell, J. A. Montgomery, Jr., J. E. Peralta, F. Ogliaro, M. J. Bearpark, J. J. Heyd, E. N. Brothers, K. N. Kudin, V. N. Staroverov, T. A. Keith, R. Kobayashi, J. Normand, K. Raghavachari, A. P. Rendell, J. C. Burant, S. S. Iyengar, J. Tomasi, M. Cossi, J. M. Millam, M. Klene, C. Adamo, R. Cammi, J. W. Ochterski, R. L. Martin, K. Morokuma, O. Farkas, J. B. Foresman, and D. J. Fox, Gaussian, Inc., Wallingford CT, 2019.
